# Digital Interventions for Generalized Anxiety Disorder (GAD): Systematic Review and Network Meta-Analysis

**DOI:** 10.3389/fpsyt.2021.726222

**Published:** 2021-12-06

**Authors:** Pedro Saramago, Lina Gega, David Marshall, Georgios F. Nikolaidis, Dina Jankovic, Hollie Melton, Sarah Dawson, Rachel Churchill, Laura Bojke

**Affiliations:** ^1^Centre for Health Economics, University of York, York, United Kingdom; ^2^Department of Health Sciences, University of York, York, United Kingdom; ^3^Hull York Medical School, University of York, Heslington, United Kingdom; ^4^Tees, Esk and Wear Valleys NHS Trust, Darlington, United Kingdom; ^5^Centre for Reviews and Dissemination, University of York, York, United Kingdom; ^6^IQVIA, London, United Kingdom; ^7^Common Mental Disorders Group, Cochrane Collaboration, York, United Kingdom

**Keywords:** worry, anxiety, cognitive behavior therapy (CBT), mobile applications, digital, systematic review (sr), meta-analysis

## Abstract

**Background:** Generalized anxiety disorder is the most common mental health condition based on weekly prevalence. Digital interventions have been used as alternatives or as supplements to conventional therapies to improve access, patient choice, and clinical outcomes. Little is known about their comparative effectiveness for generalized anxiety disorder.

**Methods:** We conducted a systematic review and network meta-analysis of randomized controlled trials comparing digital interventions with medication, non-digital interventions, non-therapeutic controls, and no intervention.

**Results:** We included 21 randomized controlled trials with a total of 2,350 participants from generalized anxiety disorder populations. Pooled outcomes using analysis of Covariance and rankograms based on the surface under the cumulative ranking curves indicated that antidepressant medication and group therapy had a higher probability than digital interventions of being the “best” intervention. Supported digital interventions were not necessarily “better” than unsupported (pure self-help) ones.

**Conclusions:** Due to very wide confidence intervals, network meta-analysis results were inconclusive as to whether digital interventions are better than no intervention and non-therapeutic active controls, or whether they confer an additional benefit to standard therapy. Future research needs to compare digital interventions with one-to-one therapy and with manualized non-digital self-help and to include antidepressant medication as a treatment comparator and effect modifier.

## Introduction

Generalized Anxiety Disorder (GAD) is the most common mental health condition with 6% point-prevalence (measured over the preceding week) in the UK, nearly double that of depression (3.3%) (1). It is often confused with panic disorder or depression when self-reported by survey participants (2). GAD is characterized by excessive worry that persists for several months and leads to significant distress or impairment in everyday life and functioning (3). Other typical characteristics include free-floating anxiety and physical symptoms, such as muscle tension, headaches, restlessness, difficulty concentrating, irritability, or sleep problems. GAD is associated with low quality of life and high healthcare costs (4).

Psychological interventions can be effective for GAD, especially cognitive behavior therapy (CBT) (5) and applied relaxation (6). CBT helps the individual challenge or tolerate worrying thoughts and confront anxiety-provoking situations rather than avoiding them. Applied relaxation counteracts the physical symptoms of GAD thought a series of tense-then-release muscle exercises that reduce muscle tension. Antidepressant medication can also be effective (7) and is often the first choice for treatment by clinicians in view of limited capacity to deliver psychological interventions. To improve access to psychological therapies and increase patient choice and therapist capacity, digital interventions have been used as alternatives or supplements to conventional face-to-face clinic-based therapy (8, 9).

Digital interventions are defined as software-based therapeutic activities accessed via technology platforms, such as the internet, virtual reality (VR), mobile phones. According to the World Health Organization (WHO), the term “digital intervention” represents a discrete function of using technology to achieve health sector objectives (10). In the context of a specific condition, such as GAD, digital intervention (DIs) fulfil the discrete function of using software and digital media to deliver therapeutic activities that aim to improve symptoms associated with the condition in populations.

An example of a DI for GAD is a 10-week internet-based self-help programme consisting of psychoeducation (information about worry, stress, and anxiety, including its risk factors and treatments), CBT (dealing with the purpose, meaning, and content of worry, as well as modifying unhelpful responses to worry), relaxation (tensing then releasing body muscle groups and refocus attention away from worry) and physical activity (11). Another example is a mobile app (12) that teaches diaphragmatic breathing in a series of mini-games, from sailing a boat down a river to flying balloons into the sky.

Previous reviews of the effectiveness of DIs for GAD (13, 14) included mixed populations of anxiety disorders and depression without reporting outcomes separately for GAD subgroups within these mixed samples. Reporting a disorder-specific outcome for mixed samples can be misleading because it implies that, if an intervention works for the mixed sample, it will also work for each of its constituent populations. Studies reporting findings from mixed samples do not answer the question of whether DIs are effective for GAD to inform disorder-specific clinical guidelines. To achieve this, and while preserving the benefits of randomisation, we need to analyse GAD outcomes reported separately for GAD populations and GAD sub-samples within mixed populations.

This paper reports a systematic review and quantitative synthesis of RCTs comparing DIs with other interventions, non-therapeutic control arms and no intervention for GAD populations with varying levels of illness severity (sub-threshold, mild, moderate, severe). The review had four objectives:

1. Categorize the DIs and comparator arms into groups that could be pooled together.
2. Compare the pooled outcomes of DIs with the pooled outcomes of non-digital interventions, medication, non-therapeutic controls, and no intervention for GAD symptoms.
3. Compare the pooled outcomes of different types of DIs.
4. Identify limitations and gaps in the existing research on DIs for GAD.

After describing the review, the classification and synthesis methodology in section Methods, section Results of the paper goes through the characteristics of the evidence base and synthesis results. Section Discussion discusses the findings, followed by concluding remarks in section Conclusions.

## Methods

### Protocol

The protocol for the review was registered with PROSPERO 2018 CRD42018105837 as part of a larger piece of work that investigated the costs and outcomes of digital interventions for mental health, funded by the UK's National Institute for Health Research (NIHR). The review has been conducted and reported as recommended by the Preferred Reporting Items for Systematic Reviews and Meta-Analyses (PRISMA) extension statement for network meta-analysis (15).

### Search Strategy

In December 2018, the following databases were searched to identify published and unpublished studies: MEDLINE, PsycINFO, Cochrane Central Register of Controlled Trials (CENTRAL), Cochrane Database of Systematic Reviews (CDSR), Cumulative Index to Nursing & Allied Health (CINAHL Plus), Database of Abstracts of Reviews of Effects (DARE), EMBASE, Web of Science Core Collection, NHS Economic Evaluation Database (NHS EED); Database of promoting health effectiveness reviews (DoPHER) and Proquest.

We also searched two clinical trial registries and other resources for ongoing studies: ClinicalTrials.gov and the WHO International Clinical Trials Registry Platform portal, as well as the NIHR portfolio of studies. Web searches were conducted using Google and Google Scholar making use of simplified search terms. After searches were complete, supplementary searches were conducted, including reference lists of included studies and forward citation searches. Finally, we contacted the authors of included studies for information on any other work in the field they were aware of. The searches were undertaken for studies conducted since 1997 and restricted to those written in English.

In June 2019, the searches were updated and widened to include terms based on unspecified Anxiety Disorders. An additional pilot search was conducted on Cochrane Library and PsycINFO databases using terms based on “worry” and “anxiety prevention.” This ensured that no articles were missed. No new included articles emerged from the pilot search, and it was not deemed necessary to expand it to all remaining databases. The full search terms and outputs of the database searches are provided in Appendix A (December 2018 search) and Appendix B (June 2019 search).

### Study Identification and Selection

Two reviewers (DM, HM) independently screened all titles and abstracts of the identified studies against our inclusion/exclusion criteria. If either reviewer indicated a study could be relevant, we retrieved the full text. The same two reviewers independently assessed the full texts against our inclusion/exclusion criteria. A third reviewer (LG) resolved any disagreements through discussion to agree a final list of included and excluded studies.

Eligible studies included: (a) study design: RCTs, to minimize risk of bias and confounding variables; (b) participants: participants with symptoms or risk of GAD within mental health populations or within the general population; we defined this as a certified diagnosis using a standardized diagnostic interview or a score above an accepted cut-off for diagnosable GAD (which may include sub-threshold scores) in standardized questionnaires; (c) interventions: software-based systems and technology platforms designed for patient-facing delivery of a mental health intervention (i.e., an intervention to improve mental health outcomes); (d) comparisons: all comparisons relevant to DIs, even when two or more DIs were compared with each other without other comparators; and (e) outcomes: GAD-specific measures of anxiety or worry (e.g., GAD-7), reported for GAD populations or GAD sub-samples within mixed populations.

We have excluded: (i) mixed populations of GAD with other conditions, when the outcomes were not reported separately for GAD subgroups; (ii) technology used as a means for telecommunication (e.g., email, phone or video) without any software-based processing; (iii) software-based systems designed for training of health professionals or for administration without any patient-facing intervention components; and (iv) studies that were only identified as protocols, abstracts, or reviews; these were marked so we could check for RCTs that we may have missed in the database searches.

### Data Extraction and Risk of Bias Assessment

Two researchers (DM, HM) independently extracted data from published and unpublished study reports. Data were extracted on the sample, study design, intervention, and comparator characteristics, baseline characteristics, and results. Any discrepancies were resolved by a third reviewer (LG). Risk of bias of each study was assessed using Cochrane's Risk of Bias (RoB) 2.0 (16).

### Classification of DIs and Their Alternatives

In order to conduct an evidence synthesis, it was necessary to classify and group the interventions from studies. We developed a classification system for DIs and their comparator interventions and controls, in the following four steps:

a) We conducted a detailed data extraction of intervention/control arm characteristics as reported within our included RCTs for GAD and their relevant linked papers.
b) We identified common and differentiating features of intervention/control arms between RCTs, but also incremental differences between interventions/controls within the same RCT.
c) We consulted the literature and an advisory group of health services researchers and clinicians about intervention features that could be important for clinical outcomes (e.g., amount of interpersonal contact, who offers support to the DI, whether the intervention is available publicly or *via* referral to specialist services, types of software required or therapeutic approaches used).
d) We applied the classification criteria to each randomisation arm in the included RCTs so that each intervention/control arm was assigned to a classification group.

These 4 steps were iterative; we resolved discrepancies by refining our classification criteria until two reviewers (DM, LG) independently reached the same allocation for every intervention/control they classified. We grouped DIs and their alternatives according to the three criteria below.

*Criterion 1–Intervention (I) or Control (C):* An Intervention (I) was an action carried out as part of a research protocol for therapeutic purposes, i.e., it was expected to improve clinical symptoms and functioning based on psychological or behavioral theories and preliminary evidence. A Control (C) was a non-therapeutic activity that was not expected to make a clinical difference to the condition; this could be a psychological placebo, an “attention control,” or a change in usual care introduced by the research team to keep participants safe and minimize attrition.
*Criterion 2–Digital (D) or Non-Digital (NoD)*: A Digital Intervention (DI) or a Digital Control (DC) included software programmes to guide patient-facing activities. A Non-Digital Intervention/Non-Digital Control (NoDI/NoDC) did not involve any technology and was delivered by printed materials or during face-to-face meetings, or *via* telecommunications technology without automated software e.g., consultations by email, skype, or phone.
*Criterion 3–Supported (S) or Unsupported (U)*: Supported interventions/controls included scheduled or regular two-way person-to-person contact (e.g., between service user and clinician or researcher, or peer-to-peer). Unsupported interventions/controls either had no interpersonal contact or included limited *ad-hoc* interaction (e.g., phoning a helpline with any problems as a one-off). We also classified as unsupported interventions/controls those in which communication was one-way, such as a reminders by email, post, or phone.

Based on these three criteria, we mapped DIs and their alternatives into eight groups resulting from the combinations of (I or C) x (D or NoD) x (S or U).

*Group 1: Supported Digital Intervention (SDI)*, e.g., computerized cognitive behavior therapy with phone support; clinician-delivered therapy assisted by virtual reality.
*Group 2: Unsupported Digital Intervention (UDI)*, e.g., internet self-help without any clinician contact, mobile app with automated reminders but without personal interaction.
*Group 3: Supported Non-Digital Intervention (SNoDI)*, e.g., individual or group therapy in a clinic, or therapy delivered by a clinician *via* phone or an online platform.
*Group 4: Unsupported Non-Digital Intervention (UNoDI)*, e.g., self-help using a treatment manual or a book or a website without clinician input.
*Group 5: Supported Digital Control (SDC)*, e.g., access to a general health education website with weekly check-in calls from a researcher; virtual reality “placebo” environment used in a clinic with a researcher present.
*Group 6: Unsupported Digital Control (UDC)*, e.g., access to an educational website without any support from a person or with just automated reminder emails.
*Group 7: Supported Non-Digital Control (SNoDC)*, e.g., weekly check-in by phone or regular clinical assessment face-to-face without any specific therapy instructions.
*Group 8: Unsupported Non-Digital Controls (UnoDC)*, e.g., printed materials with general health advice without any specific therapy instructions.

Separate groups were used for medication and no intervention. Medication (M) was any pharmacological agent (pills, injections, etc.) offered as part of a research protocol. No Intervention (NI) included waiting lists and usual care in which there were no additional therapeutic activities and no changes in patient routines. The NI group may still have received medication or consultations as part of routine care, but this would have been equally accessible to all participants in a trial irrespective of group allocation, so the effect would be canceled out across randomisation arms.

### Data Synthesis and Statistical Analysis

Over the last two decades, network meta-analysis (NMA) methods (17) also known as mixed treatment comparisons (18, 19)–have been developed to synthesize evidence from multiple studies. NMA is an extension to the standard (pairwise) meta-analysis, which pools together the results of studies for one type of intervention compared to one type of alternative (e.g., active treatment or placebo control). An NMA enables the simultaneous comparison of multiple interventions and multiple comparators within a single coherent analysis. Such an approach is routinely used in health technology assessments to inform the optimal intervention strategy for a given medical condition (20). NMAs are often used to inform estimates of clinical and cost-effectiveness and commissioning decisions.

In the NMA, an ANalysis of COVAriance (ANCOVA) modeling framework was used, where a final outcome measurement is synthesized and adjusted for baseline measurements. Compared to the “change from baseline” approach, the ANCOVA model avoids guessing within-patient correlation across measurements as typically this is not reported in studies. Treatment effect estimates based on ANCOVA methods have been shown to be more efficient, less biassed and robust to random baseline imbalance (21–26). Hence, the ANCOVA model, is the preferred method for estimating treatment effects from continuous outcomes (27–30).

We adopted a modeling approach in line with the parameterisation for continuous data with normal likelihood and identity link used by Dias et al. (21, 22). Fixed-effects (FE) and random-effects (RE) models (the latter accounting for potential correlation within multi-arm trials) were fit to the data. In the model, patients who did not receive any treatment were assumed to neither improve nor worsen over the duration (i.e., null placebo effect). Furthermore, it was assumed that the effect of the baseline measurement is common across all treatments, implying that when two active treatments are compared in a trial, the baseline effects are offset.

All analyses were conducted within a Bayesian Markov chain Monte Carlo (MCMC) approach, fitted using WinBUGS software version 1.4.3 [Copyright © 2007 Medical Research Council (UK) and Imperial College (UK)] (31) and linked to the freely available software R [version 4.0.2, Copyright © 2020 (32)] through the package R2WinBUGS (33). In all models the MCMC Gibbs sampler was initially run for 10,000 iterations and these were discarded as “burn-in.” Models were run for at least further 5,000 iterations, on which inferences were based. Chain convergence was checked using autocorrelation and Brooks-Gelman-Rubin diagram diagnostics (34–36). Goodness of fit and model complexity was assessed using the deviance information criterion (DIC) and posterior mean residual deviance (37).

We presented the estimated results as relative treatment effect scores and associated 95% credible intervals, CrIs. We have estimated the probability of a treatment being the “best” (i.e., being the most clinically effective) (38), and presented rankograms for all interventions, which provide the probabilities of an intervention being ranked 1 (the most effective) to 7 (the least effective). Finally, we reported the surface under the cumulative ranking curve (SUCRA), which is a numerical presentation of the overall ranking of each intervention. SUCRA values range from 0 to 100%, with higher SUCRA values suggesting that a treatment is likely to be better overall (21, 39).

Appendix C gives further details on the analysis (C1), including annotated synthesis WinBUGS code (C2), sample data and initial values for the main model used (C3).

### Assessment of Heterogeneity and Consistency

The model was extended to include study-level covariates as potential treatment effect modifiers. This meant that we looked for factors other than the treatment itself, which could have influenced outcomes within each study and may have created differences (heterogeneity) across studies. These factors included disease severity (40), concomitant medication (41) and the presence of comorbidities (42, 43). Meta-regression is the most commonly employed method to explore the influence of particular study-level covariates on the relative effect. To preserve all studies (and treatments), when a covariate was not reported by some studies, we allowed the model to impute missing covariate information (multiple imputation procedure assuming “missing at random”).

We assessed inconsistency to check that all pieces of evidence (from direct and indirect sources) were in agreement. Following guidance by Dias and colleagues (44, 45), inconsistency was assessed by comparing the DIC of our primary analyses (based on NMA models that assume consistency between direct and indirect evidence) and the DICs yielded by inconsistency models (which provide effect estimates based on direct evidence only). Results were assessed for coherence by qualitatively comparing estimates of pairwise ANCOVA meta-analysis (direct) and ANCOVA RE NMA (direct and indirect).

### Sensitivity Analysis

We conducted two types of sensitivity analysis. First, we evaluated the sensitivity of the networks to the influence of each individual trial. When network links were informed by more than one trial, we removed each trial one at a time (giving *nj*−*1* for each analysis, where *n* is the total number of trials in contrast *j*) and investigated the impact on the probability of each intervention being “best.” Second, we assessed the robustness of the synthesis results by repeating the analysis while excluding all studies of <30 patients.

## Results

### Included and Excluded Studies

**Figure 1:**
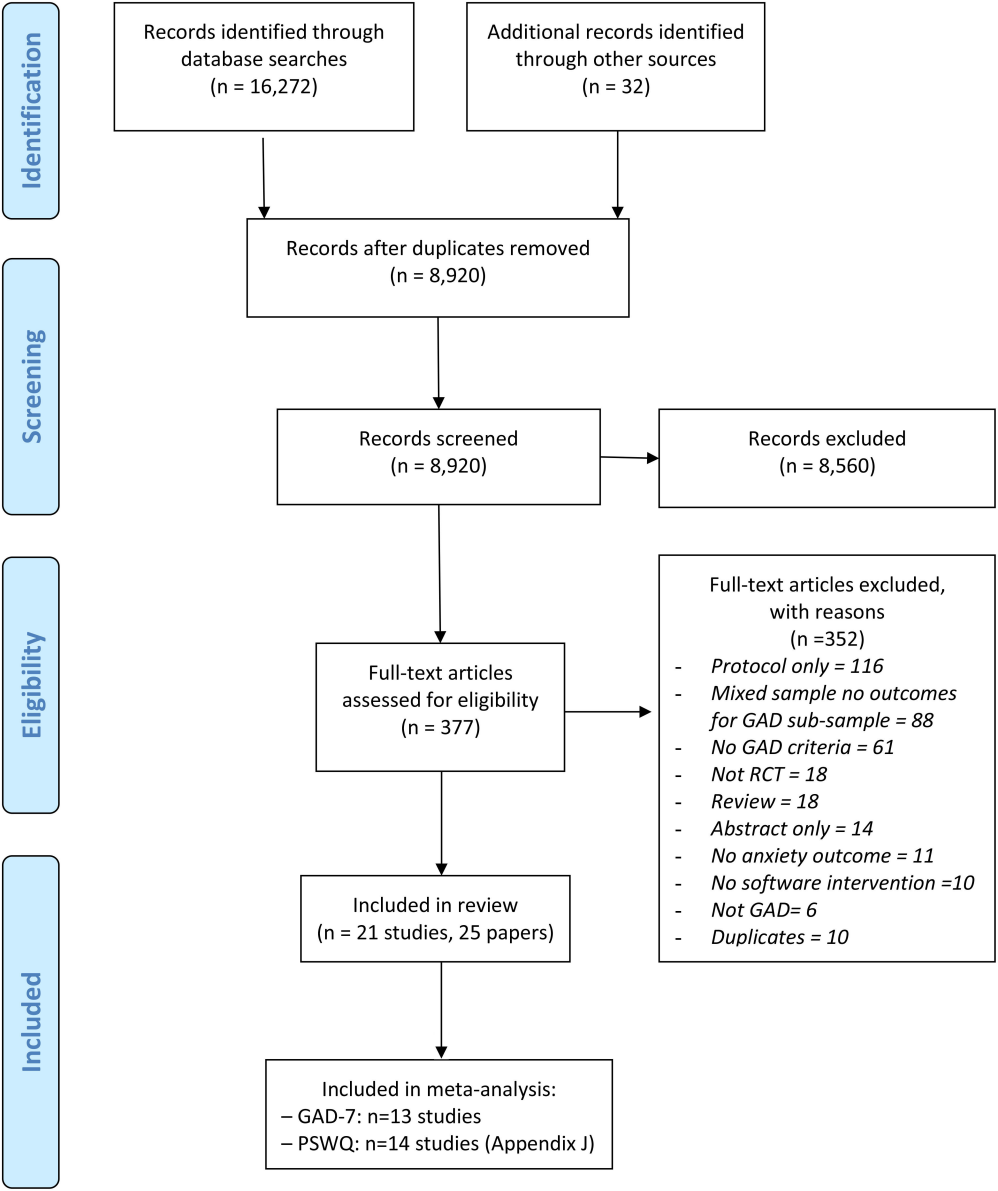
PRISMA diagram for the identification and selection of clinical trials relating to digital interventions for generalized anxiety disorder (GAD). Adapted from Moher (46).

Initial systematic searches of bibliographic databases identified 16,272 records; in addition, 32 records were identified through secondary searches (e.g., citation searching of protocols and abstracts). After duplicates were removed, a total of 8,920 records were screened by title and abstract and 8,560 records were excluded. We retrieved the full text papers for the remaining 377 records and, as a result of further screening, 352 articles were excluded. In total, 21 studies (reported in 25 papers) were included in the review. The PRISMA diagram (Figure 1) summarisez the number of records retrieved and selected at different stages of identification and screening. Appendix D gives a full reference list of the excluded studies grouped according to reasons for exclusion.

### Sample Characteristics in RCTs of DIs for GAD

The 21 RCTs included in the review, as detailed in Table 1, were conducted over 10 years between 2009 and 2019 in 10 countries (Sweden, Australia, USA, UK, Canada, Spain, Italy, Ireland, Taiwan, Netherlands) and involved 2,547 randomized participants. Most participants were recruited from the adult general population, except in four studies that recruited students/young adults and one study with over 60s. GAD populations were defined as either meeting the criteria of an established diagnostic tool, such as the Mini-International Neuropsychiatric Interview (MINI) (69), or a score above an accepted cut-off for diagnosable GAD in standardized questionnaires, such as the Generalized Anxiety Disorder-7 item questionnaire (GAD-7) (40).

**Table 1:** Sample characteristics and outcome measurement in RCTs of digital interventions for GAD.

**Study**	**Country**	**Population**	**Entry criteria**	***N* randomized**	***N* analyzed**	**Follow-up time points**	**Reported outcomes^**++**^**
Andersson et al. (47)	Sweden	General–adults	GAD diagnosis on SCID-I	81	59	8 weeks 3 months 18 months^*^	PSWQ, BAI. BDI-II, GADQ-IV, MADRS-S, QOLI, SCID-I, STAI-S, STAI-T
Andersson et al. (48)	Sweden	General–adults	PSWQ > 56	140	132	10 weeks 4 months^*^ 12 months^*^	PSWQ, BBQ, CAQ, HADS-A, IOU, MADRS-S, MCQ-30
Christensen et al. (11)	Australia	General–adults	GAD7 > 5	558	264	10 weeks 6 months 12 months	GAD-7, PSWQ, ASI, CES-D, days out of role, MINI
Christensen et al. (49)	Australia	General–adults	GAD diagnosis on ADIS-IV	21	11	10 weeks 6 months 12 months	GAD-7, CES-D, CGI
Dahlin et al. (50)	Sweden	General–adults	GAD diagnosis on SCID-I PSWQ > 45	103	85	9 weeks 6 months^*^	GAD-7, PSWQ, BAI, GADQ-IV, MADRS-S, PHQ-9, QOLI
Dear et al. (51)	Australia	General–adults	GAD diagnosis on MINI GAD7>5	338	260	9 weeks 3 months 12 months 24 months	GAD-7, K-10, MINI, MINI-SPIN, NEO-FFI-N, PDSS-SR, PHQ-9, SDS
Hazen et al. (52)	USA	University students	PSWQ > 60	24	23	3-6 weeks	PSWQ, BDI, STAI-T
Hirsch et al. (53)	UK	General–adults	Mixed sample anxiety/depression^**#**^ GAD diagnosis on SCID-I GAD7 > 10	64	64	3–4 weeks 1 month	GAD-7, PSWQ, PHQ-9, RRS
Howell et al. (54)	USA	University students	Mixed sample non-clinical (GAD <4) and clinical mild GAD (4 < GAD7 <10)^**~**^	197	NR	3 months	GAD-7**♢**
Johansson et al. (55)	Sweden	General–adults	Mixed sample anxiety/depression^**#**^ GAD diagnosis on MINI GAD7 > 10	43	NR	10 weeks 3 months	GAD-7, PHQ-9
Jones et al. (56)	Canada	Over 60s	GAD diagnosis or threshold sub-clinical on MINI GAD7>10	46	41	7–10 weeks 1 month	GAD-7, ACES, GAI, GDS, PHQ-9, PSWQ-A, WHOQOL
Navarro-Haro et al. (57)	Spain	Primary care–adults	GAD diagnosis on MINI	42	30	7–12 weeks	GAD-7, DERS, FFMQ, HADS, MAIA
Paxling et al. (58)	Sweden	General–adults	GAD diagnosis on SCID-I PSWQ > 53 GADQ-IV > 5.7	89	72	8 weeks 1 year^*^ 3 years^*^	PSWQ, BAI, BDI-II, GADQ-IV, MADRS-S, QOLI, STAI-S, STAI-T
Pham et al. (12)	UK	General–adults	Mixed sample common mental health problems^**##**^ GAD7 > 6 OASIS > 8 ASI > 16	63	42	4 weeks	GAD-7, Acceptability, ASI-3, OASIS, PDSS-SR, QLES-Q-SF
Repetto et al. (59) [linked with (60, 61)]	Italy	Primary care–adults	GAD diagnosis (unspecified tool used)	25	24	Not reported	GAD-7, PSWQ, BAI, HAM-A, STAI
Richards et al. (62)	Ireland	University students	GAD7>10	137	112	6 weeks	GAD-7, PSWQ, BDI-II, WASA
Robinson et al. (63)	Australia	General–adults	GAD diagnosis on MINI	150	138	11 weeks 3 months^*^	GAD-7, PSWQ, K-10, PHQ-9, SDS
Teng et al. (64)	Taiwan	General–adults	GAD diagnosis on DIS-IV PSWQ > 60	93	82	4 weeks 1 month	PSWQ, BAI, BDI, STAI-S, STAI-T
Titov et al. (65) [linked with (66)]	Australia	General–adults	GAD diagnosis on MINI	34	NR	9 weeks	GAD-7, K-10, PHQ-9, SDS
Titov et al. (67)	Australia	General–adults	Mixed sample anxiety/depression^**#**^ GAD diagnosis on MINI	48	19	9 weeks 3 months	PSWQ, DASS-21, K-10, NEO-FFI-N, PDSS-SR, PHQ-9, SDS, SPSQ
Topper et al. (68)	Netherlands	15- to 22-year-olds	PSWQ above 66th percentile (score 47)	251	218	8–10 weeks 3 months 12 months	PSWQ, BDI-II, EDI-2-BU, GADQ-IV, MASQ-D30, PTQ, QDS, RRS

* *Patients received active intervention at follow-up timepoint*.

*♢Study not included in the meta-analysis because it reported only categorical GAD-7 outcomes*.

*#Outcomes reported separately for GAD sub-group*.

*##Outcomes reported for the whole mixed sample of common mental health problems but all participants scored GAD7 > 6*.

*~Outcomes reported separately for mild GAD sub-group (4 < GAD7 < 10)*.

*++ For the full measure names corresponding to the acronyms, see Appendix E*.

### Risk of Bias Assessment

All but one (52) out of the 21 included studies, were judged to have a high risk of bias in at least one domain of assessment, for at least one outcome measure. This was largely due to the choice of outcome measurement as all studies used self-reported–albeit standardized–questionnaires. Self-reported outcomes are considered to have a high risk of bias in these studies because participants can rarely be blind to their allocation group. A visual description of the results of the RoB assessment is given in Appendix E, both for each RoB domain across all studies (Appendix E1) and for each study under each RoB domain (Appendix E2).

### Classification of Digital Interventions and Comparators

A classification exercise took place to enable consistency across digital interventions and comparators. We classified DIs and their alternatives according to three criteria: (a) whether they were a psychological/behavioral intervention (I) or a non-therapeutic psychological/behavioral control (C); (b) whether they were digital (D) or non-digital (NoD); (c) whether they were supported (S) or unsupported (U). Waiting lists and usual care were classified under no intervention (NI) unless an active component (e.g., monitoring, sham activity) was introduced, in which case the waiting list/usual care was classified as non-therapeutic psychological/behavioral control. An additional classification group was included for pharmacological interventions, called medication (M).

The interventions and controls of the 20 RCTs were allocated to one of the following eight classification groups: medication (M); no intervention (NI); supported digital control (SDC); supported digital intervention (SDI); supported non-digital control (SNoDC); supported non-digital intervention (SNoDI); unsupported digital control (UDC); unsupported digital intervention (UDI). There were no available clinical studies that included unsupported non-digital interventions (UNoDI) or unsupported non-digital controls (UNoDC). Table 2 describes all the interventions and controls included in each classification group for each study.

**Table 2:** Characteristics and classifications of interventions and controls in RCTs of digital interventions for GAD.

**Study**	**Intervention or control description (mode of delivery, therapy/control method, type of interpersonal contact/support)**	**Classification**
Andersson et al. (47)	1. Web-based psychodynamic therapy + weekly online support by psychology students/qualified psychologist	SDI
	2. Web-based CBT + weekly online support by psychology students/qualified psychologists	SDI
	3. Waiting list (crossover to web-based CBT at 3-months).	NI
Andersson et al. (48)	1. Web-based extinction therapy + daily online support by psychology students	SDI
	2. Waiting list (+ weekly online^#^ PSWQ ratings and option to phone if symptoms worsen–crossover to web-based extinction therapy at 10 weeks)	NI^a^
Christensen et al. (11)	1. Web-based CBT–no interpersonal communication	UDI
	2. Web-based CBT + weekly phone calls by “casual interviewers”	SDI
	3. Web-based CBT + weekly reminder email similar in content to phone calls by “casual interviewers” but no two-way communication	UDI
	4. Control website (information about general health)–no interpersonal communication	UDC
	5. Control website (information general health) + weekly phone calls by “casual interviewers”	SDC
Christensen et al. (49)	1. Web-based CBT + scheduled on-site meetings with psychologists/GPs	SDI
	2. Control website (information about general health) + scheduled meetings with psychologists/GPs	SDC
	3. Medication (SSRI–Sertraline 25 up to 100 mg per day) + scheduled meetings with psychologists/GPs	M
Dahlin et al. (50)	1. Web-based MBT & ACT + weekly messages via a secure messaging system by psychology students	SDI
	2. Waiting list (+weekly online^#^ GAD-7 and PSWQ ratings–contact with administrator implied for weekly measure completion but unclear–crossover to modified web-based MBT & ACT at 9 weeks)	NI^a^
Dear et al. (51)^*^	1. Web-based CBT (trans-diagnostic model focusing on mental wellbeing) + weekly phone/email contact with qualified psychologists	SDI
	2. Web-based CBT (GAD-specific focusing on worry control) + weekly phone/email contact with qualified psychologists	SDI
	3. Web-based CBT (trans-diagnostic model focusing on mental wellbeing) + standardized weekly email reminders and option to phone/email if needed technical support or had other problems–no scheduled or regular interpersonal contact	UDI
	4. Web-based CBT (GAD-specific focusing on worry control) + standardized weekly email reminders and option to phone/email if needed technical support or had other problems–no scheduled or regular interpersonal contact	UDI
Hazen et al. (52)	1. Computer-delivered attentional retraining + “non-therapy” meetings with “experimenters” every 6 days	SDI
	2. Sham training + “non-therapy” meetings with “experimenters” every 6 days	SDC
Hirsch et al. (53)^**^	1. Web-based CBM + 1 initial onsite meeting + regular (unspecified) contact by phone/email/SMS with researchers (unspecified qualifications) + RNT priming	SDI
	2. Web-based CBM + 1 initial onsite meeting + regular (unspecified) contact by phone/email/SMS with researchers (unspecified qualifications)–no RNT priming	SDI
	3. Control website (neutral scenarios) + 1 initial onsite meeting + regular (unspecified) contact by phone/email/SMS with researchers (unspecified qualifications)	SDC
Johansson et al. (55)	1. Web-based psychodynamic therapy + weekly written messages via online messaging system by therapists (unspecified qualifications)	SDI
	2. Waiting list + weekly assessment and non-directive support via online messaging system with therapists (unspecified qualifications) matching therapist support in the intervention	SDC^b^
Jones et al. (56)	1. Web-based CBT + weekly messages via online messaging system by therapists (unspecified qualification)	SDI
	2. Waiting list (crossover after 7– 10 weeks)–no monitoring or other input specified	NI
Navarro-Haro et al. (57)	1. Group MBI in weekly onsite meetings with a therapist	SNoDI
	2. VR mindfulness skills + group MBI in weekly onsite meetings with a therapist	SDI
Paxling et al. (58)	1. Web-based CBT (like an online book) + weekly online/email contact with therapist	SDI
	2. Waiting list (crossover after 8 weeks)–no monitoring or other input specified	NI
Pham et al. (12)	1. Mobile game of breathing re-training–no interpersonal contact	UDI
	2. Waiting list + weekly newsletter with curated content on breathing retraining exercises, matching content to mobile game, mindfulness meditation (assumed via mobile but not clear) + email reminders to complete assessments (crossover to access the mobile game after 4 weeks)	UDC^c^
Repetto et al. (59)	1. VR relaxation during weekly meetings with therapist + mobile phone home-access of VR environments	SDI
	2. VR relaxation during weekly meetings with therapist + mobile phone home-access ofVR environments + biofeedback machine for therapist to adapt VR environments according to participant heart rate	SDI
	3. Waiting list (no monitoring or any other input specified)	NI
Richards et al. (62)	1. Web-based CBT + weekly online messages by psychologists	SDI
	2. Waiting list (crossover at week 7–no monitoring or other input)	NI
Robinson et al. (63)	1. Web-based CBT + weekly phone/email contact by a “clinician” (clinical psychologist)	SDI
	2. Web-based CBT + weekly phone/email contact by a “technician” (administrative clinic manager)	SDI
	3. Waiting list (crossover at week 11–no monitoring or other input)	NI
Teng et al. (64)	1. Mobile app–home-delivered ABM + weekly “lab” meeting with assistant + phone call if missed sessions	SDI
	2. Mobile app–attention training + weekly “lab” meeting with assistant + phone call from if missed sessions	SDC
	3. Waiting list + weekly meetings with research assistant for matching assessment in a “lab”	SNoDC^d^
Titov et al. (65)	1. Web-based CBT + moderated online discussion forum + instant online messaging + 1 initial phone contact + subsequent email/phone weekly contact with clinical psychologist	SDI
	2. Waiting list (crossover at week 11–no monitoring or other input)	NI
Titov et al. (67)	1. Web-based CBT + moderated online discussion forum + instant online messaging + 1 initial phone contact + subsequent email/phone weekly contact with clinical psychologist	SDI
	2. Waiting list (+ unclear if contact with psychologist–crossover at week 9)	NI
Topper et al. (68)	1. Group CBT in weekly meetings with psychologists	SNoDI
	2. Web-based CBT + weekly online personalized feedback from psychologists (unclear whether there was two-way communication between participant and psychologist in response to feedback).	SDI
	3. Waiting list (crossover at 12 months–no monitoring or other input from the research team)	NI

*# Not clear whether online measures were sent by email or were automated and delivered via a platform*.

* *2 x 2 factorial RCT, so outcomes reported in 2 groups (SDI vs. UDI for both GAD-specific and transdiagnostic groups)*.

** *Outcomes reported as a single group across both interventions (SDI with or without RNT)*.

^***^Study not included in the meta-analysis because it only reported GAD-7 categorical outcomes.

a *“Waiting list” classified as “No Intervention” (NI) because the online weekly ratings were self-completed without any further input from the research team*.

b *“Waiting list” classified as “Supported Digital Control” (SDC) because there was substantial and regular non-specific support and monitoring via an online messaging system with a therapist that matched the duration of therapeutic support in the intervention arm*.

c *“Waiting list” classified as Unsupported Digital Control (UDC) because there was substantial and regular information and therapeutic advice similar to that of the intervention –via the mobile (inferred) (hence “digital”); standardized materials without two-way interaction with a therapist (hence “unsupported”)*.

d *“Waiting list” classified as Supported Non-Digital Control (SNoDC) because there was substantial and regular face-to-face assessments with a researcher that matched the assessments of the intervention groups*.

*ABM, Attentional Bias Modification; ACT, Acceptance and Commitment Therapy; CBT, Cognitive Behavior Therapy; GAD, Generalized Anxiety Disorder; M, medication; MBI, Mindfulness Based Intervention; MBT, Mindfulness Based Therapy; NI, No intervention; PSWQ, Penn State Worry Questionnaire; RNT, Repetitive Negative Thinking; SDC, Supported Digital Control; SDI, Supported Digital Intervention; SNoDC, Supported Non-Digital Control; SNoDI, Supported Non-digital intervention; SSRI, Selective Serotonin Reuptake Inhibitor; VR, Virtual Reality; UDC, Unsupported Digital Control; UDI, Unsupported Digital Intervention*.

Based on the 8-group classification for GAD RCTs, the majority of DIs studied were supported (SDI−18 RCTs) and were compared against no intervention (NI−12 RCTs). Only 3 RCTs evaluated unsupported DIs (UDI); two were web-based CBT (11, 51); and one (Pham et al.) a mobile game to practice breathing re-training. The only non-digital intervention (NoDI) represented in two RCTs (57, 68) was group therapy (one CBT and one mindfulness-based intervention) and there was only one RCT that included an antidepressant medication, Sertraline (49). With regards to non-therapeutic active controls reported in 8 RCTs, most included a digital element whereas only one RCT (64) had a non-digital control in the form of a weekly face-to-face assessment with a research assistant in a lab (SDC).

Just over half of the included RCTs (12/21) evaluated CBT. Therapeutic approaches other than CBT included: psychodynamic therapy (47), extinction therapy (48), acceptance and commitment therapy (50), cognitive or attentional bias modification (52, 53, 64), mindfulness (50, 57), relaxation (Repetto, 52), and diaphragmatic breathing (12). The most common technology platform use was a web-interface (*n* = 17). Two studies used VR platforms (57, 59), and two used smartphone apps (12, 64). The study by Repetto et al. (59) also used a mobile interface to enable users to access the VR scenarios at home but has also included a biofeedback system in one of the study arms.

DIs differed not only in technology platform and therapy type, but also in whether additional interpersonal support was offered as an adjunct to the digital element and, if so, its type. Most DIs included some interpersonal contact by phone or face-to-face with professionals (GPs, therapists, psychologists both students and qualified), non-clinical researchers, or lay people. Only two studies included DIs that were pure self-help without any contact (11, 12). Some DIs were supplemented by standardized emails without regular communication with another person (11, 51, 54).

### Selection of Studies and Outcome for the NMA

A total of 45 different outcome measures were reported in the included RCTs, as shown in Appendix F. GAD-7 was used in 14 out of the 21 RCTs to measure symptoms at baseline and outcomes at follow-up. Penn State Worry Questionnaire (PSWQ) (70, 71) was also reported in 14 studies. Apart from GAD-7 and PSWQ, the two other most frequently reported outcomes were for depression: the Patient Health Questionnaire−9 item (PHQ-9) (72) and the Beck Depression Inventory–version II (BDI-II) (73), reported in 8 and 6 RCTs, respectively (Appendix F1). The Hamilton Anxiety Scale (HAM-A) (74) used in a recent NMA on medication for GAD (7), only appeared once in the included RCTs (Appendix F2).

We focused on GAD-7 as our outcome of choice for the NMA. GAD-7 is a 7-item anxiety scale described in the literature as a valid and efficient tool to screen for GAD and assess symptom severity in clinical practice and research (40).

Our NMA for GAD-7 included 13 studies (Table 1). One study (54) used GAD-7 but it was not included in the meta-analysis because it only reported categorical outcomes (i.e., mild, moderate, severe) rather than continuous scores. The measurement period across studies ranged from 3 to 12 weeks, with longer follow-ups only available for very few studies.

Given the high level of reporting of PSWQ, data for this outcome was also quantitatively synthesized (see Appendix J).

### NMA Results for GAD-7 Scores at Follow-Up

Ten direct treatment comparisons were made in the 13 trials included in the GAD-7-based NMA; 4 of the 13 trials were multi-arm trials [three 3-arm trials (49, 59, 63) and one 5-arm trial (11)]; five comparisons were informed by more than one trial where pair-wise ANCOVA meta-analysis was conducted (ANCOVA FE models and ANCOVA RE for when *n* > 3).

**Figure 2:**
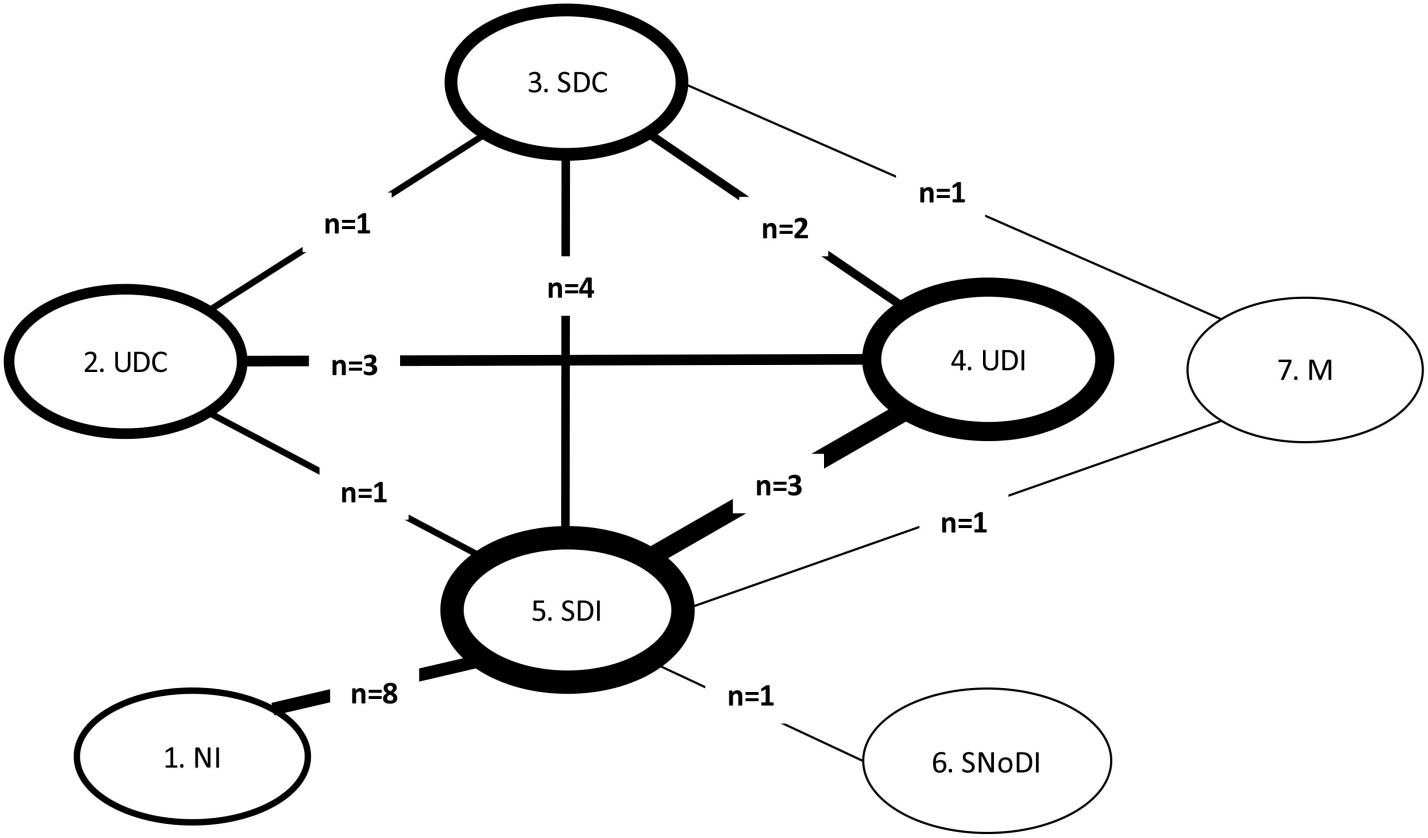
Network plot for comparisons between all interventions and controls for GAD populations in RCTs with GAD-7 score. GAD, Generalized Anxiety Disorder; M, medication; NI, No intervention; SDC, Supported Digital Control; SDI, Supported Digital Intervention; SNoDI, Supported Non-digital intervention; UDC, Unsupported Digital Control; UDI, Unsupported Digital Intervention. Line thickness around the node: proportional to the number of patients contributing to each intervention/control group. Line thickness connecting nodes: proportional to the number of patients contributing to each pairwise comparison between interventions/controls. n, number of trials informing each comparison.

We constructed a network plot to illustrate which interventions had been compared head-to-head (direct pairwise comparisons) for GAD-7 within the 13 included RCTs. An overview of these pairwise comparisons and synthesized data are shown in Appendix G. The structure of the network for GAD-7 is shown in Figure 2.

Fixed- and random-effects models were employed with minimal difference in mean residual deviances and DIC identified between the models tested. However, posterior estimates of between-study heterogeneity, τ^2^, suggested considerable variability across studies, which was in line with the narrative assessment of the studies. Hence, a random-effects approach was preferred. There was a high degree of uncertainty in the network results, especially in links not informed by direct evidence. Table 3 presents the full results of the NMA based on GAD-7 scores.

**Table 3:** Full meta-analysis results: network and direct pairwise comparisons between all interventions and controls for post-treatment (12 weeks) GAD-7 scores adjusted for baseline.

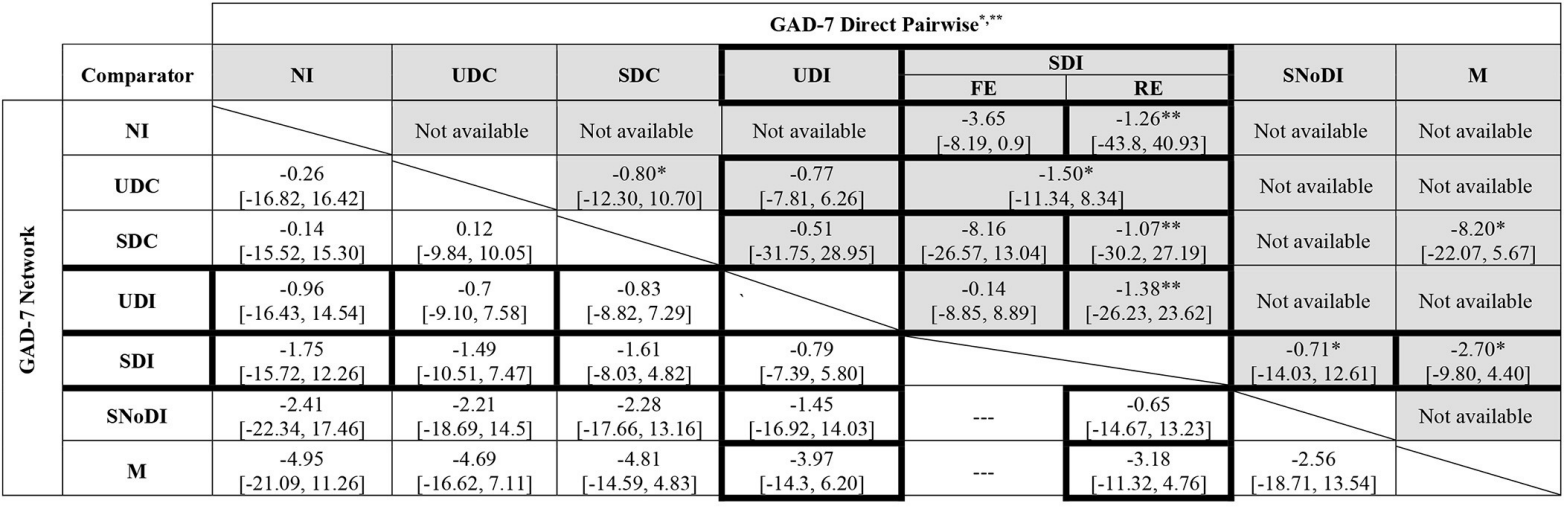

*FE, Fixed Effects; GAD, Generalized Anxiety Disorder; M, medication; NI, No intervention; RE, Random Effects; SDC, Supported Digital Control; SDI, Supported Digital Intervention; SNoDI, Supported Non-digital intervention; UDC, Unsupported Digital Control; UDI, Unsupported Digital Intervention*.

^*^Non-pooled data for when n = 1.

^**^Pairwise ANCOVA RE meta-analysis for when n > 3 for contrasts with intervention SDI.

*Lower left triangle: Network Meta-Analysis Results (ANCOVA RE); Upper right triangle (shaded area): Direct Pairwise Meta-Analysis Results (ANCOVA FE); Cells with thick black perimeter: available comparisons between digital interventions (supported and unsupported) and alternatives*.

Medication (M) was associated with the largest decrease in GAD-7 median scores compared to the other interventions, although uncertainty was high in the NMA estimates, with all 95% credible intervals including zero. These results are driven by the outcomes of a small (*n* = 21), three-arm, trial (49) that compared medication supplemented with scheduled face-to-face meetings with psychologists and GPs, against SDC (a general health website with scheduled meetings with psychologists & GPs) and SDI (a web-based CBT self-help programme with scheduled meetings with psychologists & GPs). The adjustment for baseline scores indicated that the baseline effect on the final outcome is small with a 95% credible interval including zero [change in GAD score: −0.14 (95% CrI −1.10 to 0.82)].

Results of independently pooling direct evidence for each contrast (but not pooling when *n* = 1) were found to be generally consistent with the NMA results, both in terms of direction and magnitude of the estimates (Table 3, upper-right triangle, shaded). Of note are the differences in the estimates found when applying fixed- and random-effects ANCOVA meta-analysis model on direct evidence for the comparisons of SDIs vs. SDCs (*n* = 4) and SDIs vs. NI (*n* = 8), evidencing non-negligible variability across studies and the importance of accounting for between-study heterogeneity.

Based on SUCRA values and rankograms for each intervention, as detailed in Appendices H1, H2, respectively, SDIs were estimated to be more effective (i.e., ranked higher) than UDIs, which included unsupported web-based CBT (11, 51) and an unsupported mobile breathing retraining game (12); however SDIs were less effective than SNoDI, a weekly group mindfulness-based intervention with a therapist (57).

Similar analysis was performed on the PSWQ outcome. Results are shown in Appendix J.

### Results of Between-Study Heterogeneity and Inconsistency Assessments

Three sources of heterogeneity were considered relevant: disease severity, concomitant medication, and comorbidities. Using data relating to disease severity and comorbidities was not feasible–see Appendix I for further details–thus only data on concomitant medication was included as a covariate in the synthesis modeling. When this covariate is included, the between-study heterogeneity parameter, τ^2^, is not reduced, suggesting that heterogeneity is not explained by this covariate. Crucially, even if the proportion receiving concomitant medication was identified as an important effect modifier, the meta-regression model is not necessarily suited to detect this intervention-covariate interaction as patients were receiving medication before trial entry. Therefore, medication may have already exerted an effect on patients, being captured by the ANCOVA baseline adjustment component.

Several data loops existed in the network, where both direct and indirect data informed intervention effectiveness estimates; the possibility of inconsistencies was investigated. Table 3 showed no evidence of substantial discrepancies between the direct and the NMA results; given the uncertainty in the data, only very large differences were likely to result in statistical significance. Results of the consistency and inconsistency models indicated the existence of overall model consistency, as detailed in Appendix I.

Similar analysis was performed on the PSWQ outcome. Results are shown in Appendix J.

### Sensitivity Analysis Results

The sensitivity of the network to specific studies was investigated. In total, 10 analyses with 12 (rather than the total 13) included studies for GAD-7 were performed, and the probability of each intervention being the best was assessed. The SSRI and group CBT continued to have the highest chances of being “best,” with probabilities of around 43 and 30%, respectively.

Two studies (49, 59) had <30 patients. Excluding these studies from the network also removed medication (M) from the comparator set, altering the network structure. As expected, with the reduction in the number of studies informing the network, the uncertainty in the posterior effect distributions increased further. However, no significant changes were observed compared to the main model results.

Similar analysis was performed on the PSWQ outcome. The ranking of active interventions in terms of median PSWQ score decrease vs. no intervention (NI) was unaltered, although higher score decreases were estimated. Comparing the direction and magnitude of differences in median scores at follow-up between GAD-7 and PSWQ results (where available for both), we make three observations (Appendix K). First, the difference in GAD-7 median scores at follow-up between medication and DIs is the largest across all comparators and favors medication. Second, there were no data available for comparisons between DIs and individual therapy, either face-to-face or by telephone, or between DIs and manualized guided self-help (which is the non-digital counterpart of most DIs). Third, the direction of effect favored SDIs for GAD-7 and UDIs for PSWQ.

## Discussion

### Summary and Interpretation

Our systematic review retrieved 21 RCTs of DIs or alternative pathways of care, including no intervention, for GAD. Comparators included in the studies varied. Specifically, interventions or controls could be digital or non-digital and supported or unsupported by clinicians or lay people. The majority of comparisons were between supported digital interventions and no intervention. Using an ANCOVA framework, our main NMA on GAD-7 pooled together post-treatment scores–adjusted for baseline. In addition, the existence of treatment effect modifiers was assessed, several sensitivity analyses were carried out and network consistency evaluated. NMA on the PSWQ outcome was also performed.

Our NMA results suggest that medication is associated with lower anxiety scores at follow-up relative to all other interventions and controls. Medication also ranks first in terms of its likelihood of being most effective, which considers the uncertainty in relative effect estimates. Medication results are based on data from one study. Antidepressant medication as a treatment for GAD is supported by clinical guidelines (75) and previous evidence syntheses. A large NMA (7) of medication against placebo for GAD found that Sertaline (the same antidepressant used in the study by Christensen et al. included in our NMA) improved HAM-A scores by a mean difference of −2·88 (CrI −4·17 to −1·59) from baseline compared to a placebo based on six trials. Another meta-analysis involving a mixed population (13) favored a combined treatment of psychological therapy and medication for all depressive and anxiety disorders, except GAD, where the direction of effect favored antidepressant medication (Venlafaxine) alone.

Previous reviews of DIs that reported GAD-related outcomes (13, 14) used mixed samples of anxiety disorders and depression, without reporting outcomes separate for GAD subgroups. There are no RCTs with GAD populations comparing DIs with non-digital self-help interventions based on a manual rather than a web-based program. Also, no RCTs compare DIs with individual therapy for GAD, either face-to-face or by telephone; the only available comparisons in the literature are between DIs and group therapy.

Due to very wide confidence intervals, our NMA results were inconclusive as to whether DIs for GAD were better than no intervention or non-therapeutic active controls, or whether they confer an additional benefit to standard therapy. Previous meta-analyses have suggested that supported DIs could be as good as face-to-face therapy across depression, anxiety, and somatic disorders (76, 77). However, the mixed samples in these meta-analyses without separate analysis or reporting for GAD sub-samples does not allow any conclusions about the relative efficacy of DIs specific to the treatment and prevention of GAD.

The results for supported vs. unsupported DIs for GAD were counterintuitive, as we would expect supported DIs to rank higher in terms of the likelihood of being “best,” based on a previous meta-analysis in which supported DIs were found to be four times more likely to be effective compared to those without any therapist contact (78). We found that unsupported DIs rank higher than supported DIs in terms of the probability of being best, but vice versa when considering all rankings (SUCRAs). This is consistent with a recent review (79) that reported mixed findings regarding guided vs. unguided DIs and human vs. automated support for DIs. This suggests that the design, content, technology platform or type of reinforcement offered in lieu of personal support in unsupported DIs may be important and account for some of the variability in outcomes.

### Strengths and Limitations

To our knowledge this is the first ANCOVA NMA model to synthesize evidence on two widely used outcomes for GAD. Our NMA makes best use of all currently available RCT-based evidence on DIs for GAD. Despite the sparse and low-quality data, a statistical synthesis can still be useful for decision-makers in mental health (including healthcare professionals, providers and policy-makers, patients and their families, and the research community) who may be considering the use of DIs for GAD, so that they are properly informed about the current status of the evidence base, know which DIs have been shown to be more effective in reducing GAD and prioritize future research.

There was substantial uncertainty around effect estimates of DIs against alternatives for GAD-7. This is driven by the small number of studies informing most comparisons, the small sample sizes used in some of these studies and their high risk of bias across the evidence base, all limiting our confidence in any observed differences in anxiety scores between intervention, comparators, and control arms. These observed differences may simply be due to chance; but in view of the current evidence base we cannot make clear recommendations about the relative effectiveness of DIs against their comparators.

We have to use caution when interpreting the results of our NMA across all different interventions for GAD. Our review has been completed in the context of DIs; it only included RCTs in which at least one of the randomisation arms was a DI. Therefore, we cannot draw any conclusions about the comparative merit of non-digital interventions (psychological or pharmacological) for GAD when these are considered separately to DIs (for example group CBT vs. medication). To be able to do this, we would need to include RCTs in an NMA that would enable 2nd or 3rd order contrasts (e.g., RCTs comparing no intervention and medication), which was beyond the scope of this review. Also, ranking based on likelihood of being best and on SUCRAs does not reflect differences in effectiveness estimates between interventions and controls and credible intervals, that is, we cannot tell whether the differences between ranking position (e.g., between 1st, 2nd, 3rd, 4th, etc.) are clinically meaningful.

Another point of caution, as with all evidence synthesis of complex interventions, is the pooling DIs and their alternatives into groups for analysis based on our classification criteria. Any classification implies interpretation and judgement which is conditional upon the information available from included studies. We note the insufficient reporting of details about “non-therapeutic controls” and waiting list in some studies. Furthermore, we could have split DIs into further categories according to the technology used (e.g., VR, internet, mobile app), or the function of the technology (e.g., adjunct to clinician-delivered therapy vs. patient self-help), or the type of support (e.g., phone calls vs. meetings). This would have created more “nodes” in the NMA models, but also more uncertainty because comparisons between DIs and their alternatives within each subgroup would have been informed by fewer studies.

Many of the included RCTs recruited small samples and involved multiple arms, often comparing different versions of the same intervention, thereby reducing the power of the study. Our evidence synthesis also shows that the majority of RCTs have either a short timeframe for follow-up (up to 12 weeks), or the control group has already crossed to the intervention at the point of a longer follow-up (up to 2 years), which undermines the original randomisation. Consequently, we did not include observations for further follow-up time points, where these were available, nor did we account for time differences in the short-term outcome reporting (post- treatment assessments varied from 3 to 12 weeks). Our NMA results reflect the short-term impact of DIs over an initial treatment period, but there is scant evidence to inform randomized comparisons about effectiveness beyond 12 weeks.

### Recommendations

As GAD is the most prevalent and least studied condition among other common mental health problems, future evidence syntheses will be helpful to focus on GAD populations and stratified GAD subgroups where these are randomized within mixed populations, as means of informing GAD-specific future research and clinical guidelines (75). Feasibility and pilot studies, as well as user involvement in the development of the intervention and delivery protocols, could ensure that the final RCT tests the best possible intervention for GAD. Adaptive designs with improved intervention features and boosted recruitment numbers to a fully powered RCT are preferable to the underpowered studies with multiple arms testing increments of the same DI that we have identified.

Our NMAs and previous literature suggest that antidepressants are an important factor to consider in future studies on DIs for GAD. Psychological interventions–whether digital or non-digital–include participants who are taking medication as part of routine care. It is difficult to disentangle the effects of medication and psychological support for GAD, and future RCTs need to report medication details (name, dose, and duration) and include it as a covariate in their analysis to establish how outcomes with DIs and controls are influenced by concurrent medication use.

The evidence base available in this setting is complex. In particular, the sheer volume of anxiety metrics (45 in total) being reported across the available studies, suggests a lack of consensus on which measures to use in evaluating GAD outcomes. Having a consensus about GAD-specific outcome measures can prevent participant fatigue from completing batteries of different questionnaires and enable comparisons across studies and data synthesis. GAD-7 is more sensitive to changes associated with treatment and therefore may be more suitable for longitudinal clinical research (80). Reporting continuous data on the GAD-7 as a common measure in RCTs with GAD populations will make more studies available for a future statistical synthesis. Including HAM-A in studies of psychological therapies will enable us to compare results with pharmacological studies. Future analyses using multivariate models may be able to make better use of the available evidence by borrowing strength across different outcomes.

Many studies that follow up participants over the longer-term offer the intervention to those randomized to the control group at a crossover point. Many studies that follow up participants over the longer term offer the intervention to those randomized to the control group at a crossover point, potentially biasing any long-term treatment effect (81). Participants are also likely to receive some treatment as part of usual care the longer they remain on waiting lists or non-therapeutic controls, so studies cannot withhold interventions to enable long-term follow-up. As the typical duration of DIs is between 3 and 12 weeks, the follow-up period of future RCTs needs to be longer, for example 6 months, to help us better understand the “stickiness” longer term effects of DIs beyond their initial delivery period. Usual care and waiting lists are poorly reported in RCTs and do not include data on concurrent interventions accessed by participants, including openly available self-help, which can influence the observed difference in outcome between DIs and no intervention. Greater clarity and more detailed reporting about the specific elements of comparators is essential to improve our understanding of the effects of DIs.

## Conclusions

This study is the first to evaluate the effectiveness of DIs specifically in a GAD population. It is also the first to combine all the RCT-based effectiveness evidence from DIs and key comparators in a single modeling framework, allowing the estimation of relative treatment effects for all relevant comparisons. Our results suggest that antidepressant medication is associated with lower anxiety scores at follow-up relative to all other interventions and controls. Results were inconclusive as to whether DIs are better than no intervention and non-therapeutic active controls for GAD, or whether they confer an additional benefit to standard therapy. Overall, our findings are limited in informing decision-making, highlighting how little is currently known about the comparative effectiveness of such interventions. Future primary studies and meta-analyses need to focus on GAD populations rather than mixed samples, or report outcomes specifically for GAD sub-samples if they intend to answer questions about the comparative effectiveness of DIs for GAD. Comparing DIs with manualized (non-digital) self-help and individual therapy, for which there are no current RCTs for GAD populations, will be useful in the context of stepped care. Antidepressant medication for GAD as a first-line treatment against DIs deserves further research and economic modeling. To inform commissioning and potential disinvestment from non-digital alternatives, we need to put the findings of this evidence synthesis into context together with an assessment of the costs of developing and implementing DIs in clinical practice.

## Data Availability Statement

The original contributions presented in the study are included in the article/Supplementary Material, further inquiries can be directed to the corresponding author.

## Author Contributions

PS reviewed the clinical evidence, performed the data synthesis and statistical analysis, interpreted findings, wrote the manuscript, and led the work as a whole. LG reviewed the clinical evidence, classified the interventions, interpreted findings, supported the write up of the manuscript, and all aspects of the review. DM identified and selected the studies, extracted study data, assessed their risk of bias, interpreted findings, and reviewed the manuscript. GN supported the data synthesis and statistical analysis and reviewed the manuscript. DJ contributed to the selection of studies, interpreted findings, and reviewed the manuscript. HM identified and selected the studies, extracted study data, assessed their risk of bias, interpreted findings, and reviewed the manuscript. SD performed the searches and reviewed the manuscript. RC provided support on all aspects of the review and reviewed the manuscript. LB provided support on all aspects of the review and reviewed the manuscript. All authors contributed to the article and approved the submitted version.

## Funding

This work was supported by the UK's National Institute for Health Research (NIHR), Health Technology Assessment (HTA) Programme, Grant Number 17/93/06, 2019.

## Author Disclaimer

This paper presents independent research supported by the National Institute for Health Research (NIHR), Health Technology Assessment (HTA) Programme. The views expressed are those of the authors and not necessarily of the National Health Service, the NIHR, or the Department of Health. The content of this manuscript has previously appeared online as a preprint of the NIHR report: https://eprints.whiterose.ac.uk/171555/.

## Conflict of Interest

LG is the fund holder of the research grant that supported this work. GN is employed by IQVIA. The remaining authors declare that the research was conducted in the absence of any commercial or financial relationships that could be construed as a potential conflict of interest.

## Publisher's Note

All claims expressed in this article are solely those of the authors and do not necessarily represent those of their affiliated organizations, or those of the publisher, the editors and the reviewers. Any product that may be evaluated in this article, or claim that may be made by its manufacturer, is not guaranteed or endorsed by the publisher.
