# Supplementary material for: Digital Interventions for Generalized Anxiety Disorder (GAD): Systematic Review and Network Meta-Analysis
Source: Front Psychiatry. 2021 Dec 6;12:726222. doi: 10.3389/fpsyt.2021.726222 (PMC8685377; doi:10.3389/fpsyt.2021.726222)
Supplement: Supplementary file 1 [file Data_Sheet_1.docx]

**Appendix A – Search Strategies RCTS - Generalized Anxiety Disorder, Mixed Anxiety and Depression – December 2018**

Bibliographic databases

Date of search: 19 December 2018

1. Ovid PsycINFO, n=872

2. Ovid MEDLINE databases, n= 1496

3. Ovid Embase, n=1725

4a. Cochrane Database of Systematic Reviews (CDSR), n=20

4b. Cochrane Central Register of Controlled Trials (CENTRAL) ℅ CRSO, n=1261

5. Web of Science Core Collection, n=1345

6. EBSCO CINAHL, n=791

7. CRD Database (DARE, NHS-EED, HTA), n=73

8 Proquest Dissertations and Theses International, n=73

Total=7656

Duplicates remove=4125

To screen, n=3531

**Ovid PsycINFO** <1806 to December Week 2 2018>

Search Strategy:

--------------------------------------------------------------------------------

1 generalized anxiety disorder/ (2580)

2 GAD.ti,ab,id. (3987)

3 (general* adj3 anxi*).ti,ab,id. (11524)

4 (depress* adj5 anxi*).ti,ab,id,hw. (62980)

5 or/1-4 (71333)

6 digital*.ti,id. (8694)

7 (android or app or apps or avatar* or blog* or CD-ROM or cell phone* or cellphone* or chat room* or computer* or cyber* or (digital* adj (based or deliver* or media* or medium or platform* or technolog*)) or DVD or eHealth or e-health or electronic health or e-mail* or email* or e-Portal or ePortal or ePsych* or e-Psych* or eTherap* or e-therap* or electronic forum* or gaming or information technolog* or instant messag* or messaging or internet* or ipad or i-pad or iphone or i-phone or ipod or i-pod or podcast or smart phone or smartphone or social network* site* or social networking or mHealth or m-health or mobile or multi-media or multimedia or online* or on-line or personal digital assistant or PDA or SMS or social medi* or software or telecomm* or telehealth* or tele-health* or telemed* or tele-med* or telemonitor* or tele-monitor* or telepsych* or tele-psych* or teletherap* or tele-therap* or text messag* or texting or virtual* or web* or WWW).ti,id,hw. (162119)

8 computer mediated communication/ or internet/ or websites/ or blog/ or online community/ or social media/ or online social networks/ (43521)

9 computers/ or computer games/ or digital computers/ or microcomputers/ (18589)

10 mobile devices/ or cellular phones/ or text messaging/ (6472)

11 (eLearning or blended learning).ti,id. (684)

12 (telecomm* or tele-comm*).ti,id. (981)

13 Telemedicine/ or Teleconferencing/ or exp Telecommunications Media/ or exp Audiovisual Communications Media/ (36943)

14 technology/ or information technology/ or exp computer applications/ or computer software/ (108405)

15 (technology based or ((technology or technologies) adj5 (deliver* or wearable or information or communication? or mood or mental or psychiatr*))).ti,id. (9609)

16 computer assisted therapy/ or computer assisted instruction/ or online therapy/ (19071)

17 virtual classrooms/ or virtual teams/ or computer simulation/ or virtual reality/ or computer games/ or simulation games/ (19377)

18 (gaming or gamification or smartwatch* or wearable device? or wearables or videogame or video game or videoconferenc* or video conferenc*).ti,id. (4837)

19 (synchronous or asynchronous or (electronic adj2 deliver*)).ti,id. (1736)

20 artificial intelligen*.ti,ab,id. (4513)

21 artificial intelligence/ or expert systems/ or intelligent tutoring systems/ or machine learning/ or robotics/ (22160)

22 ((computer* or online or internet* or (web adj (based or deliver*)) or digital* or multimedia or multi-media or blended) adj2 (CBT or cognitive or behavio?ral or therap* or psychotherap* or counsel*)).ti,ab,id. (4416)

23 (bCBT or b-CBT or cCBT or c-CBT or iCBT or i-CBT).ti,ab,id. (527)

24 ((distan* or remote*) adj2 (CBT or cogniti* or behavio* or therap* or psychotherap*)).ti,ab,id. (764)

25 (tele* adj2 (cognitive behavi* or CBT)).ti,ab,id. (127)

26 ((computer* or online or internet* or (web adj (based or deliver*)) or digital* or multimedia or multi-media or blended) adj3 (intervention* or program* or bibliotherap* or mindful* or mind training or problem solving or psychoeducat* or psychodrama or rational emotive or RET or reality therap* or role play* or self control or schema* or stress manag* or multicomponent* or multi* component* or (acceptance adj2 commitment))).ti,ab,id. (15944)

27 ((computer* or software or online or internet or (web adj (based or deliver*)) or multimedia) and (psychotherapy or cognitive therapy or behavior therapy or (acceptance and commitment) or bibliotherapy or metacognition or mindfulness or problem solving or psychoeducation or psychodrama or rational emotive or reality therapy or relaxation therapy or role playing or self control)).hw. (5127)

28 ((audio* or CD or CD-ROM or chat room or computer* or cyber* or digital* or DVD or e-mail or email or eHealth* or e-Health* or electronic health or etherap* or e-therap* or internet* or interactive or mobile or multimedia or multi-media or mHealth or online or on-line or podcast or tape or taped or telemed* or telehealth* or telepsych* or teletherap* or tele-therap* or instant messag* or SMS or social medi* or text messag* or texting or instant messag* or software or technolog* or video* or virtual or (web adj (based or deliver*))) adj5 (self-help or (self adj2 (direct* or guid* or unguid* or non-guid* or minim* guidance or minim* contact*)))).ti,ab,id. (1258)

29 ((audio* or CD or CD-ROM or chat room or computer* or cyber* or digital* or DVD or e-mail or email or eHealth* or e-Health* or electronic health or etherap* or e-therap* or internet* or interactive or mobile or multimedia or multi-media or mHealth or online or on-line or podcast or tape or taped or telemed* or telehealth* or telepsych* or teletherap* or tele-therap* or instant messag* or SMS or social medi* or text messag* or texting or instant messag* or software or technolog* or video* or virtual or (web adj (based or deliver*))) adj5 (cognitive behavi* or CBT)).ti,ab,id. (1706)

30 (self-help and (computer* or internet or online or software)).hw. (365)

31 (Beat* the Blues or Blues Away or Blues Begone or blended CBT or bCBT or b-CBT or BluesBegone or BounceBackNow or Bounce Back Now or BrainMaster or Brain Master or BrainGame or Brain Game or BRAVEOnline or BRAVE-online or Brave for Teen* or Brave for Child* or caCCBT or CaptainsLog or Captains Log or CatchIT or Catch-IT or CATTS or Camp Cope-A-Lot or CogMed or Cool Teens or deprexis or FindMe or GETON Mood Enhancer or GET ON Mood Enhancer or Glasgow Steps or GlasgowSteps or GripOpJeDip or Grip Op Je Dip or Help4Mood or Interapy or MasterYourMoodOnline or Master Your Mood or Mindcheck* or MindReading or Mind Reading or MindWise or Mind Wise or MobileType or Mobilyze or MoodGym or Mood Gym or MoodHacker or Mood Hacker or MoodHelper or Mood Helper or MoodMechanic or Mood Mechanic or Moodivate or MyCompass or My Compass or NetCope or Net Cope or OCFighter or OC-Fighter or ODIN or overcoming depression on the internet or PlayAttention or Play Attention or Pratenonline or Praten Online or RoboMemo or SALUDBN or SALUD BN or SmartBrain or Smart Brain or SPARX or StressBusters or Stress Busters or Stresspac or StudentBodies or Student Bodies or The Journey or ThisWayUp or This-Way-Up).ti,ab,id. (14273)

32 (CALM or CAVE or ecompared or e-compared or eSmart* or e-Smart*).ti. (348)

33 (The Journey or Think Feel Do or Bebo or Chatbot or Chat-bot or Club Penguin or Facebook or Franktown or Friendster or Habbo or Jabbersmack or hi5 or iTwixie or MySpace or Orkut or Sweety High or Kidzworld or Tumblr or Twitter or Sina Weibo or Yoursphere or YouTube).ti,id. (6609)

34 or/6-33 (249916)

35 clinical trials.sh. (11168)

36 (randomi#ed or randomi#ation or randomi#ing).ti,ab,id. (76674)

37 (RCT or at random or (random* adj3 (administ* or allocat* or assign* or class* or control* or determine* or divide* or division or distribut* or expose* or fashion or number* or place* or recruit* or split or subsitut* or treat*))).ti,ab,id. (91355)

38 ((single or double or triple or treble) adj2 (blind* or mask* or dummy)).ti,ab,id. (24726)

39 trial.ti. (27029)

40 placebo.ti,ab,id,hw. (38188)

41 treatment outcome.md. (19227)

42 treatment effectiveness evaluation.sh. (22471)

43 mental health program evaluation.sh. (2052)

44 (groups.ab. or study.ti,ab.) and (control* or waitlist* or wait* list* or ((treatment or care) adj2 usual)).ti,ab,id. (356107)

45 or/35-44 (470522)

46 5 and 34 and 45 (956)

47 limit 46 to (english language and yr="1997 -Current") (872)

***************************

**Ovid MEDLINE(R) and Epub Ahead of Print, In-Process & Other Non-Indexed Citations and Daily** <1946 to December 19, 2018>

Search Strategy:

--------------------------------------------------------------------------------

1 ((general* adj3 anxi*) or GAD).ti,ab,kf. (15465)

2 (depress* adj5 anxi*).ti,ab,kf. (68861)

3 ((depression or depressive) and anxiety).hw. (38326)

4 or/1-3 (92921)

5 digital*.ti,kf. (43508)

6 (android or app or apps or avatar* or blog* or CD-ROM or cell phone* or cellphone* or chat room* or computer* or cyber* or (digital* adj (based or deliver* or media* or medium or platform* or technolog*)) or DVD or eHealth or e-health or electronic health or e-mail* or email* or e-Portal or ePortal or ePsych* or e-Psych* or eTherap* or e-therap* or electronic forum* or gaming or information technolog* or instant messag* or messaging or internet* or ipad or i-pad or iphone or i-phone or ipod or i-pod or podcast or smart phone or smartphone or social network* site* or social networking or mHealth or m-health or mobile or multi-media or multimedia or online* or on-line or personal digital assistant or PDA or SMS or social medi* or software or telecomm* or telehealth* or tele-health* or telemed* or tele-med* or telemonitor* or tele-monitor* or telepsych* or tele-psych* or teletherap* or tele-therap* or text messag* or texting or virtual* or web* or WWW).ti,kf,hw. (836145)

7 computer communication networks/ or internet/ or blogging/ or social media/ (83635)

8 electronic mail/ or cell phones/ or smartphone/ or text messaging/ or videoconferencing/ or webcasts as topic/ or wireless technology/ (17784)

9 (eLearning or blended learning).ti,kf. (436)

10 (telecomm* or tele-comm*).ti,kf. (830)

11 Telemedicine/ (18459)

12 (technology based or ((technology or technologies) adj5 (deliver* or wearable or information or communication? or mood or mental or psychiatr*))).ti,kf. (7136)

13 Therapy, Computer-Assisted/ (6390)

14 (gaming or gamification or smartwatch* or wearable device? or wearables or videogame or video game or videoconferenc* or video conferenc*).ti,kf. (3739)

15 (synchronous or asynchronous or (electronic adj2 deliver*)).ti,kf. (10615)

16 artificial intelligen*.ti,ab,kf. (3716)

17 artificial intelligence/ or computer heuristics/ or expert systems/ or knowledge bases/ or machine learning/ or robotics/ (47426)

18 ((computer* or online or internet* or (web adj (based or deliver*)) or digital* or multimedia or multi-media or blended) adj2 (CBT or cognitive or behavio?ral or therap* or psychotherap* or counsel*)).ti,ab,kf. (5012)

19 (bCBT or b-CBT or cCBT or c-CBT or iCBT or i-CBT).ti,ab,kf. (781)

20 ((distance* or remote*) adj2 (CBT or cogniti* or behavio* or therap* or psychotherap*)).ti,ab,kf. (661)

21 ((computer* or online or internet* or (web adj (based or deliver*)) or digital* or multimedia or multi-media or blended) adj3 (intervention* or program* or bibliotherap* or mindful* or mind training or problem solving or psychoeducat* or psychodrama or rational emotive or RET or reality therap* or role play* or self control or schema* or stress manag* or multicomponent* or multi* component* or (acceptance adj2 commitment))).ti,ab,kf. (26916)

22 (tele* adj2 (cognitive behavi* or CBT)).ti,ab,kf. (142)

23 ((computers or computer interface or software or online or internet or (web adj (based or deliver*)) or multimedia) and (psychotherapy or cognitive therapy or behavior therapy or (acceptance and commitment) or bibliotherapy or metacognition or mindfulness or problem solving or psychoeducation or psychodrama or rational emotive or reality therapy or relaxation therapy or role playing or self control)).hw. (3257)

24 ((audio* or CD or CD-ROM or chat room or computer* or cyber* or digital* or DVD or e-mail or email or eHealth* or e-Health* or electronic health or etherap* or e-therap* or internet* or interactive or mobile or multimedia or multi-media or mHealth or online or on-line or podcast or tape or taped or telemed* or telehealth* or telepsych* or teletherap* or tele-therap* or instant messag* or SMS or social medi* or text messag* or texting or instant messag* or software or technolog* or video* or virtual or (web adj (based or deliver*))) adj5 (self-help or (self adj2 (direct* or guid* or unguid* or non-guid* or minim* guidance or minim* contact*)))).ti,ab,kf. (1177)

25 ((audio* or CD or CD-ROM or chat room or computer* or cyber* or digital* or DVD or e-mail or email or eHealth* or e-Health* or electronic health or etherap* or e-therap* or internet* or interactive or mobile or multimedia or multi-media or mHealth or online or on-line or podcast or tape or taped or telemed* or telehealth* or telepsych* or teletherap* or tele-therap* or instant messag* or SMS or social medi* or text messag* or texting or instant messag* or software or technolog* or video* or virtual or (web adj (based or deliver*))) adj5 (cognitive behavi* or CBT)).ti,ab,kf. (1668)

26 (self care and (computers or internet or software)).sh. (1456)

27 (Beat* the Blues or Blues Away or BluesBegone or Blues Begone or blended CBT or bCBT or b-CBT or BounceBackNow or Bounce Back Now or BrainMaster or Brain Master or BrainGame or Brain Game or BRAVEOnline or BRAVE-online or Brave for Teen* or Brave for Child* or caCCBT or CaptainsLog or Captains Log or CatchIT or Catch-IT or CATTS or Camp Cope-A-Lot or CogMed or Cool Teens or deprexis or FindMe or GETON Mood Enhancer or GET ON Mood Enhancer or Glasgow Steps or GlasgowSteps or GripOpJeDip or Grip Op Je Dip or Help4Mood or Interapy or MasterYourMoodOnline or Master Your Mood or Mindcheck* or MindReading or Mind Reading or MindWise or Mind Wise or MobileType or Mobilyze or MoodGym or Mood Gym or MoodHacker or Mood Hacker or MoodHelper or Mood Helper or MoodMechanic or Mood Mechanic or Moodivate or MyCompass or My Compass or NetCope or Net Cope or OCFighter or OC-Fighter or ODIN or overcoming depression on the internet or PlayAttention or Play Attention or Pratenonline or Praten Online or RoboMemo or SALUDBN or SALUD BN or SmartBrain or Smart Brain or SPARX or StressBusters or Stress Busters or Stresspac or StudentBodies or Student Bodies or The Journey or Think Feel Do or ThisWayUp or This-Way-Up).ti,ab,kf. (12185)

28 (CALM or CAVE or ecompared or e-compared or eSmart* or e-Smart*).ti. (2184)

29 (Bebo or Chatbot or Chat-bot or Club Penguin or Facebook or Franktown or Friendster or Habbo or Jabbersmack or hi5 or iTwixie or MySpace or Orkut or Sweety High or Kidzworld or Tumblr or Twitter or Sina Weibo or Yoursphere or YouTube).ti,kf. (2656)

30 or/5-29 (929048)

31 controlled clinical trial.pt. (92795)

32 randomized controlled trial.pt. (472446)

33 (randomi#ed or randomi#ation or randomi#ing).ti,ab,kf. (563847)

34 (RCT or "at random" or (random* adj3 (administ* or allocat* or assign* or class* or cluster or control* or determine* or divide* or division or distribut* or expose* or fashion or number* or place* or pragmatic or quasi or recruit* or split or subsitut* or treat*))).ti,ab,kf. (474903)

35 placebo*.ab,ti,kf. (200959)

36 trial.ab,ti,kf. (529268)

37 ((single or double or triple or treble) adj2 (blind* or mask* or dummy)).ti,ab,kf. (161404)

38 random allocation/ or double-blind method/ or single-blind method/ (260122)

39 (groups.ab. or study.ti,ab.) and (control* or waitlist* or wait* list* or ((treatment or care) adj2 usual)).ti,ab,kf. (1807925)

40 or/31-39 (2658506)

41 4 and 30 and 40 (1521)

42 limit 41 to (english language and yr="1997 -Current") (1496)

***************************

**Ovid Embase** <1974 to 2018 Week 51>

Search Strategy:

--------------------------------------------------------------------------------

1 generalized anxiety disorder/ (9659)

2 (general* adj3 anxi*).ti,ab,kw. (13766)

3 "mixed anxiety and depression"/ (631)

4 (depress* adj5 anxi*).ti,ab,kw. (105033)

5 or/1-4 (116477)

6 digital*.ti,kw. (48641)

7 (android or app or apps or avatar* or blog* or CD-ROM or cell phone* or cellphone* or chat room* or computer* or cyber* or (digital* adj (based or deliver* or media* or medium or platform* or technolog*)) or DVD or eHealth or e-health or electronic health or e-mail* or email* or e-Portal or ePortal or ePsych* or e-Psych* or eTherap* or e-therap* or electronic forum* or gaming or information technolog* or instant messag* or messaging or internet* or ipad or i-pad or iphone or i-phone or ipod or i-pod or podcast or smart phone or smartphone or social network* site* or social networking or mHealth or m-health or mobile or multi-media or multimedia or online* or on-line or personal digital assistant or PDA or SMS or social medi* or software or telecomm* or telehealth* or tele-health* or telemed* or tele-med* or telemonitor* or tele-monitor* or telepsych* or tele-psych* or teletherap* or tele-therap* or text messag* or texting or virtual* or web* or WWW).ti,kw. (304604)

8 internet/ or blogging/ or social media/ (111285)

9 *online system/ (5847)

10 e-mail/ or text messaging/ (21143)

11 mobile application/ or mobile phone/ or smartphone/ (25686)

12 (eLearning or blended learning).ti,kw. (619)

13 telecommunication/ or teleconference/ or wireless communication/ (28266)

14 (telecomm* or tele-comm*).ti,kw. (888)

15 *technology/ or telemedicine/ or telehealth/ or telepsychiatry/ or teletherapy/ (43196)

16 (technology based or ((technology or technologies) adj5 (deliver* or wearable or information or communication? or mood or mental or psychiatr*))).ti,kw. (8942)

17 computer assisted therapy/ or *computer program/ (37911)

18 videoconferencing/ or video game/ or virtual reality/ or webcast/ (19555)

19 (gaming or gamification or smartwatch* or wearable device? or wearables or videogame or video game or videoconferenc* or video conferenc*).ti,kw. (4399)

20 (synchronous or asynchronous or (electronic adj2 deliver*)).ti,kw. (12679)

21 artificial intelligence.ti,ab,kw,hw. (19907)

22 ((computer* or online or internet* or (web adj (based or deliver*)) or digital* or multimedia or multi-media or blended) adj2 (CBT or cognitive or behavio?ral or therap* or psychotherap* or counsel*)).ti,ab,kw. (6575)

23 (bCBT or b-CBT or cCBT or c-CBT or iCBT or i-CBT).ti,ab,kw. (1056)

24 ((distance* or remote*) adj2 (CBT or cogniti* or behavio* or therap* or psychotherap*)).ti,ab,kw. (732)

25 ((computer* or online or internet* or (web adj (based or deliver*)) or digital* or multimedia or multi-media or blended) adj3 (intervention* or program* or bibliotherap* or mindful* or mind training or problem solving or psychoeducat* or psychodrama or rational emotive or RET or reality therap* or role play* or self control or schema* or stress manag* or multicomponent* or multi* component* or (acceptance adj2 commitment))).ti,ab,kw. (33664)

26 ((computer or software or online or internet or (web adj (based or deliver*)) or multimedia) and (psychotherapy or cognitive therapy or behavior therapy or (acceptance and commitment) or bibliotherapy or metacognition or mindfulness or problem solving or psychoeducation or psychodrama or rational emotive or reality therapy or relaxation therapy or role playing or self control)).hw. (8304)

27 ((audio* or CD or CD-ROM or chat room or computer* or cyber* or digital* or DVD or e-mail or email or eHealth* or e-Health* or electronic health or etherap* or e-therap* or internet* or interactive or mobile or multimedia or multi-media or mHealth or online or on-line or podcast or tape or taped or telemed* or telehealth* or telepsych* or teletherap* or tele-therap* or instant messag* or SMS or social medi* or text messag* or texting or instant messag* or software or technolog* or video* or virtual or (web adj (based or deliver*))) adj5 (self-help or (self adj2 (direct* or guid* or unguid* or non-guid* or minim* guidance or minim* contact*)))).ti,ab,kw. (1550)

28 ((audio* or CD or CD-ROM or chat room or computer* or cyber* or digital* or DVD or e-mail or email or eHealth* or e-Health* or electronic health or etherap* or e-therap* or internet* or interactive or mobile or multimedia or multi-media or mHealth or online or on-line or podcast or tape or taped or telemed* or telehealth* or telepsych* or teletherap* or tele-therap* or instant messag* or SMS or social medi* or text messag* or texting or instant messag* or software or technolog* or video* or virtual or (web adj (based or deliver*))) adj5 (cognitive behavi* or CBT)).ti,ab,kw. (2117)

29 (self care and internet).sh. (1805)

30 (The Journey or Think Feel Do or Bebo or Chatbot or Chat-bot or Club Penguin or Facebook or Franktown or Friendster or Habbo or Jabbersmack or hi5 or iTwixie or MySpace or Orkut or Sweety High or Kidzworld or Tumblr or Twitter or Sina Weibo or Yoursphere or YouTube or e-compared or e-smart*).ti,kw. (8787)

31 (CALM or CAVE or ecompared or e-compared or eSmart* or e-Smart*).ti. (2353)

32 (Beat* the Blues or Blues Away or BluesBegone or Blues Begone or blended CBT or bCBT or b-CBT or BounceBackNow or Bounce Back Now or BrainMaster or Brain Master or BrainGame or Brain Game or BRAVEOnline or BRAVE-online or Brave for Teen* or Brave for Child* or caCCBT or CaptainsLog or Captains Log or CatchIT or Catch-IT or CATTS or Camp Cope-A-Lot or CogMed or Cool Teens or deprexis or FindMe or GETON Mood Enhancer or GET ON Mood Enhancer or Glasgow Steps or GlasgowSteps or GripOpJeDip or Grip Op Je Dip or Help4Mood or Interapy or MasterYourMoodOnline or Master Your Mood or Mindcheck* or MindReading or Mind Reading or MindWise or Mind Wise or MobileType or Mobilyze or MoodGym or Mood Gym or MoodHacker or Mood Hacker or MoodHelper or Mood Helper or MoodMechanic or Mood Mechanic or Moodivate or MyCompass or My Compass or NetCope or Net Cope or OCFighter or OC-Fighter or ODIN or overcoming depression on the internet or PlayAttention or Play Attention or Pratenonline or Praten Online or RoboMemo or SALUDBN or SALUD BN or SmartBrain or Smart Brain or SPARX or StressBusters or Stress Busters or Stresspac or StudentBodies or Student Bodies or The Journey or ThisWayUp or This-Way-Up).ti,ab,kw. (16805)

33 or/6-32 (576399)

34 randomized controlled trial/ (527900)

35 randomization.de. (80416)

36 controlled clinical trial/ (459711)

37 *clinical trial/ (17548)

38 placebo.de. (327935)

39 placebo.ti,ab. (280555)

40 trial.ti. (258741)

41 (randomi#ed or randomi#ation or randomi#ing).ti,ab,kw. (805050)

42 (RCT or "at random" or (random* adj3 (administ* or allocat* or assign* or class* or control* or determine* or divide* or division or distribut* or expose* or fashion or number* or place* or recruit* or split or subsitut* or treat*))).ti,ab,kw. (640241)

43 ((singl$ or doubl$ or trebl$ or tripl$) adj3 (blind$ or mask$ or dummy)).mp. (282768)

44 (groups.ab. or study.ti,ab.) and (control* or waitlist* or wait* list* or ((treatment or care) adj2 usual)).ti,ab,kw. (2493291)

45 or/34-44 (3563403)

46 5 and 33 and 45 (1735)

47 limit 46 to yr="1997 -Current" (1725)

***************************

**Cochrane Library:CDSR** (Issue 12 of 2012, 2018)

Search Name: CODI-GAD-Dec-2018

Last Saved: 20/12/2018 15:08:49

Comment:

ID Search

#1 (digital*):ti (Word variations have been searched)

#2 (android or app or apps or avatar* or blog* or CD-ROM or cell phone* or cellphone* or chat room* or computer* or cyber* or (digital* adj (based or deliver* or media* or medium or platform* or technolog*)) or DVD or eHealth or e-health or electronic health or e-mail* or email* or e-Portal or ePortal or ePsych* or e-Psych* or eTherap* or e-therap* or electronic forum* or gaming or (information near technolog*) or (instant next messag*) or messaging or internet* or ipad or i-pad or iphone or i-phone or ipod or i-pod or podcast or (smart next phone) or smartphone or (social next network*next site*) or (social next networking) or mHealth or m-health or mobile or multi-media or multimedia or online* or on-line or (personal next digital next assistant) or PDA or SMS or (social next medi*) or software or telecomm* or telehealth* or tele-health* or telemed* or tele-med* or telemonitor* or tele-monitor* or telepsych* or tele-psych* or teletherap* or tele-therap* or text messag* or texting or virtual* or web* or WWW):ti,kw

#3 MeSH descriptor: [Computer Communication Networks] explode all trees

#4 (internet or blog* or "social media"):ti,kw

#5 MeSH descriptor: [Telecommunications] explode all trees

#6 MeSH descriptor: [Microcomputers] explode all trees

#7 MeSH descriptor: [Computer Communication Networks] explode all trees

#8 ((cell next phone*) or (mobile next phone*) or (mobile next application*) or smartphone* or "text messaging" or e-mail or "electronic mail" or videoconferencing or webcast* or wireless):kw

#9 (eLearning or "blended learning"):ti,kw

#10 (telecomm* or tele-comm* or telemed*):ti,kw

#11 ("technology based" or ((technology or technologies) near (deliver* or wearable or information or communication* or mood* or mental* or psychiatr*))):ti

#12 "Computer Assisted Therapy":kw

#13 MeSH descriptor: [Therapy, Computer-Assisted] this term only

#14 (gaming or gamification or smartwatch* or (wearable next device*) or wearables or videogame or (video next gam*) or videoconferenc* or (video next conferenc*)):ti,kw

#15 (synchronous or asynchronous or (electronic near/3 deliver*)):ti,kw

#16 ((artificial next intelligen*) or "computer heuristics" or (expert next system*) or (knowledge next base*) or "machine learning" or robotics):ti,kw

#17 MeSH descriptor: [Artificial Intelligence] explode all trees

#18 (((computer* or online or internet* or (web next based) or (web next deliver*) or digital* or multimedia or multi-media or blended) near (CBT or cognitive or counsel* or behavioral or behavioural or therap* or psychotherap* or counsel*))):ti,ab,kw

#19 (bCBT or b-CBT or cCBT or c-CBT or iCBT or i-CBT):ti,ab,kw

#20 (((distance* or remote*) near (CBT or cogniti* or counsel* or behavio* or therap* or psychotherap*))):ti,ab,kw

#21 ((computer* or online or internet* or "web based" or (web next deliver*) or digital* or multimedia or multi-media or blended) near (intervention* or program* or bibliotherap* or mindful* or "mind training" or "problem solving" or psychoeducat* or psychodrama or "rational emotive" or RET or (reality next therap*) or (role next play*) or "self control" or schema* or (stress next manag*) or multicomponent* or (multi* next component*) or (acceptance near/2 commitment))):ti,ab,kw

#22 ((audio* or CD or CD-ROM or "chat room" or computer* or cyber* or digital* or DVD or e-mail or email or eHealth* or e-Health* or "electronic health" or etherap* or e-therap* or internet* or interactive or mobile or multimedia or multi-media or mHealth or online or on-line or podcast or tape or taped or telemed* or telehealth* or telepsych* or teletherap* or tele-therap* or (instant next messag*) or SMS or (social next medi*) or (text next messag*) or texting or software or technolog* or video* or virtual or "web based” or (web next deliver*)) near (self-help or (self near/2 (direct* or guid* or unguid* or non-guid* or minim* guidance or minim* contact*)))):ti,ab,kw

#23 ((audio* or CD or CD-ROM or "chat room" or computer* or cyber* or digital* or DVD or e-mail or email or eHealth* or e-Health* or "electronic health" or etherap* or e-therap* or internet* or interactive or mobile or multimedia or multi-media or mHealth or online or on-line or podcast or tape or taped or telemed* or telehealth* or telepsych* or teletherap* or tele-therap* or (instant next messag*) or SMS or (social next medi*) or (text next messag*) or texting or software or technolog* or video* or virtual or "web based" or (web next deliver*)) near ((cognitive next behavi*) or CBT)):ti,ab,kw

#24 ((tele* near/2 (cognitive next behavi*)) or (tele* near/2 CBT)):ti,ab,kw

#25 ("self care" and (computers or internet or software)):kw

#26 ((Beat* near/2 Blues) or "Blues Away" or BluesBegone or "Blues Begone" or "blended CBT" or bCBT or b-CBT or BounceBackNow or "Bounce Back Now" or BrainMaster or "Brain Master" or BrainGame or "Brain Game" or BRAVEOnline or "BRAVE-online" or (Brave near/2 Teen*) or (Brave near/2 Child*) or caCCBT or CaptainsLog or "Captains Log" or CatchIT or Catch-IT or CATTS or "Camp Cope-A-Lot" or CogMed or "Cool Teens" or deprexis or ecompared or e-compared or eSmart* or e-Smart* or FindMe or "GETON Mood Enhancer" or "GET ON Mood Enhancer" or "Glasgow Steps" or GlasgowSteps or GripOpJeDip or "Grip Op Je Dip" or Help4Mood or Interapy or MasterYourMoodOnline or "Master Your Mood" or Mindcheck* or MindReading or "Mind Reading" or MindWise or “Mind Wise” or MobileType or Mobilyze or MoodGym or "Mood Gym" or MoodHacker or "Mood Hacker" or MoodHelper or "Mood Helper" or MoodMechanic or "Mood Mechanic" or Moodivate or MyCompass or "My Compass" or NetCope or "Net Cope" or OCFighter or OC-Fighter or ODIN or (overcoming next depression near/3 internet) or PlayAttention or "Play Attention" or Pratenonline or "Praten Online" or RoboMemo or SALUDBN or "SALUD BN" or SmartBrain or "Smart Brain" or SPARX or StressBusters or "Stress Busters" or Stresspac or StudentBodies or "Student Bodies" or "The Journey" or ThisWayUp or (This next Way next Up) or "Think Feel Do"):ti,ab,kw

#27 (Bebo or Chatbot or Chat-bot or "Club Penguin" or Facebook or Franktown or Friendster or Habbo or Jabbersmack or hi5 or iTwixie or MySpace or Orkut or Sweety High or Kidzworld or Tumblr or Twitter or Sina Weibo or Yoursphere or YouTube):ti,kw

#28 #1 or #2 or #3 or #4 or #5 or #6 or #7 or #8 or #9 or #10 or #11 or #12 or #13 or #14 or #15 or #16 or #17 or #18 or #19 or #20 or #21 or #22 or #23 or #24 or #25 or #26 or #27

#29 ("GENERALIZED ANXIETY DISORDER" OR "GENERALISED ANXIETY DISORDER"):KW

#30 (general* near anxi*):ti,ab

#31 (anxi* near depress*):ti,ab

#32 (anxiety and (depression or depressive)):kw

#33 #29 or #30 or #31 or #32

#34 #28 and #33

CDSR=20

**Cochrane Central Register of Controlled Trials (CENTRAL) ℅ CRSO** (20 December 2018)

#1 digital*:TI 1540

#2 (android or app or apps or avatar* or blog* or CD-ROM or cell phone* or cellphone* or chat room* or computer* or cyber* or (digital* adj (based or deliver* or media* or medium or platform* or technolog*)) or DVD or eHealth or e-health or electronic health or e-mail* or email* or e-Portal or ePortal or ePsych* or e-Psych* or eTherap* or e-therap* or electronic forum* or gaming or (information near technolog*) or (instant next messag*) or messaging or internet* or ipad or i-pad or iphone or i-phone or ipod or i-pod or podcast or (smart next phone) or smartphone or (social next network*next site*) or (social next networking) or mHealth or m-health or mobile or multi-media or multimedia or online* or on-line or (personal next digital next assistant) or PDA or SMS or (social next medi*) or software or telecomm* or telehealth* or tele-health* or telemed* or tele-med* or telemonitor* or tele-monitor* or telepsych* or tele-psych* or teletherap* or tele-therap* or text messag* or texting or virtual* or web* or WWW):ti,kw 19105

#3 MESH DESCRIPTOR Computer Communication Networks EXPLODE ALL TREES 3408

#4 ((internet or blog* or "social media")):TI,AB,KY 7116

#5 MESH DESCRIPTOR Telecommunications EXPLODE ALL TREES 5272

#6 MESH DESCRIPTOR Microcomputers EXPLODE ALL TREES 648

#7 MESH DESCRIPTOR Computer Communication Networks EXPLODE ALL TREES 3408

#8 (((cell next phone*) or (mobile next phone*) or (mobile next application*) or smartphone* or "text messaging" or e-mail or "electronic mail" or videoconferencing or webcast* or wireless)):TI,AB,KY 6353

#9 ((eLearning or "blended learning")):TI,AB,KY 94

#10 ((telecomm* or tele-comm* or telemed*)):TI,AB,KY 2869

#11 (("technology based" or ((technology or technologies) near (deliver* or wearable or information or communication? or mood* or mental* or psychiatr*)))):TI,AB,KY 681

#12 ("Computer Assisted Therapy"):TI,AB,KY 230

#13 MESH DESCRIPTOR Therapy, Computer-Assisted EXPLODE ALL TREES 2834

#14 ((gaming or gamification or smartwatch* or (wearable next device*) or wearables or videogame or (video next gam*) or videoconferenc* or (video next conferenc*))):TI,AB,KY 1987

#15 ((synchronous or asynchronous or (electronic near3 deliver*))):TI,AB,KY 871

#16 (((artificial next intelligen*) or "computer heuristics" or (expert next system*) or (knowledge next base*) or "machine learning" or robotics)):TI,AB,KY 2255

#17 MESH DESCRIPTOR Artificial Intelligence EXPLODE ALL TREES 907

#18 ((((computer* or online or internet* or (web next based) or (web next deliver*) or digital* or multimedia or multi-media or blended) near (CBT or cognitive or counsel* or behavioral or behavioural or therap* or psychotherap* or counsel*)))):TI,AB,KY 5008

#19 ((bCBT or b-CBT or cCBT or c-CBT or iCBT or i-CBT)):TI,AB,KY 461

#20 ((((distance* or remote*) near (CBT or cogniti* or counsel* or behavio* or therap* or psychotherap*)))):TI,AB,KY 308

#21 (((computer* or online or internet* or "web based" or (web next deliver*) or digital* or multimedia or multi-media or blended) near (intervention* or program* or bibliotherap* or mindful* or "mind training" or "problem solving" or psychoeducat* or psychodrama or "rational emotive" or RET or (reality next therap*) or (role next play*) or "self control" or schema* or (stress next manag*) or multicomponent* or (multi* next component*) or (acceptance near2 commitment)))):TI,AB,KY 8769

#22 ((tele* near2 ("cognitive behavi*" or CBT))):TI,AB,KY 157

#23 (((audio* or CD or CD-ROM or "chat room" or computer* or cyber* or digital* or DVD or e-mail or email or eHealth* or e-Health* or "electronic health" or etherap* or e-therap* or internet* or interactive or mobile or multimedia or multi-media or mHealth or online or on-line or podcast or tape or taped or telemed* or telehealth* or telepsych* or teletherap* or tele-therap* or (instant next messag*) or SMS or (social next medi*) or (text next messag*) or texting or software or technolog* or video* or virtual or "web based" or (web next deliver*)) near ((cognitive next behavi*) or CBT))):TI,AB,KY 1274

#24 (((audio* or CD or CD-ROM or "chat room" or computer* or cyber* or digital* or DVD or e-mail or email or eHealth* or e-Health* or "electronic health" or etherap* or e-therap* or internet* or interactive or mobile or multimedia or multi-media or mHealth or online or on-line or podcast or tape or taped or telemed* or telehealth* or telepsych* or teletherap* or tele-therap* or (instant next messag*) or SMS or (social next medi*) or (text next messag*) or texting or software or technolog* or video* or virtual or "web based" or (web next deliver*)) near (self-help or (self near2 (direct* or guid* or unguid* or non-guid* or minim* guidance or minim* contact*))))):TI,AB,KY 529

#25 ("self care" and (computers or internet or software)):ky 578

#26 ((Beat* near2 Blues) or "Blues Away" or BluesBegone or "Blues Begone" or "blended CBT" or bCBT or b-CBT or BounceBackNow or "Bounce Back Now" or BrainMaster or "Brain Master" or BrainGame or "Brain Game" or BRAVEOnline or "BRAVE-online" or (Brave near2 Teen*) or (Brave near2 Child*) or caCCBT or CaptainsLog or "Captains Log" or CatchIT or Catch-IT or CATTS or "Camp Cope-A-Lot" or CogMed or "Cool Teens" or deprexis or ecompared or e-compared or eSmart* or e-Smart* or FindMe or "GETON Mood Enhancer" or "GET ON Mood Enhancer" or "Glasgow Steps" or GlasgowSteps or GripOpJeDip or "Grip Op Je Dip" or Help4Mood or Interapy or MasterYourMoodOnline or "Master Your Mood" or Mindcheck* or MindReading or "Mind Reading" or MindWise or “Mind Wise” or MobileType or Mobilyze or MoodGym or "Mood Gym" or MoodHacker or "Mood Hacker" or MoodHelper or "Mood Helper" or MoodMechanic or "Mood Mechanic" or Moodivate or MyCompass or "My Compass" or NetCope or "Net Cope" or OCFighter or OC-Fighter or ODIN or (overcoming next depression near3 internet) or PlayAttention or "Play Attention" or Pratenonline or "Praten Online" or RoboMemo or SALUDBN or "SALUD BN" or SmartBrain or "Smart Brain" or SPARX or StressBusters or "Stress Busters" or Stresspac or StudentBodies or "Student Bodies" or "The Journey" or ThisWayUp or “This Way Up” or "Think Feel Do") 613

#27 (Bebo or Chatbot or Chat-bot or "Club Penguin" or Facebook or Franktown or Friendster or Habbo or Jabbersmack or hi5 or iTwixie or MySpace or Orkut or "Sweety High" or Kidzworld or Tumblr or Twitter or Sina Weibo or Yoursphere or YouTube) 337

#28 #1 OR #2 OR #3 OR #4 OR #5 OR #6 OR #7 OR #8 OR #9 OR #10 OR #11 OR #12 OR #13 OR #14 OR #15 OR #16 OR #17 OR #18 OR #19 OR #20 OR #21 OR #22 OR #23 OR #24 OR #25 OR #26 OR #27 39701

#29 ("GENERALIZED ANXIETY DISORDER" OR "GENERALISED ANXIETY DISORDER"):ky,kw 527

#30 (general* near anxi*):ti,ab 2177

#31 (anxi* near depress*):ti,ab 7499

#32 (anxiety and (depression or depressive)):kw,ky 8051

#33 #30 OR #31 OR #32 OR #33 13558

#34 #28 AND #33 1261

**Web of Science Core Collection** (20 December 2018)

Indexes=SCI-EXPANDED, SSCI, A&HCI, CPCI-S, CPCI-SSH, ESCI Timespan=All years

# 16 1,345 #15 AND #14

# 15 2,109,319 TOPIC: (randomized or randomization or randomizing or randomised or randomisation or randomising) *OR* TOPIC: (RCT or "at random") *OR* TOPIC: ((random* NEAR (administ* or allocat* or assign* or class* or cluster or control* or determine* or divide* or division or distribut* or expose* or fashion or number* or place* or pragmatic or quasi or recruit* or split or subsitut* or treat*))) *OR* TOPIC: (placebo*) *OR* TOPIC: (trial) *OR* TOPIC: (((single or double or triple or treble) NEAR (blind* or mask* or dummy))) *AND* TOPIC: ((group or groups or study) NEAR (control* or waitlist* or "wait* list*" or ((treatment or care) NEAR usual)))

# 14 2,799 #13 AND #9

# 13 87,922 #12 OR #11 OR #10

# 12 76,206 TOPIC: ((depress* near anxi*))

# 11 20,453 TOPIC: ((general* near anxi*))

# 10 9,201 TOPIC: ("generalized anxiety disorder") *OR* TOPIC: ("generalised anxiety disorder”)

# 9 1,517,820 #8 OR #7 OR #6 OR #5 OR #4 OR #3 OR #2 OR #1

# 8 10,707 TITLE: ((Bebo or Chatbot or Chat-bot or "Club Penguin" or Facebook or Franktown or Friendster or Habbo or Jabbersmack or hi5 or iTwixie or MySpace or Orkut or Sweety High or Kidzworld or Tumblr or Twitter or Sina Weibo or Yoursphere or YouTube))

# 7 14,937 TITLE: (((CALM or CAVE or ecompared or e-compared or eSmart* or e-Smart*)))

# 6 8,911 TOPIC: ((((“Beat* the Blues” or "Blues Away" or BluesBegone or "Blues Begone" or "blended CBT" or bCBT or b-CBT or BounceBackNow or "Bounce Back Now" or BrainMaster or "Brain Master" or BrainGame or "Brain Game" or BRAVEOnline or "BRAVE-online" or “Brave for Teen*” or “Brave for Child*” or caCCBT or CaptainsLog or "Captains Log" or CatchIT or Catch-IT or CATTS or "Camp Cope A Lot" or CogMed or "Cool Teens" or deprexis or ecompared or eSmart* or FindMe or "GETON Mood Enhancer" or "GET ON Mood Enhancer" or "Glasgow Steps" or GlasgowSteps or GripOpJeDip or "Grip Op Je Dip" or Help4Mood or Interapy or MasterYourMoodOnline or "Master Your Mood" or Mindcheck* or MindReading or "Mind Reading" or MindWise or “Mind Wise” or MobileType or Mobilyze or MoodGym or "Mood Gym" or MoodHacker or "Mood Hacker" or MoodHelper or "Mood Helper" or MoodMechanic or "Mood Mechanic" or Moodivate or MyCompass or "My Compass" or NetCope or "Net Cope" or OCFighter or OC-Fighter or ODIN or “overcoming depression on the internet” or PlayAttention or "Play Attention" or Pratenonline or "Praten Online" or RoboMemo or SALUDBN or "SALUD BN" or SmartBrain or "Smart Brain" or SPARX or StressBusters or "Stress Busters" or Stresspac or StudentBodies or "Student Bodies" or "The Journey" or ThisWayUp or “This Way Up” or "Think Feel Do”))))

# 5 2,694 TOPIC: ((((audio* or CD or CD-ROM or "chat room" or computer* or cyber* or digital* or DVD or e-mail or email or eHealth* or e-Health* or "electronic health" or etherap* or e-therap* or internet* or interactive or mobile or multimedia or multi-media or mHealth or online or on-line or podcast or tape or taped or telemed* or telehealth* or telepsych* or teletherap* or tele-therap* or “instant messag*” or SMS or “social medi*” or “text messag*” or texting or software or technolog* or video* or virtual) near (“cognitive behavi*” or CBT))))

# 4 67,553 TOPIC: (((((audio* or CD or CD-ROM or "chat room" or computer* or cyber* or digital* or DVD or e-mail or email or eHealth* or e-Health* or "electronic health" or etherap* or e-therap* or internet* or interactive or mobile or multimedia or multi-media or mHealth or online or on-line or podcast or tape or taped or telemed* or telehealth* or telepsych* or teletherap* or tele-therap* or "instant messag*" or SMS or "social medi*" or "text messag*" or texting or software or technolog* or video* or virtual or "web based" or "web deliver*") near self*))))

# 3 4,303 TOPIC: ((((computer* or online or internet* or "web based" or “web deliver*” or digital* or multimedia or multi-media or blended) near (“acceptance and commitment” or bibliotherap* or mindful* or "mind training" or "problem solving" or psychoeducat* or psychodrama or "rational emotive" or RET or “reality therap*” or “role play*”))))

# 2 39,569 TOPIC: (((computer* or online or internet* or "web based" or "web deliver*" or digital* or multimedia or multi-media or blended) near (CBT or cognitive or counsel* or behavioral or behavioural or psychotherap*))) *OR* TOPIC: ((bCBT or b-CBT or cCBT or c-CBT or iCBT or i-CBT)) *OR* TOPIC: (((distan* or remote*) near (CBT or cogniti* or counsel* or behavio* or psychotherap*)))

# 1 1,413,826 TITLE: ((((android or app or apps or “artificial intelligen*” or asynchronous or avatar* or "blended learning” or blog* or CD-ROM or “cell phone*” or cellphone* or “chat room*” or computer* or cyber* or digital* or DVD or eHealth or e-health “electronic* deliver*” or “deliver* electronically” or “electronic forum*” or “electronic health” or eLearning or e-Learning or e-mail* or email* or e-Portal or ePortal or ePsych* or e-Psych* or eTherap* or e-therap* or “expert system*” or gaming or gamification or “information technolog*” or “information and communication* technolog*” or “instant messag*” or internet* or ipad or i-pad or iphone or i-phone or ipod or i-pod or podcast or “knowledge base*” or “smart phone” or smartphone or “social network* site*” or “social networking” or mHealth or m-health or "machine learning" or mobile or multi-media or multimedia or online* or on-line or “personal digital assistant” or PDA or robotics or SMS or smartwatch* or “smart watch*” “social medi*” or software or synchronous or "technology based” or telecomm* or tele-comm* or telehealth* or tele-health* or telemed* or tele-med* or telemonitor* or tele-monitor* or telepsych* or tele-psych* or teletherap* or tele-therap* or “text messag*” or texting or videoconferencing or “video conferenc*” or virtual* or videogame or “video gam*” or “wearable device*” or wearables or web or webcast* or wireless or WWW))))

**EBSCOHost CINAHL** (20 December 2018)

S33 [Date limired, 1997 onwards] 791

S32 (S20 AND S31) 793

S31 (S21 OR S22 OR S23 OR S24 OR S25 OR S26 OR S27 OR S30) 633,328

S30 (S28 AND S29) 386,551

S29 TI ( (control* or waitlist* or wait* list* or ((treatment or care) N2 usual)) ) OR AB ( (control* or waitlist* or wait* list* or ((treatment or care) N2 usual)) ) 529,162

S28 TI ( group or groups or study ) OR AB ( group or groups or study ) 1,686,442

S27 TI ( ((single or double or triple or treble) N2 (blind* or mask* or dummy)) ) OR AB ( ((single or double or triple or treble) N2 (blind* or mask* or dummy)) ) 37,918

S26 TI trial OR AB trial 252,075

S25 (MH "Placebos") OR TI placebo* OR AB placebo* 52,224

S24 TI ( (RCT or "at random" or (random* N3 (administ* or allocat* or assign* or class* or cluster or control* or determine* or divide* or division or distribut* or expose* or fashion or number* or place* or pragmatic or quasi or recruit* or split or subsitut* or treat*))) ) OR AB ( (RCT or "at random" or (random* N3 (administ* or allocat* or assign* or class* or cluster or control* or determine* or divide* or division or distribut* or expose* or fashion or number* or place* or pragmatic or quasi or recruit* or split or subsitut* or treat*))) ) 192,505

S23 TI ( randomized or randomization or randomizing or randomised or randomisation or randomising ) OR AB ( randomized or randomization or randomizing or randomised or randomisation or randomising ) 194,186

S22 (MM "Clinical Trials”) 16,583

S21 (MH "Randomized Controlled Trials”) 79,164

S20 (S14 AND S19) 1,750

S19 (S15 OR S16 OR S17 OR S18) 37,214

S18 MW ANXIETY AND MW DEPRESS* 20,113

S17 TI (depress* N5 anxi*) OR AB (depress* N5 anxi*) 27,106

S16 TI (general* N3 anxi*) OR AB (general* N3 anxi*) 3,421

S15 (MH "Generalized Anxiety Disorder”) 353

S14 (S1 OR S2 OR S3 OR S4 OR S5 OR S6 OR S7 OR S8 OR S9 OR S10 OR S11 OR S12 OR S13) 267,746

S13 TI ( CALM or CAVE or ecompared or e-compared or eSmart* or e-Smart* ) OR TI ( Bebo or Chatbot or Chat-bot or "Club Penguin" or Facebook or Franktown or Friendster or Habbo or Jabbersmack or hi5 or iTwixie or MySpace or Orkut or "Sweety High" or Kidzworld or Tumblr or Twitter or "Sina Weibo" or Yoursphere or YouTube ) 3,592

S12 TI ( “Beat* the Blues” or "Blues Away" or BluesBegone or "Blues Begone" or "blended CBT" or bCBT or b-CBT or BounceBackNow or "Bounce Back Now" or BrainMaster or "Brain Master" or BrainGame or "Brain Game" or BRAVEOnline or "BRAVE-online" or “Brave for Teen*” or “Brave for Child*” or caCCBT or CaptainsLog or "Captains Log" or CatchIT or Catch-IT or CATTS or "Camp Cope A Lot" or CogMed or "Cool Teens" or deprexis or ecompared or eSmart* or FindMe or "GETON Mood Enhancer" or "GET ON Mood Enhancer" or "Glasgow Steps" or GlasgowSteps or GripOpJeDip or "Grip Op Je Dip" or Help4Mood or Interapy or MasterYourMoodOnline or "Master Your Mood" or Mindcheck* or MindReading or "Mind Reading" or MindWise or “Mind Wise” or MobileType or Mobilyze or MoodGym or "Mood Gym" or MoodHacker or "Mood Hacker" or MoodHelper or "Mood Helper" or MoodMechanic or "Mood Mechanic" or Moodivate or MyCompass or "My Compass" or NetCope or "Net Cope" or OCFighter or OC-Fighter or ODIN or “overcoming depression on the internet” or PlayAttention or "Play Attention" or Pratenonline or "Praten Online" or RoboMemo or SALUDBN or "SALUD BN" or SmartBrain or "Smart Brain" or SPARX or StressBusters or "Stress Busters" or Stresspac or StudentBodies or "Student Bodies" or "The Journey" or ThisWayUp or “This Way Up” or "Think Feel Do” ) OR AB ( “Beat* the Blues” or "Blues Away" or BluesBegone or "Blues Begone" or "blended CBT" or bCBT or b-CBT or BounceBackNow or "Bounce Back Now" or BrainMaster or "Brain Master" or BrainGame or "Brain Game" or BRAVEOnline or "BRAVE-online" or “Brave for Teen*” or “Brave for Child*” or caCCBT or CaptainsLog or "Captains Log" or CatchIT or Catch-IT or CATTS or "Camp Cope A Lot" or CogMed or "Cool Teens" or deprexis or ecompared or eSmart* or FindMe or "GETON Mood Enhancer" or "GET ON Mood Enhancer" or "Glasgow Steps" or GlasgowSteps or GripOpJeDip or "Grip Op Je Dip" or Help4Mood or Interapy or MasterYourMoodOnline or "Master Your Mood" or Mindcheck* or MindReading or "Mind Reading" or MindWise or “Mind Wise” or MobileType or Mobilyze or MoodGym or "Mood Gym" or MoodHacker or "Mood Hacker" or MoodHelper or "Mood Helper" or MoodMechanic or "Mood Mechanic" or Moodivate or MyCompass or "My Compass" or NetCope or "Net Cope" or OCFighter or OC-Fighter or ODIN or “overcoming depression on the internet” or PlayAttention or "Play Attention" or Pratenonline or "Praten Online" or RoboMemo or SALUDBN or "SALUD BN" or SmartBrain or "Smart Brain" or SPARX or StressBusters or "Stress Busters" or Stresspac or StudentBodies or "Student Bodies" or "The Journey" or ThisWayUp or “This Way Up” or "Think Feel Do” ) 9,937

S11 TI ( ((audio* or CD or CD-ROM or “chat room” or computer* or cyber* or digital* or DVD or e-mail or email or eHealth* or e-Health* or “electronic health” or etherap* or e-therap* or internet* or interactive or mobile or multimedia or multi-media or mHealth or online or on-line or podcast or tape or taped or telemed* or telehealth* or telepsych* or teletherap* or tele-therap* or instant messag* or SMS or social medi* or “text messag*” or texting or “instant messag*” or software or technolog* or video* or virtual or “web based” or “web deliver*”) N2 (“cognitive behavi*” or CBT)) ) OR AB ( ((audio* or CD or CD-ROM or “chat room” or computer* or cyber* or digital* or DVD or e-mail or email or eHealth* or e-Health* or “electronic health” or etherap* or e-therap* or internet* or interactive or mobile or multimedia or multi-media or mHealth or online or on-line or podcast or tape or taped or telemed* or telehealth* or telepsych* or teletherap* or tele-therap* or instant messag* or SMS or social medi* or “text messag*” or texting or “instant messag*” or software or technolog* or video* or virtual or “web based” or “web deliver*”) N2 (“cognitive behavi*” or CBT)) ) 781

S10 TI ( ((audio* or CD or CD-ROM or “chat room” or computer* or cyber* or digital* or DVD or e-mail or email or eHealth* or e-Health* or “electronic health” or etherap* or e-therap* or internet* or interactive or mobile or multimedia or multi-media or mHealth or online or on-line or podcast or tape or taped or telemed* or telehealth* or telepsych* or teletherap* or tele-therap* or instant messag* or SMS or social medi* or “text messag*” or texting or “instant messag*” or software or technolog* or video* or virtual or “web based” or “web deliver*”) N2 (self*)) ) OR AB ( ((audio* or CD or CD-ROM or “chat room” or computer* or cyber* or digital* or DVD or e-mail or email or eHealth* or e-Health* or “electronic health” or etherap* or e-therap* or internet* or interactive or mobile or multimedia or multi-media or mHealth or online or on-line or podcast or tape or taped or telemed* or telehealth* or telepsych* or teletherap* or tele-therap* or instant messag* or SMS or social medi* or “text messag*” or texting or “instant messag*” or software or technolog* or video* or virtual or “web based” or “web deliver*”) N2 (self*)) ) 4,420

S9 TI ( (tele* N2 ("cognitive behavi*" or CBT)) ) OR AB ( (tele* N2 ("cognitive behavi*" or CBT)) ) 106

S8 TI ( ((computer* or online or internet* or "web based" or "web deliver*" or digital* or multimedia or multi-media or blended) N2 (intervention* or program* or bibliotherap* or mindful* or "mind training" or "problem solving" or psychoeducat* or psychodrama or "rational emotive" or RET or "reality therap*" or "role play*" or "self control" or schema* or "stress manag*" or multicomponent* or "multi* component*" or "acceptance and commitment")) ) OR AB ( ((computer* or online or internet* or "web based" or "web deliver*" or digital* or multimedia or multi-media or blended) N2 (intervention* or program* or bibliotherap* or mindful* or "mind training" or "problem solving" or psychoeducat* or psychodrama or "rational emotive" or RET or "reality therap*" or "role play*" or "self control" or schema* or "stress manag*" or multicomponent* or "multi* component*" or "acceptance and commitment")) ) 8,332

S7 TI ( ((distance* or remote*) N2 (CBT or cogniti* or behavio* or therap* or psychotherap*)) ) OR AB ( ((distance* or remote*) N2 (CBT or cogniti* or behavio* or therap* or psychotherap*)) ) 217

S6 TI ( (bCBT or b-CBT or cCBT or c-CBT or iCBT or i-CBT) ) OR AB ( (bCBT or b-CBT or cCBT or c-CBT or iCBT or i-CBT) ) 274

S5 TI ( ((computer* or online or internet* or "web based" or "web deliver*" or digital* or multimedia or multi-media or blended) N2 (CBT or cognitive or behavio* or therap* or psychotherap* or counsel*)) ) OR AB ( ((computer* or online or internet* or "web based" or "web deliver*" or digital* or multimedia or multi-media or blended) N2 (CBT or cognitive or behavio* or therap* or psychotherap* or counsel*) ) 3,827

S4 TI ( (“artificial intelligen*” or eLearning or “blended learning” or “technology based”) ) OR TI ( (gaming or gamification or smartwatch* or “wearable device*” or wearables or videogame or “video game” or videoconferenc* or “video conferenc*”) ) 2,888

S3 (MH "Cellular Phone”) OR (MH "Computers, Hand-Held") OR (MH "Computers, Portable") OR MH (“Electronic Bulletin Boards”) OR (MH "Electronic Mail”) OR (MH "Instant Messaging") OR (MH “Internet") OR (MH “Microcomputers”) OR (MH “Smartphone") OR (MH "Social Media") OR (MH "Social Networking") OR MH "Telecommunications") OR (MH "Teleconferencing") OR (MH "Telehealth") OR (MH "Telemedicine") OR (MH "Telenursing") OR MH (“Telephone") OR (MH “Telepsychiatry") OR (MH "Text Messaging") OR (MH “Videoconferencing") OR (MH "Wireless Communications”) OR (MH "World Wide Web”) OR (MH “Webcasts”)) 161,003

S2 (MH "Therapy, Computer Assisted”) 4,948

S1 TI (android or app or apps or “artificial intelligen*” or asynchronous or avatar* or "blended learning” or blog* or CD-ROM or “cell phone*” or cellphone* or “chat room*” or computer* or cyber* or digital* or DVD or eHealth or e-health “electronic* deliver*” or “deliver* electronically” or “electronic forum*” or “electronic health” or eLearning or e-Learning or e-mail* or email* or e-Portal or ePortal or ePsych* or e-Psych* or eTherap* or e-therap* or “expert system*” or gaming or gamification or “information technolog*” or “information and communication* technolog*” or “instant messag*” or internet* or ipad or i-pad or iphone or i-phone or ipod or i-pod or podcast or “knowledge base*” or “smart phone” or smartphone or “social network* site*” or “social networking” or mHealth or m-health or "machine learning" or mobile or multi-media or multimedia or online* or on-line or “personal digital assistant” or PDA or robotics or SMS or smartwatch* or “smart watch*” “social medi*” or software or synchronous or "technology based” or telecomm* or tele-comm* or telehealth* or tele-health* or telemed* or tele-med* or telemonitor* or tele-monitor* or telepsych* or tele-psych* or teletherap* or tele-therap* or “text messag*” or texting or videoconferencing or “video conferenc*” or virtual* or videogame or “video gam*” or “wearable device*” or wearables or web or webcast* or wireless or WWW) 134,618

**CRD Databases** (DARE, HTA, NHS-EED (Archived database, from searches to Dec 2014)

1 (digital*):TI 129

2 ((android or app or apps or avatar* or blog* or CD-ROM or cell phone* or cellphone* or chat room* or computer* or cyber* or DVD or eHealth or e-health or electronic health or e-mail* or email* or e-Portal or ePortal or ePsych* or e-Psych* or eTherap* or e-therap* or electronic forum* or gaming or information technolog* or instant messag* or messaging or internet* or ipad or i-pad or iphone or i-phone or ipod or i-pod or podcast or smart phone or smartphone or social network* site* or social networking or mHealth or m-health or mobile or multi-media or multimedia or online* or on-line or personal digital assistant or PDA or SMS or social medi* or software or telecomm* or telehealth* or tele-health* or telemed* or tele-med* or telemonitor* or tele-monitor* or telepsych* or tele-psych* or teletherap* or tele-therap* or text messag* or texting or virtual* or web* or WWW)):TI 1007

3 MeSH DESCRIPTOR internet EXPLODE ALL TREES 259

4 MeSH DESCRIPTOR Telecommunications EXPLODE ALL TREES 664

5 MeSH DESCRIPTOR Therapy, Computer-Assisted 111

6 (((eLearning or blended learning))) 1

7 (((telecomm* or tele-comm*))) 49

8 ((((technology based or ((technology or technologies) near (deliver* or wearable or information or communication? or mood* or mental* or psychiatr*)))))) 200

9 ((((gaming or gamification or smartwatch* or wearable device* or wearables or videogame or video game or videoconferenc* or video conferenc*)))) 73

10 ((((synchronous or asynchronous or electronic* deliver* or deliver* electronic*)))) 78

11 (( (artificial intelligen*))) 6

12 ((((bCBT or b-CBT or cCBT or c-CBT or iCBT or i-CBT)))) 19

13 (((((distan* or remote*) NEAR (CBT or cogniti* or behavio* or therap* or psychotherap* or counsel*))))) 28

14 ((((((computer* or online or internet* or web based or web deliver* or digital* or multimedia or multi-media or blended) NEAR (CBT or cognitive or behavio* or therap* or psychotherap* or counsel*)))))) 289

15 ((( ((computer* or online or internet* or web based or web deliver* or digital* or multimedia or multi-media or blended) NEAR (intervention* or program* or bibliotherap* or mindful* or mind training or problem solving or psychoeducat* or psychodrama or rational emotive or RET or reality therap* or role play* or self control or schema* or stress manag* or multicomponent* or multi* component* or (acceptance NEAR2 commitment)))) )) 437

16 (( ((tele* NEAR (cognitive behavi* or CBT))))) 9

17 (((((audio* or CD or CD-ROM or chat room or computer* or cyber* or digital* or DVD or e-mail or email or eHealth* or e-Health* or electronic health or etherap* or e-therap* or internet* or interactive or mobile or multimedia or multi-media or mHealth or online or on-line or podcast or tape or taped or telemed* or telehealth* or telepsych* or teletherap* or tele-therap* or instant messag* or SMS or social medi* or text messag* or texting or instant messag* or software or technolog* or video* or virtual or web based or web deliver*) near self*)))) 109

18 (((CALM or CAVE or ecompared or e-compared or eSmart* or e-Smart*))) 41

19 (((Bebo or Chatbot or Chat-bot or Club Penguin or Facebook or Franktown or Friendster or Habbo or Jabbersmack or hi5 or iTwixie or MySpace or Orkut or Sweety High or Kidzworld or Tumblr or Twitter or Sina Weibo or Yoursphere or YouTube) )) 4

20 ((Beat* the Blues or Blues Away or BluesBegone or Blues Begone or blended CBT or bCBT or b-CBT or BounceBackNow or Bounce Back Now or BrainMaster or Brain Master or BrainGame or Brain Game or BRAVEOnline or BRAVE-online or Brave for Teen* or Brave for Child* or caCCBT or CaptainsLog or Captains Log or CatchIT or Catch-IT or CATTS or Camp Cope-A-Lot or CogMed or Cool Teens or deprexis or FindMe or GETON Mood Enhancer or GET ON Mood Enhancer or Glasgow Steps or GlasgowSteps or GripOpJeDip or Grip Op Je Dip or Help4Mood or Interapy or MasterYourMoodOnline or Master Your Mood or Mindcheck* or MindReading or Mind Reading or MindWise or Mind Wise or MobileType or Mobilyze or MoodGym or Mood Gym or MoodHacker or Mood Hacker or MoodHelper or Mood Helper or MoodMechanic or Mood Mechanic or Moodivate or MyCompass or My Compass or NetCope or Net Cope or OCFighter or OC-Fighter or ODIN or overcoming depression on the internet or PlayAttention or Play Attention or Pratenonline or Praten Online or RoboMemo or SALUDBN or SALUD BN or SmartBrain or Smart Brain or SPARX or StressBusters or Stress Busters or Stresspac or StudentBodies or Student Bodies or The Journey or Think Feel Do or ThisWayUp or This-Way-Up)) 15

21 (((((audio* or CD or CD-ROM or chat room or computer* or cyber* or digital* or DVD or e-mail or email or eHealth* or e-Health* or electronic health or etherap* or e-therap* or internet* or interactive or mobile or multimedia or multi-media or mHealth or online or on-line or podcast or tape or taped or telemed* or telehealth* or telepsych* or teletherap* or tele-therap* or instant messag* or SMS or social medi* or text messag* or texting or instant messag* or software or technolog* or video* or virtual or web based or web deliver*) near (cognitive behavi* or CBT))))) 80

22 (#1 OR #2 OR #3 OR #4 OR #5 OR #6 OR #7 OR #8 OR #9 OR #10 OR #11 OR #12 OR #13 OR #14 OR #15 OR #16 OR #17 OR #18 OR #19 OR #20 OR #21) 2180

23 ("generalised anxiety disorder") OR ("generalized anxiety disorder”) 94

24 ((general* near anxi*)) 152

25 ((depress* near anxi*)) 558

26 MeSH DESCRIPTOR anxiety 308

27 MeSH DESCRIPTOR depression 639

28 MeSH DESCRIPTOR depressive disorder EXPLODE ALL TREES 1030

29 (#26 AND #27) 151

30 (#26 AND #28) 21

31 (#23 OR #24 OR #25 OR #29 OR #30) 755

32 (#22 AND #31) 73

**Proquest Dissertations and Theses** (20 December 2018)

S10 S8 AND S9 (73)

S9 ti(android OR app OR apps OR avatar* OR blog* OR CD-ROM OR cell phone* OR cellphone* OR chat room* OR computer* OR cyber* OR digital* OR DVD OR eHealth OR e-health OR electronic health OR e-mail* OR email* OR e-Portal OR ePortal OR ePsych* OR e-Psych* OR eTherap* OR e-therap* OR electronic forum* OR gaming OR information technolog* OR instant messag* OR messaging OR internet* OR ipad OR i-pad OR iphone OR i-phone OR ipod OR i-pod OR podcast OR smart phone OR smartphone OR social network* site* OR social networking OR mHealth OR m-health OR mobile OR multi-media OR multimedia OR online* OR on-line OR personal digital assistant OR PDA OR SMS OR social medi* OR software OR telecomm* OR telehealth* OR tele-health* OR telemed* OR tele-med* OR telemonitor* OR tele-monitor* OR telepsych* OR tele-psych* OR teletherap* OR tele-therap* OR text messag* OR texting OR virtual* OR web* OR WWW) (121,843)

S8 S6 AND S7 (2,217)

S7 S4 OR S5 (229,605)

S6 S1 OR S2 OR S3 (12,414)

S5 noft((control NEAR group*)) (53,298)

S4 noft((RCT or random* or placebo* or trial)) (193,160)

S3 noft((depress* NEAR anxi*)) (11,024)

S2 noft((general* NEAR anxi*)) (1,979)

S1 noft("generalized anxiety" or "generalised anxiety") (742)
