# Supplementary material for: Digital Interventions for Generalized Anxiety Disorder (GAD): Systematic Review and Network Meta-Analysis
Source: Front Psychiatry. 2021 Dec 6;12:726222. doi: 10.3389/fpsyt.2021.726222 (PMC8685377; doi:10.3389/fpsyt.2021.726222)
Supplement: Supplementary file 3 [file Data_Sheet_3.docx]

**Appendix C Methods for statistical analysis and synthesis model**

**C1. Statistical synthesis model**

Using the random-effects approach, the NMA ANCOVA model used takes the following form:

$${y1}_{i,k} \sim N \left( \theta_{i,k} , {\sigma1}_{i,k}^{2} \right)$$

$$\theta_{i,k}= \mu_{i}+ \delta_{i,bk}+ \beta_{bk}* {y0}_{i,k}$$

$$\delta_{i,bk} \sim N \left( d_{bk}, \tau^{2} \right)$$

$$d_{bk}= d_{Ak}- d_{Ab} , d_{AA}=0$$

$\beta_{bk}= \beta_{Ak}- \beta_{Ab} , \beta_{AA}=0$ (1)

The set of treatments included in these trials are labelled [A,B,C,…], where A is the reference treatment and ${y1}_{i,k}$ and ${\sigma1}_{i,k}^{2}$ are the study *I* and arm *k*-specific post-treatment measurement (the assessment closest to 2 months) and their associated standard errors. $\theta_{i,k}$ is the linear predictor that uses the identity link function, with $\mu_{i}$ being the study-specific baseline, parameters for the reference treatment $b$ in each study (which is not necessarily the reference treatment of the network i.e. treatment A), $\delta_{i, bk}$ the study-specific relative treatment effects between the treatment included in arm $k$ and the treatment included in the baseline arm $b$ of study $i$. $\beta_{bk}$ represent the treatment-specific coefficients that adjust for the pre-treatment (i.e. baseline) measurements ${y0}_{i,k}$ under the ANCOVA model. $\delta_{i,kl}$ are assumed to follow a random-effect approach with mean $d_{bk}$ and a between-studies heterogeneity $\tau^{2}$ that is assumed to be common across all treatment comparisons to assist identification. For trials that use an active control treatment (i.e. $b\neq A$) the consistency assumption is imposed in the form of a set of functional relationships amongst basic parameters (e.g. $d_{Ak}$). Note that $\beta_{AA}$ is assumed to be zero indicating that patients who did not receive any treatment are expected to neither improve nor worsen during the duration of treatment (i.e. null placebo effect). Finally, we assume that the effect of the baseline measurement is common across all treatments so that $\beta_{Ak}=\beta$, implying that when two active treatments are compared in a trial, the baseline effects are offset. Vague prior distributions were assigned to all parameters i.e. $d_{Ak}, \beta\sim N \left( 0, {10}^{-6} \right)$and $\tau\sim Unif\left( 0,10 \right)$.

Meta-regression is the most commonly employed method to explore the influence of particular study-level covariates on the relative effect. A range of approaches can be used to model comparison-specific treatment-effect interactions.(2) In this analysis we assumed a common effect interaction (i.e. a single interaction term is assumed to apply to all comparisons with no intervention) as this was deemed more clinically plausible and also less data demanding. However, this method requires that all studies report data on the covariate(s) in question. For the trials informing the NMA, complete data for disease severity (as a binary covariate mild to moderate/moderate to severe) was obtained, but not for the other two potential effect modifiers. Under these circumstances, one option is to exclude studies for which data on the covariate is missing and perform a meta-regression on the subset of studies that provide covariate information; however, this approach may lead to a smaller network (with less interventions being compared) and ‘weaker’ (with less evidence informing it). Alternatively, to preserve all studies (and treatments), we may assume that the covariate is distributed across studies according to a Beta distribution, the hyperparameters of which are assigned non-informative priors and are estimated within the model through the MCMC simulation in order to impute missing covariate information (multiple imputation procedure assuming ‘missingness’ mechanism of ‘missing at random’). The meta-regression model extends the aforementioned NMA ANCOVA model so that the linear predictor is now:

$$\theta_{i,k}= \mu_{i}+ \delta_{i,bk}+ \beta_{bk}* {y0}_{i,k}+ +B_{bk}*X_{i}$$

$$B_{bk}= B_{Ak}- B_{Ab} , B_{AA}=0$$

$X_{i} \sim Beta (a,b)$ (2)

$B_{Ak}$ are again assumed independent of treatment comparison so that $B_{Ak}=B$ which represents the additional effect that is observed not due to the treatment, but due to the interaction of the treatment with the study-level covariate. When $X_{i}$ represent the study-level covariate values, and are assigned a Beta distribution with hyperparameters $a,b$ which are estimated within the model and assigned vague priors $a,b \sim Unif(0,1000)$. Since $X_{i}$ are proportions, a Beta distribution is perhaps the most reasonable distributional assumption. The effect-modification for the reference treatment is also assumed to be zero.

**C2. WinBUGS code for main synthesis model**

The WinBUGS modelling code is provided below followed by a summary table of all variables included in the dataset and R-code describing the specification of initial values for two chains.

**WinBUGS model code**

model {

for(i in 1:NS) {

w[i,1]<- 0

delta[i,1]<- 0

mu[i] ~ dnorm(0,1.0E-6)

for (k in 1:na[i]) {

y1[i,k] ~ dnorm(theta[i,k], prec[i,k]) #likelihood function

theta[i,k] <- mu[i] + delta[i,k]

var[i,k] <- pow(se1[i,k], 2)

prec[i,k] <- 1/var[i,k]

dev[i,k] <- (y1[i,k] - theta[i,k]) * (y1[i,k] - theta[i,k]) * prec[i,k] #residual deviance }

resdev[i] <- sum(dev[i,1:na[i]])

for (k in 2:na[i]) {

#consistency model for treatment effects and baseline adjustment

delta[i,k] ~ dnorm(md[i,k],precd[i,k])

md[i,k]<- d[t[i,k]] - d[t[i,1]] + (b_base[t[i,k]] - b_base[t[i,1]]) * y0[i,k] + sw[i,k]

precd[i,k] <- pre * 2 * (k - 1)/k

#correction for multi-arm trials

w[i,k]<- delta[i,k] - d[t[i,k]] + d[t[i,1]]

sw[i,k]<- sum(w[i,1:k-1]) / (k - 1)

}

}

#total Residual Deviance

totresdev <- sum(resdev[])

d[1]<-0

for (k in 2:NT) {

#prior on treatment effects and baseline score effects

d[k] ~ dnorm(0,1.0E-6)

b_base[k] <- b_basey

}

#prior on random treatment effect variance

tau ~ dunif(0,10)

tau.sq<- tau * tau

pre<- 1 / (tau.sq)

#prior on impact of baseline score on final outcome score

b_basey ~ dnorm(0,1.0E-6)

b_base[1]<-0

# pairwise effects

for (c in 1:(NT - 1)) {

for (k in (c + 1):NT) {

ef[c,k] <- d[k] - d[c]

}

}

# Treatment A baseline, based on average of the trials including No intervention

for (i in 1:NS) {

mu1[i] <- mu[i] * equals(t[i,1],1)

}

mn.mu1<- sum(mu1[]) / 6

#Posterior distributions of absolute post-treatment scores

for (k in 1:NT) {

T[k]<- mn.mu1 + d[k] + b_base[k]*mn.mu1

}

# ranking and prob{treatment k is the best}

for (k in 1:NT) {

rk[k]<- NT + 1 - rank(T[],k)

best[k]<- equals(rk[k],7)

}

}

**C3. Description of datasets and variables**

| Object | Variable | Description |
| --- | --- | --- |
| Data set descriptors / constants | na | Number of arms in studies in dataset |
|  | NS | Number of trials in dataset |
|  | NT | Number of treatments in dataset |
| Data | y1 | Data on final outcome mean score (arm-level) |
|  | y0 | Data on baseline outcome mean score (arm-level) |
|  | t | Treatment code |
|  | se1 | Data on final outcome standard error of mean score (arm-level) |

**R-code used to generate initial values (only one set shown for didactic purposes)**

list(d = c(NA,0,0,0,0,0,0), mu = c(0,0,0,0,0, 0,0,0,0,0,0,0,0), b_basey = c(0), tau = c(1))
