# Supplementary material for: Digital Interventions for Generalized Anxiety Disorder (GAD): Systematic Review and Network Meta-Analysis
Source: Front Psychiatry. 2021 Dec 6;12:726222. doi: 10.3389/fpsyt.2021.726222 (PMC8685377; doi:10.3389/fpsyt.2021.726222)
Supplement: Supplementary file 4 [file Data_Sheet_4.docx]

**Appendix D Excluded studies by reason**

**Protocols (116)**

1. isrctn15890967. Differences in long-term outcome: face to face v internet enabled cognitive behavioural therapy (CBT). Http://wwwwhoint/trialsearch/trial2aspx? Trialid=isrctn15890967. 2016.

2. isrctn71327173. A pilot research project into the use of online resources for low mood and anxiety in the voluntary sector. Http://wwwwhoint/trialsearch/trial2aspx? Trialid=isrctn71327173. 2007.

3. isrctn79652741. A pilot randomised controlled trial of an internet-based cognitive behavioral therapy (CBT) treatment for adolescent anxiety. Http://wwwwhoint/trialsearch/trial2aspx? Trialid=isrctn79652741. 2012.

4. Andersson G. Psychodynamic Internet Treatment Versus Cognitive Behavioral Therapy (CBT) for Generalized Anxiety Disorder (NCT01312116). Http://clinicaltrialsgov/ct2/show/NCT01312116. 2011.

5. Christensen H, Griffiths KM, Mackinnon AJ, Kalia K, Batterham PJ, Kenardy J, et al. Protocol for a randomised controlled trial investigating the effectiveness of an online e health application for the prevention of Generalised Anxiety Disorder. BMC Psychiatry. 2010;10:25.

6. Christensen H, Guastella AJ, Mackinnon AJ, Griffiths KM, Eagleson C, Batterham PJ, et al. Protocol for a randomised controlled trial investigating the effectiveness of an online e-health application compared to attention placebo or sertraline in the treatment of generalised anxiety disorder. Trials. 2010;11:48.

7. Nct. Internet-based Treatment of Generalized Anxiety Disorder. https://clinicaltrialsgov/show/NCT01570374. 2012.

8. Johansson R, Hesser H, Ljotsson B, Frederick RJ, Andersson G. Transdiagnostic, affect-focused, psychodynamic, guided self-help for depression and anxiety through the internet: Study protocol for a randomised controlled trial. BMJ Open. 2012;2 (6) (no pagination)(e002167).

9. Paxling B. A randomized clinical trial of internet-delivered cognitive behaviour therapy for generalized anxiety disorder [UMIN000001353]. WHO International Clinical Trials Registry [wwwwhoint/ictrp]. 2008.

10. Richards D, Timulak L, Doherty G, Sharry J, McLoughlin O, Rashleigh C, et al. Low-intensity internet-delivered treatment for generalized anxiety symptoms in routine care: protocol for a randomized controlled trial. Trials. 2014;15:145.

11. ntr4993. Preventing childhood anxiety disorders: is a video game as effective as a CBT-based program? Http://wwwwhoint/trialsearch/trial2aspx? Trialid=ntr4993. 2015.

12. NTR4477. A randomized controlled trail to evaluate the effectiveness of a videogame intervention (Dojo) for anxious-aggressive adolescents. http://wwwwhoint/trialsearch/Trial2aspx?TrialID=NTR4477. 2014.

13. NTR4379. A randomized controlled trial to test the effectiveness of an immersive 3D video game in preventing anxiety. http://wwwwhoint/trialsearch/Trial2aspx?TrialID=NTR4379. 2014.

14. chictr-ior-15006470. Internet-based mindfulness and rumination-focused cognitive behavioural therapy as selective prevention of anxiety and depression: a randomised controlled trial. Http://wwwwhoint/trialsearch/trial2aspx? Trialid=chictr-ior-15006470. 2015.

15. actrn12610001058066. A randomized controlled trial of the effects of different levels of graded support on symptoms of anxiety and depression in adults using the Internet-based Wellbeing Course. Http://wwwwhoint/trialsearch/trial2aspx? Trialid=actrn12610001058066. 2010.

16. actrn12611000508976. A randomised controlled trial of the Cool Teens computerised program for anxious adolescents compared to wait list. Http://wwwwhoint/trialsearch/trial2aspx? Trialid=actrn12611000508976. 2011.

17. actrn12611001055998. The Wellbeing Program for Anxiety and Depression - A Randomised Controlled Trial. Http://wwwwhoint/trialsearch/trial2aspx? Trialid=actrn12611001055998. 2011.

18. actrn12612000212853. A randomised controlled trial (RCT) of brief internet-delivered education about managing symptoms of anxiety and depression in tertiary students. Http://wwwwhoint/trialsearch/trial2aspx? Trialid=actrn12612000212853. 2012.

19. actrn12612000214831. A randomised controlled trial (RCT) of brief internet-delivered education about managing symptoms of anxiety and depression in tertiary Chinese students. Http://wwwwhoint/trialsearch/trial2aspx? Trialid=actrn12612000214831. 2012.

20. actrn12612000351819. An Evaluation of Stepped Care for Child Anxiety. Http://wwwwhoint/trialsearch/trial2aspx? Trialid=actrn12612000351819. 2012.

21. actrn12612000414819. The Clinical Research Unit for Anxiety and Depression (CRUfAD) Schools Program for the Prevention of Adolescent Anxiety and Depression - A Randomised Controlled Trial. Http://wwwwhoint/trialsearch/trial2aspx? Trialid=actrn12612000414819. 2012.

22. actrn12612000421831. Is internet-delivered treatment specifically for symptoms of depression as effective as internet-delivered treatment for symptoms of both depression and anxiety, and are self-guided versions of these as effective as therapist-guided versions? Http://wwwwhoint/trialsearch/trial2aspx? Trialid=actrn12612000421831. 2012.

23. actrn12612000431820. Is internet-delivered treatment specifically for symptoms of panic disorder as effective as internet-delivered treatment for symptoms of both anxiety and depression, and are self-guided versions of these as effective as therapist-guided versions? Http://wwwwhoint/trialsearch/trial2aspx? Trialid=actrn12612000431820. 2012.

24. actrn12612000433808. Is internet-delivered treatment specifically for symptoms of generalised anxiety disorder as effective as internet-delivered treatment for symptoms of both anxiety and depression, and are self-guided versions of these as effective as therapist-guided versions? Http://wwwwhoint/trialsearch/trial2aspx? Trialid=actrn12612000433808. 2012.

25. actrn12613000502730. A randomised controlled trial comparing Internet based cognitive behavioural therapy (CBT) versus a cognitive behavioural therapy (CBT) self help book versus a meditation self help book versus a waitlist control condition for adults with depression on severity of symptoms and depression. Http://wwwwhoint/trialsearch/trial2aspx? Trialid=actrn12613000502730. 2013.

26. actrn12613001083785. Randomised controlled trial of the effectiveness of Mental Health Guru, an internet-based workplace induction program for depression and anxiety. Http://wwwwhoint/trialsearch/trial2aspx? Trialid=actrn12613001083785. 2013.

27. actrn12614001318673. A randomised controlled trial and consumer characteristics of the Mindful eHealth wellbeing program. Http://wwwwhoint/trialsearch/trial2aspx? Trialid=actrn12614001318673. 2014.

28. actrn12614001319662. A randomised controlled trial of the efficacy of the Panic eHealth wellbeing program. Http://wwwwhoint/trialsearch/trial2aspx? Trialid=actrn12614001319662. 2014.

29. actrn12615000112561. Behavioural and Cognitive Changes During Self-Guided Internet-Delivered Cognitive-Behavioural Therapy for Anxiety and Depression: a Randomised Controlled Trial. Http://wwwwhoint/trialsearch/trial2aspx? Trialid=actrn12615000112561. 2015.

30. actrn12615000217505. Randomised controlled trial of Cool Little Kids Online: a parenting program to increase young children’s confidence and prevent anxiety problems. Http://wwwwhoint/trialsearch/trial2aspx? Trialid=actrn12615000217505. 2015.

31. actrn12615000473561. A randomised controlled trial evaluating the effectiveness of a one module eHealth wellbeing program (THRIVE) on increasing wellbeing and decreasing stress. Http://wwwwhoint/trialsearch/trial2aspx? Trialid=actrn12615000473561. 2015.

32. actrn12615000927527. A randomised controlled trial comparing internet-delivered Cognitive Behavioural Therapy (iCBT), internet-delivered Mindfulness-Enhanced Cognitive Behavioural Therapy, internet-delivered Mindfulness Skills Training versus usual care for adults with depression and/or anxiety. Http://wwwwhoint/trialsearch/trial2aspx? Trialid=actrn12615000927527. 2015.

33. actrn12615001015538. A randomised controlled trial and consumer characteristics of iChooseWell eHealth: a comprehensive biopsychosocial online wellbeing program to decrease stress. Http://wwwwhoint/trialsearch/trial2aspx? Trialid=actrn12615001015538. 2015.

34. actrn12615001211550. Can a tailored online program reduce mental health symptoms in adults? Http://wwwwhoint/trialsearch/trial2aspx? Trialid=actrn12615001211550. 2015.

35. actrn12616000346471. Can attention training improve sleep, anxiety, and mood? Http://wwwwhoint/trialsearch/trial2aspx? Trialid=actrn12616000346471. 2016.

36. Calear AL, Christensen H, Griffiths KM, MacKinnon A. The Y-Worri Project: Study protocol for a randomised controlled trial. Trials. 2013;14 (1) (no pagination)(76).

37. Gorini A, Riva G. The potential of virtual reality as anxiety management tool: a randomized controlled study in a sample of patients affected by generalized anxiety disorder. Trials. 2008;9:25.

38. Heber E, Ebert DD, Lehr D, Nobis S, Berking M, Riper H. Efficacy and cost-effectiveness of a web-based and mobile stress-management intervention for employees: design of a randomized controlled trial. BMC Public Health. 2013;13:655.

39. Hoek W, Schuurmans J, Koot HM, Cuijpers P. Prevention of depression and anxiety in adolescents: a randomized controlled trial testing the efficacy and mechanisms of Internet-based self-help problem-solving therapy. Trials. 2009;10:93.

40. isrctn32111346. Cognitive-behavioural self-help for depressed and anxious adults; a randomised controlled trial comparing computer based interactive self-help versus a self-help treatment manual. Http://wwwwhoint/trialsearch/trial2aspx? Trialid=isrctn32111346. 2003.

41. isrctn64118207. Randomised controlled trial of computerised psychological treatment of anxiety and depression in primary care. Http://wwwwhoint/trialsearch/trial2aspx? Trialid=isrctn64118207. 2003.

42. isrctn83626400. Using online-cognitive behaviour therapy (Online-CBT) to treat general anxiety and worry among older adults: is it effective and does client engagement matter? Http://wwwwhoint/trialsearch/trial2aspx? Trialid=isrctn83626400. 2013.

43. jprn-umin000001353. Origo - A randomized clinical trial of internet-delivered cognitive behaviour therapy for generalized anxiety disorder. Http://wwwwhoint/trialsearch/trial2aspx? Trialid=jprn-umin000001353. 2008.

44. Nct. Cognitive Training for Mood and Anxiety Disorders. https://clinicaltrialsgov/show/NCT02256566. 2014.

45. Nct. Internet-Based CBT for Children With Anxiety Disorders: Implementation in Clinical Settings. https://clinicaltrialsgov/show/NCT02350257. 2015.

46. Nct. Internet-delivered Transdiagnostic Intervention for Anxiety and Depression. https://clinicaltrialsgov/show/NCT02739607. 2016.

47. nct01390168. Internet-administrated treatment of anxiety disorders (NOVA II). Http://clinicaltrialsgov/ct2/show/nct01390168. 2011.

48. ntr1322. Prevention of depression and anxiety in adolescents through the Internet. Http://wwwwhoint/trialsearch/trial2aspx? Trialid=ntr1322. 2008.

49. ntr3950. Online prevention of anxiety and depression in adolescents. Http://wwwwhoint/trialsearch/trial2aspx? Trialid=ntr3950. 2013.

50. ntr4366. A randomized controlled trial using a video game to reduce anxiety in children. Http://wwwwhoint/trialsearch/trial2aspx? Trialid=ntr4366. 2014.

51. ntr4660. Online training to reduce anxiety and depressive symptoms. Http://wwwwhoint/trialsearch/trial2aspx? Trialid=ntr4660. 2014.

52. NTR4758. Testing daily smartphone-delivered interventions in individuals with workstress. http://wwwwhoint/trialsearch/Trial2aspx?TrialID=NTR4758. 2014.

53. NTR4827. Testing a daily smartphone-delivered intervention in individuals with work stress. http://wwwwhoint/trialsearch/Trial2aspx?TrialID=NTR4827. 2014.

54. ntr4850. Online prevention of anxiety and depression in adolescents - Phase II. Http://wwwwhoint/trialsearch/trial2aspx? Trialid=ntr4850. 2014.

55. Romijn G, Riper H, Kok R, Donker T, Goorden M, van Roijen LH, et al. Cost-effectiveness of blended vs. face-to-face cognitive behavioural therapy for severe anxiety disorders: study protocol of a randomized controlled trial. BMC Psychiatry. 2015;15:311.

56. Titov N. Internet-based treatment of generalized anxiety disorder, and/or social phobia, and/or panic disorder (the Anxiety program): A randomized controlled study exploring the role of a clinician [ACTRN12610000242022]. Australian New Zealand Clinical Trials Registry [wwwanzctrorgau]. 2010.

57. Titov N. A randomized controlled trial of the effects of guided internet-treatment vs. self-guided internet-treatment with pre-treatment contact vs. purely self-guided internet-delivered treatment on symptoms of anxiety and depression in Australian older adults (60+). Http://wwwanzctrorgau/ACTRN12613000958785aspx. 2013.

58. Titov N. A randomized controlled trial of the effects of self-guided vs. guided Internet-administered treatment on symptoms of anxiety and depression in Australian young adults (18-24). Http://wwwanzctrorgau/ACTRN12613000915752aspx. 2013.

59. actrn12610000833066. A randomised controlled trial of a fully automated online mindfulness program focussing on 18-25 Australian Tertiary Education students. Http://wwwwhoint/trialsearch/trial2aspx? Trialid=actrn12610000833066. 2010.

60. actrn12610000247077. The Wellbeing Program: a randomized controlled trial of internet based treatment of anxiety and depression. Http://wwwwhoint/trialsearch/trial2aspx? Trialid=actrn12610000247077. 2010.

61. actrn12606000349549. Computerised cognitive behaviour therapy (CBT) for the treatment of anxiety and effects on neuropsychological functioning and hormone levels. Http://wwwwhoint/trialsearch/trial2aspx? Trialid=actrn12606000349549. 2006.

62. isrctn55102899. Comparing counseling alone versus counseling supplemented with a well-being mobile phone app for university students with anxiety or depression. Http://wwwwhoint/trialsearch/trial2aspx? Trialid=isrctn55102899. 2016.

63. Nct. Impact of a Mobile Application (Pacifica) on Stress, Anxiety, and Depression. https://clinicaltrialsgov/show/NCT03333707. 2017.

64. Repetto C, Gorini A, Algeri D, Vigna C, Gaggioli A, Riva G. The use of biofeedback in clinical virtual reality: The Intrepid Project. Annual Review of CyberTherapy and Telemedicine. 2009;7:128-32.

65. Riva G, Gorini A, Gaggioli A. The Intrepid project - biosensor-enhanced virtual therapy for the treatment of generalized anxiety disorders. Studies in Health Technology and Informatics. 2009;142:271-6.

66. actrn12609000501246. The Anxiety Program: a randomized controlled trial of internet based education for Anxiety. Http://wwwwhoint/trialsearch/trial2aspx? Trialid=actrn12609000501246. 2009.

67. actrn12609000136202. Internet-based education for generalized anxiety disorder (the Worry Program): a randomized controlled trial. Http://wwwwhoint/trialsearch/trial2aspx? Trialid=actrn12609000136202. 2009.

68. Abbott J-AM, Klein B, McLaren S, Austin DW, Molloy M, Meyer D, et al. Out & online; effectiveness of a tailored online multi-symptom mental health and wellbeing program for same-sex attracted young adults: study protocol for a randomised controlled trial. Trials. 2014;15(1):504-.

69. actrn12617000572369. A randomised evaluation trial of the LIFE FLeX digital health and wellbeing program for anxiety and depression. Http://wwwwhoint/trialsearch/trial2aspx? Trialid=actrn12617000572369. 2017.

70. actrn12617000573358. A randomised controlled trial of iMindTime digital health: a comprehensive mindfulness and loving kindness meditation wellbeing digital health program. Http://wwwwhoint/trialsearch/trial2aspx? Trialid=actrn12617000573358. 2017.

71. actrn12617000608369. The Sweet Spot: five levels of support for an anxiety and depression course plus usual care, compared to usual care alone. Http://wwwwhoint/trialsearch/trial2aspx? Trialid=actrn12617000608369. 2017.

72. actrn12617001246370. Use of an Internet-based Decision Aid (myAID) for Ulcerative Colitis Patients to Improve Quality of Life, Empowerment, Decision Making and Disease Control. Http://wwwwhoint/trialsearch/trial2aspx? Trialid=actrn12617001246370. 2017.

73. actrn12617001538336. Online Cognitive Behavioural Therapy (CBT) versus mindfulness for depression and anxiety: a randomised controlled trial. Http://wwwwhoint/trialsearch/trial2aspx? Trialid=actrn12617001538336. 2017.

74. actrn12618001415291. Using Stepped Care in Internet-Based Cognitive Behaviour Therapy for child and adolescent anxiety. Http://wwwwhoint/trialsearch/trial2aspx? Trialid=actrn12618001415291. 2018.

75. ACTRN12618001500246. Comparing two versions of online Cognitive Behavioural Therapy (CBT) for panic and anxiety in adults: a randomised controlled trial. http://wwwwhoint/trialsearch/Trial2aspx?TrialID=ACTRN12618001500246. 2018.

76. actrn12618001565235. Increasing engagement with online programs to improve mental health in the community: the Engagement Project. Http://wwwwhoint/trialsearch/trial2aspx? Trialid=actrn12618001565235. 2018.

77. actrn12618001604291. A randomised control trial of the Uprise online program for university students. Http://wwwwhoint/trialsearch/trial2aspx? Trialid=actrn12618001604291. 2018.

78. actrn12618001688279. Evaluating the effectiveness of a video-based online intervention in reducing depression and anxiety in adults. Http://wwwwhoint/trialsearch/trial2aspx? Trialid=actrn12618001688279. 2018.

79. Bolinski F, Kleiboer A, Karyotaki E, Bosmans JE, Zarski AC, Weisel KK, et al. Effectiveness of a transdiagnostic individually tailored Internet-based and mobile-supported intervention for the indicated prevention of depression and anxiety (ICare Prevent) in Dutch college students: study protocol for a randomised controlled trial. Trials. 2018;19(1):118.

80. chictr-inr-17013188. An intervention study of internet-based cognitive training programs for mental disorders. Http://wwwwhoint/trialsearch/trial2aspx? Trialid=chictr-inr-17013188. 2017.

81. chictr-trc-14005205. A randomized controlled study on the clinical efficacy of brief attentional bias modification training (abmt) in adults with generalized anxiety disorder. Http://wwwwhoint/trialsearch/trial2aspx? Trialid=chictr-trc-14005205. 2014.

82. drks00005384. Online self-help training for work-related stress. Http://wwwwhoint/trialsearch/trial2aspx? Trialid=drks00005384. 2013.

83. Herrero R, Mira A, Cormo G, Etchemendy E, Banos R, Garcia-Palacios A, et al. An Internet based intervention for improving resilience and coping strategies in university students: Study protocol for a randomized controlled trial. Internet Interventions. 2018.

84. Hoek RJA, Havermans BM, Houtman ILD, Brouwers EPM, Heerkens YF, Zijlstra-Vlasveld MC, et al. Stress Prevention@Work: a study protocol for the evaluation of a multifaceted integral stress prevention strategy to prevent employee stress in a healthcare organization: a cluster controlled trial.[Erratum appears in BMC Public Health. 2017 Sep 22;17 (1):736; PMID: 28938882]. BMC Public Health. 2017;18(1):26.

85. isrctn81412545. Investigating the effects of a self-guided, internet-based self-help program for people with anxiety disorders. Http://wwwwhoint/trialsearch/trial2aspx? Trialid=isrctn81412545. 2016.

86. isrctn89276818. Feasibility and preliminary efficacy of digital cognitive behavioural therapy for anxiety. Http://wwwwhoint/trialsearch/trial2aspx? Trialid=isrctn89276818. 2018.

87. isrctn91967124. Internet-delivered interventions for people with depression and anxiety in IAPT services. Http://wwwwhoint/trialsearch/trial2aspx? Trialid=isrctn91967124. 2017.

88. Kader Maideen SF, Mohd-Sidik S, Rampal L, Mukhtar F, Ibrahim N, Phang CK, et al. A Web-Based Psychoeducational Intervention Program for Depression and Anxiety in an Adult Community in Selangor, Malaysia: Protocol of a Randomized Controlled Trial. JMIR Research Protocols. 2016;5(2):e112.

89. Kanuri N, Newman MG, Ruzek JI, Kuhn E, Manjula M, Jones M, et al. The Feasibility, Acceptability, and Efficacy of Delivering Internet-Based Self-Help and Guided Self-Help Interventions for Generalized Anxiety Disorder to Indian University Students: Design of a Randomized Controlled Trial. JMIR Research Protocols. 2015;4(4):e136.

90. Karyotaki E, Klein AM, Riper H, Wit L, Krijnen L, Bol E, et al. Examining the effectiveness of a web-based intervention for symptoms of depression and anxiety in college students: study protocol of a randomised controlled trial. BMJ Open. 2019;9(5):e028739.

91. Kaylor-Hughes CJ, Rawsthorne M, Coulson NS, Simpson S, Simons L, Guo B, et al. Direct to Public Peer Support and e-Therapy Program Versus Information to Aid Self-Management of Depression and Anxiety: Protocol for a Randomized Controlled Trial. JMIR Research Protocols. 2017;6(12):e231.

92. Krahe C, Mathews A, Whyte J, Hirsch CR. Cognitive bias modification for interpretation with and without prior repetitive negative thinking to reduce worry and rumination in generalised anxiety disorder and depression: protocol for a multisession experimental study with an active control condition. BMJ Open. 2016;6(12):e013404.

93. Lutz W, Zimmermann D, Muller V, Deisenhofer AK, Rubel JA. Randomized controlled trial to evaluate the effects of personalized prediction and adaptation tools on treatment outcome in outpatient psychotherapy: study protocol. BMC Psychiatry. 2017;17.

94. Musiat P, Potterton R, Gordon G, Spencer L, Zeiler M, Waldherr K, et al. Web-based indicated prevention of common mental disorders in university students in four European countries - Study protocol for a randomised controlled trial. Internet Interventions. 2018.

95. Nct. Online, Guided Interventions to Reduce Generalized Anxiety Disorder Among Indian University Students. https://clinicaltrialsgov/show/NCT02410265. 2015.

96. Nct. Virtual Reality and Relaxation for the Treatment of Generalized Anxiety Disorders: a Comparative Study With Standard Relaxation. https://clinicaltrialsgov/show/NCT02571790. 2015.

97. Nct. Internet-Delivered Cognitive Behavior Therapy for Anxiety and Depression Amongst French Canadians. https://clinicaltrialsgov/show/NCT03043833. 2017.

98. Nct. Internet-delivered Interventions for Stress, Anxiety and Depression in the Workplace. https://clinicaltrialsgov/show/NCT03271645. 2017.

99. Nct. Internet-delivered CBT for Adolescents With GAD. https://clinicaltrialsgov/show/NCT03469453. 2017.

100. Nct. Efficacy of a Mobile Application vs MBSR in Healthcare Students. https://clinicaltrialsgov/show/NCT03712319. 2018.

101. Nct. Internet-delivered Cognitive Behavioural Therapy in Arabic for Anxiety and Depression. https://clinicaltrialsgov/show/NCT03496350. 2018.

102. nct03552900. Evaluating a Mobile App for Students Seeking Care for Depression and Anxiety at Harvard University Health Services. Https://clinicaltrialsgov/show/nct03552900. 2018.

103. nct03683472. Developing a Novel Digital Therapeutic for the Treatment of Generalized Anxiety Disorder. Https://clinicaltrialsgov/show/nct03683472. 2018.

104. nct03684434. Online Cognitive Behaviour Therapy for Depression and Anxiety: randomized Controlled Trial Varying Treatment Content. Https://clinicaltrialsgov/show/nct03684434. 2018.

105. nct03764644. Web-based Attention Bias Modification Treatment for Childhood Anxiety Disorders. Https://clinicaltrialsgov/show/nct03764644. 2018.

106. nct03771300. Efficacy of a MBI Programme With or Without Virtual Reality Support to Reduce Stress in University Students. Https://clinicaltrialsgov/show/nct03771300. 2018.

107. nct03807193. Self-selected or Predetermined Internet-based Treatment for Generalized Anxiety Disorder With Different Types of Support. Https://clinicaltrialsgov/show/nct03807193. 2019.

108. nct03810131. Study Protocol for a Feasibility Study of a Novel ACT-based eHealth Psychoeducational Intervention for Students With Mental Distress. Https://clinicaltrialsgov/show/nct03810131. 2019.

109. ntr6562. Effectiveness of an Internet-based prevention intervention for depression and anxiety. Http://wwwwhoint/trialsearch/trial2aspx? Trialid=ntr6562. 2017.

110. ntr6797. Examining the effectiveness of a web-based intervention for symptoms of depression and/ or anxiety. Http://wwwwhoint/trialsearch/trial2aspx? Trialid=ntr6797. 2017.

111. NTR6821. Take a DEEP breath: testing the Effectiveness of a Virtual Reality Biofeedback Video Game for Anxiety Regulation. http://wwwwhoint/trialsearch/Trial2aspx?TrialID=NTR6821. 2017.

112. Richards D, Duffy D, Blackburn B, Earley C, Enrique A, Palacios J, et al. Digital IAPT: the effectiveness & cost-effectiveness of internet-delivered interventions for depression and anxiety disorders in the Improving Access to Psychological Therapies programme: study protocol for a randomised control trial. BMC Psychiatry. 2018;18(1):59.

113. TCTR20170727002. A newly developed computer based cognitive behavioral therapy for children with anxiety trait: a pilot study. http://wwwwhoint/trialsearch/Trial2aspx?TrialID=TCTR20170727002. 2017.

114. Weisel KK, Zarski AC, Berger T, Krieger T, Schaub MP, Moser CT, et al. Efficacy and cost-effectiveness of guided and unguided internet- and mobile-based indicated transdiagnostic prevention of depression and anxiety (ICare Prevent): A three-armed randomized controlled trial in four European countries. Internet Interventions. 2018;(no pagination).

115. Williams C, McClay C-A, Martinez R, Morrison J, Haig C, Jones R, et al. Online CBT life skills programme for low mood and anxiety: study protocol for a pilot randomized controlled trial. Trials. 2016;17:1-7.

116. Witlox M, Kraaij V, Garnefski N, de Waal MWM, Smit F, Hoencamp E, et al. An Internet-based Acceptance and Commitment Therapy intervention for older adults with anxiety complaints: study protocol for a cluster randomized controlled trial. Trials. 2018;19(1):502.

**Mixed populations that included GAD among other conditions (e.g. depression) but did not report separate outcomes for the GAD sub-group (n=88)**

1. Batterham PJ, Calear AL, Farrer L, McCallum SM, Cheng VWS. FitMindKit: Randomised controlled trial of an automatically tailored online program for mood, anxiety, substance use and suicidality. Internet Interventions. 2017;(no pagination).

2. Bell CJ, Colhoun HC, Carter FA, Frampton CM. Effectiveness of computerised cognitive behaviour therapy for anxiety disorders in secondary care. Australian and New Zealand Journal of Psychiatry. 2012;46(7):630-40.

3. Bell CJ, Colhoun HC, Carter FA, Frampton CM. Effectiveness of computerised cognitive behaviour therapy for anxiety disorders in secondary care. 2012;46(7):630-40.

4. Benton SA, Heesacker M, Snowden SJ, Lee G. Therapist-assisted, online (TAO) intervention for anxiety in college students: TAO outperformed treatment as usual. Professional Psychology: Research and Practice. 2016;47(5):363-71.

5. Berger T, Boettcher J, Caspar F. Internet-based guided self-help for several anxiety disorders: A randomized controlled trial comparing a tailored with a standardized disorder-specific approach. Psychotherapy. 2014;51(2):207-19.

6. Berger T, Urech A, Krieger T, Stolz T, Schulz A, Vincent A, et al. Effects of a transdiagnostic unguided internet intervention ('velibra') for anxiety disorders in primary care: Results of a randomized controlled trial. Psychological Medicine. 2017;47(1):67-80.

7. Bergman Nordgren L, Carlbring P, Linna E, Andersson G. Role of the working alliance on treatment outcome in tailored internet-based cognitive behavioural therapy for anxiety disorders: randomized controlled pilot trial. JMIR Research Protocols. 2013;2(1):e4.

8. Boettcher J, Astrom V, Pahlsson D, Schenstrom O, Andersson G, Carlbring P. Internet-based mindfulness treatment for anxiety disorders: A randomized controlled trial. Behavior Therapy. 2014;45(2):241-53.

9. Broglia E, Millings A, Barkham M. Counseling With Guided Use of a Mobile Well-Being App for Students Experiencing Anxiety or Depression: Clinical Outcomes of a Feasibility Trial Embedded in a Student Counseling Service. JMIR Mhealth Uhealth. 2019;7(8):e14318.

10. Carlbring P, Maurin L, Torngren C, Linna E, Eriksson T, Sparthan E, et al. Individually-tailored, internet-based treatment for anxiety disorders: A randomized controlled trial. Behaviour Research and Therapy. 2011;49(1):18-24.

11. Comer JS, Puliafico AC, Aschenbrand SG, McKnight K, Robin JA, Goldfine ME, et al. A pilot feasibility evaluation of the CALM program for anxiety disorders in early childhood. Journal of Anxiety Disorders. 2012;26(1):40-9.

12. Day V, McGrath PJ, Wojtowicz M. Internet-based guided self-help for university students with anxiety, depression and stress: A randomized controlled clinical trial. Behaviour Research and Therapy. 2013;51(7):344-51.

13. De Lijster JM, Dieleman GC, Utens EMWJ, Van Der Ende J, Alexander TM, Boon A, et al. Online Attention Bias Modification in Combination with Cognitive-Behavioural Therapy for Children and Adolescents with Anxiety Disorders: A Randomised Controlled Trial. Behaviour Change. 2019.

14. De Voogd EL, Wiers RW, Salemink E. Online visual search attentional bias modification for adolescents with heightened anxiety and depressive symptoms: A randomized controlled trial. Behaviour Research and Therapy. 2017;92:57-67.

15. Dean J, Potts HW, Barker C. Direction to an Internet Support Group Compared With Online Expressive Writing for People With Depression And Anxiety: A Randomized Trial. JMIR Mental Health. 2016;3(2):e12.

16. Dear BF, Fogliati VJ, Fogliati R, Johnson B, Boyle O, Karin E, et al. Treating anxiety and depression in young adults: A randomised controlled trial comparing clinician-guided versus self-guided Internet-delivered cognitive behavioural therapy. Australian and New Zealand Journal of Psychiatry. 2018;52(7):668-79.

17. Dear BF, Zou JB, Ali S, Lorian CN, Johnston L, Sheehan J, et al. Clinical and Cost-Effectiveness of Therapist-Guided Internet-Delivered Cognitive Behavior Therapy for Older Adults With Symptoms of Anxiety: A Randomized Controlled Trial. Behavior Therapy. 2015;46(2):206-17.

18. Donker T, van Straten A, Riper H, Marks I, Andersson G, Cuijpers P. Implementation of Internet-based preventive interventions for depression and anxiety: role of support? The design of a randomized controlled trial. Trials. 2009;10:59.

19. Fish MT, Russoniello CV, O'Brien K. The efficacy of prescribed casual videogame play in reducing symptoms of anxiety: A randomized controlled study. Games for Health. 2014;3(5):291-5.

20. Fitzpatrick KK, Darcy A, Vierhile M. Delivering Cognitive Behavior Therapy to Young Adults With Symptoms of Depression and Anxiety Using a Fully Automated Conversational Agent (Woebot): A Randomized Controlled Trial. JMIR Mental Health. 2017;4(2):e19.

21. Gould CE, Kok BC, Ma VK, Wetherell JL, Sudheimer K, Beaudreau SA. Video-Delivered Relaxation Intervention Reduces Late-Life Anxiety: a Pilot Randomized Controlled Trial. American journal of geriatric psychiatry. 2019.

22. Grime PR. Computerized cognitive behavioural therapy at work: a randomized controlled trial in employees with recent stress-related absenteeism. Occupational Medicine-Oxford. 2004;54(5):353-9.

23. Hadjistavropoulos HD, Schneider LH, Edmonds M, Karin E, Nugent MN, Dirkse D, et al. Randomized controlled trial of Internet-delivered cognitive behaviour therapy comparing standard weekly versus optional weekly therapist support. Journal of Anxiety Disorders. 2017;52:15-24.

24. Haukaas RB, Gjerde IB, Varting G, Hallan HE, Solem S. A Randomized Controlled Trial Comparing the Attention Training Technique and Mindful Self-Compassion for Students With Symptoms of Depression and Anxiety. Frontiers in Psychology. 2018;9.

25. Hoek W, Schuurmans J, Koot HM, Cuijpers P. Effects of Internet-based guided self-help problem-solving therapy for adolescents with depression and anxiety: a randomized controlled trial. PLoS ONE. 2012;7(8):e43485.

26. Hoek W, Schuurmans J, Koot HM, Cuijpers P. Effects of Internet-Based Guided Self-Help Problem-Solving Therapy for Adolescents with Depression and Anxiety: A Randomized Controlled Trial. PLOS ONE. 2012;7(8):e43485.

27. Holmes JM, Spence SH. Feasibility and outcome of clinic-plus-internet delivery of cognitive-behavior therapy for anxious children and their parents. 29th Australian Association for Cognitive and Behaviour Therapy Annual Conference; 2006 October 18 - 23; Manly. 2006:11.

28. Johnston L, Titov N, Andrews G, Spence J, Dear BF. A RCT of a transdiagnostic internet-delivered treatment for three anxiety disorders: examination of support roles and disorder-specific outcomes. PloS one. 2011;6(11):e28079.

29. Johnston L, Titov N, Andrews G, Spence J, Dear BF. A RCT of a transdiagnostic internet-delivered treatment for three anxiety disorders: examination of support roles and disorder-specific outcomes. PloS one. 2011;6(11):e28079.

30. Jolstedt M, Wahlund T, Lenhard F, Ljotsson B, Mataix-Cols D, Nord M, et al. Efficacy and cost-effectiveness of therapist-guided internet cognitive behavioural therapy for paediatric anxiety disorders: a single-centre, single-blind, randomised controlled trial. Lancet Child Adolesc Health. 2018;2(11):792-801.

31. Jonassaint CR, Gibbs P, Belnap BH, Karp JF, Abebe KZ, Rollman BL. Engagement and outcomes for a computerised cognitive-behavioural therapy intervention for anxiety and depression in African Americans. BJPsych Open. 2017;3(1):1-5.

32. Karbasi A, Haratian A. The Efficacy of Internet-based Cognitive Behavioral Therapy on the Anxiety Disorders among Adolescent Girls. Adv. 2018;7:13.

33. Keller ML. An Internet cognitive-behavioral skills-based program for child anxiety. Dissertation Abstracts International: Section B: The Sciences and Engineering. 2010;71(2-B):1344.

34. Kendall PC, Hudson JL, Gosch E, Flannery-Schroeder E, Suveg C. Cognitive-behavioral therapy for anxiety disordered youth: A randomized clinical trial evaluating child and family modalities. Journal of Consulting and Clinical Psychology. 2008;76(2):282-97.

35. Khanna MS, Kendall PC. Computer-assisted cognitive behavioral therapy for child anxiety: results of a randomized clinical trial. Journal of consulting and clinical psychology. 2010;78(5):737.

36. Kleiboer A, Donker T, Seekles W, van Straten A, Riper H, Cuijpers P. A randomized controlled trial on the role of support in internet-based problem solving therapy for depression and anxiety. Behaviour Research and Therapy. 2015;72:63-71.

37. Mailey EL, Wojcicki TR, Motl RW, Hu L, Strauser DR, Collins KD, et al. Internet-delivered physical activity intervention for college students with mental health disorders: A randomized pilot trial. Psychology, Health and Medicine. 2010;15(6):646-59.

38. Mansson KN, Skagius Ruiz E, Gervind E, Dahlin M, Andersson G. Development and initial evaluation of an Internet-based support system for face-to-face cognitive behavior therapy: a proof of concept study. Journal of Medical Internet Research. 2013;15(12):e280.

39. March S, Spence SH, Donovan CL. The efficacy of an internet-based cognitive-behavioral therapy intervention for child anxiety disorders. Journal of pediatric psychology. 2008;34(5):474-87.

40. Marks IM. Randomised controlled trial comparing computerised cognitive behaviour therapy for anxiety and depression against standard GP care. National Research Register. 1998.

41. McCrone P, Knapp M, Proudfoot J, Ryden C, Cavanagh K, Shapiro DA, et al. Cost-effectiveness of computerised cognitive-behavioural therapy for anxiety and depression in primary care: randomised controlled trial. British Journal of Psychiatry. 2004;185:55-62.

42. Moberg C, Niles A, Beermann D. Guided Self-Help Works: Randomized Waitlist Controlled Trial of Pacifica, a Mobile App Integrating Cognitive Behavioral Therapy and Mindfulness for Stress, Anxiety, and Depression. J Med Internet Res. 2019;21(6):e12556.

43. Moore JA. Examination of the effects of computer assisted mindfulness strategies with adolescents in an alternative high school setting. Dissertation Abstracts International: Section B: The Sciences and Engineering. 2018;79(5-B(E)):No-Specified.

44. Mullin A, Dear BF, Karin E, Wootton BM, Staples LG, Johnston L, et al. The UniWellbeing course: A randomised controlled trial of a transdiagnostic internet-delivered cognitive behavioural therapy (CBT) programme for university students with symptoms of anxiety and depression. Internet Interventions. 2015;2(2):128-36.

45. Newby JM, Mackenzie A, Williams AD, McIntyre K, Watts S, Wong N, et al. Internet cognitive behavioural therapy for mixed anxiety and depression: a randomized controlled trial and evidence of effectiveness in primary care. Psychological medicine. 2013;43(12):2635-48.

46. Newby JM, Mackenzie A, Williams AD, McIntyre K, Watts S, Wong N, et al. Internet cognitive behavioural therapy for mixed anxiety and depression: a randomized controlled trial and evidence of effectiveness in primary care. Psychological Medicine. 2013;43(12):2635-48.

47. Newby JM, Mewton L, Andrews G. Transdiagnostic versus disorder-specific internet-delivered cognitive behaviour therapy for anxiety and depression in primary care. Journal of Anxiety Disorders. 2017;46:25-34.

48. Newby JM, Mewton L, Williams AD, Andrews G. Effectiveness of transdiagnostic Internet cognitive behavioural treatment for mixed anxiety and depression in primary care. Journal of Affective Disorders. 2014;165:45-52.

49. Newby JM, Williams AD, Andrews G. Reductions in negative repetitive thinking and metacognitive beliefs during transdiagnostic internet cognitive behavioural therapy (iCBT) for mixed anxiety and depression. Behaviour Research and Therapy. 2014;59:52-60.

50. Niles AN, Loerinc AG, Krull JL, Roy-Byrne P, Sullivan G, Sherbourne CD, et al. Advancing personalized medicine: Application of a novel statistical method to identify treatment moderators in the coordinated anxiety learning and management study. Behavior Therapy. 2017;48(4):490-500.

51. Nordgren LB, Hedman E, Etienne J, Bodin J, Kadowaki A, Eriksson S, et al. Effectiveness and cost-effectiveness of individually tailored Internet-delivered cognitive behavior therapy for anxiety disorders in a primary care population: A randomized controlled trial. Behaviour Research and Therapy. 2014;59:1-11.

52. Proudfoot J, Goldberg D, Mann A, Everitt B, Marks I, Gray JA. Computerized, interactive, multimedia cognitive-behavioural program for anxiety and depression in general practice. Psychological Medicine. 2003;33(2):217-27.

53. Proudfoot J, Ryden C, Everitt B, Shapiro DA, Goldberg D, Mann A, et al. Clinical efficacy of computerised cognitive-behavioural therapy for anxiety and depression in primary care: Randomised controlled trial. The British Journal of Psychiatry. 2004;185(1):46-54.

54. Reid SC, Kauer SD, Hearps SJ, Crooke AH, Khor AS, Sanci LA, et al. A mobile phone application for the assessment and management of youth mental health problems in primary care: a randomised controlled trial. BMC Family Practice. 2011;12:131.

55. Rollman BL. Effectiveness of Online Collaborative Care for Treating Mood and Anxiety Disorders in Primary Care A Randomized Clinical Trial (vol 75, pg 56, 2018). JAMA Psychiatry. 2018;75(1):104-.

56. Rollman BL, Belnap B, Rothenberger SD, Abebe K, Rotondi AJ, Karp JF. ONLINE TREATMENTS FOR MOOD AND ANXIETY DISORDERS IN PRIMARY CARE: A RANDOMIZED CONTROLLED TRIAL. Journal of General Internal Medicine. 2016;31:S316-S7.

57. Rollman BL, Belnap BH, Hum B, Abebe KZ, Spring MB, Rotondi AJ, et al. Effectiveness of online collaborative care for treating mood and anxiety disorders in primary care: A randomized clinical trial. JAMA Psychiatry. 2018;75(1):56-64.

58. Rollman BL, Belnap BH, Rothenberger SD, Abebe KZ, Rotondi AJ, Karp JF. ONLINE TREATMENTS FOR MOOD AND ANXIETY DISORDERS IN PRIMARY CARE: A RANDOMIZED CONTROLLED TRIAL. Psychosomatic Medicine. 2017;79(4):A20-A.

59. Salemink E, Kindt M, Rienties H, van den Hout M. Internet-based Cognitive Bias Modification of Interpretations in patients with anxiety disorders: A randomised controlled trial. Journal of Behavior Therapy and Experimental Psychiatry. 2014;45(1):186-95.

60. Schleider J, Weisz J. A single‐session growth mindset intervention for adolescent anxiety and depression: 9‐month outcomes of a randomized trial. Journal of Child Psychology and Psychiatry. 2018;59(2):160-70.

61. Schleider J, Weisz J. A single-session growth mindset intervention for adolescent anxiety and depression: 9-month outcomes of a randomized trial. Journal of child psychology and psychiatry, and allied disciplines. 2018;59(2):160-70.

62. Scholten H, Malmberg M, Lobel A, Engels RC, Granic I. A Randomized Controlled Trial to Test the Effectiveness of an Immersive 3D Video Game for Anxiety Prevention among Adolescents. PloS one. 2016;11(1):e0147763.

63. Schoneveld EA, Lichtwarck-Aschoff A, Granic I. Preventing Childhood Anxiety Disorders: Is an Applied Game as Effective as a Cognitive Behavioral Therapy-Based Program? Prevention science : the official journal of the Society for Prevention Research. 2018;19(2):220-32.

64. Schoneveld EA, Malmberg M, Lichtwarck-Aschoff A, Verheijen GP, Engels RCME, Granic I. A neurofeedback video game (MindLight) to prevent anxiety in children: A randomized controlled trial. Computers in Human Behavior. 2016;63:321-33.

65. Schuurmans AAT, Nijhof KS, Engels R, Granic I. Using a Videogame Intervention to Reduce Anxiety and Externalizing Problems among Youths in Residential Care: an Initial Randomized Controlled Trial. Journal of psychopathology and behavioral assessment. 2017:1-11.

66. Sethi S. Treating youth depression and anxiety: A randomised controlled trial examining the efficacy of computerised versus face-to-face cognitive behaviour therapy. Australian Psychologist. 2013;48(4):249-57.

67. Sethi S, Campbell AJ, Ellis LA. The use of computerized self-help packages to treat adolescent depression and anxiety. Journal of Technology in Human Services. 2010;28(3):144-60.

68. Shechner T, Rimon-Chakir A, Britton JC, Lotan D, Apter A, Bliese PD, et al. Attention Bias Modification Treatment Augmenting Effects on Cognitive Behavioral Therapy in Children With Anxiety: Randomized Controlled Trial. Journal of the American Academy of Child and Adolescent Psychiatry. 2014;53(1):61-71.

69. Silfvernagel K, Westlinder A, Andersson S, Bergman K, Hernandez RD, Fallhagen L, et al. Individually tailored internet-based cognitive behaviour therapy for older adults with anxiety and depression: A randomised controlled trial. Cognitive Behaviour Therapy. 2018;47(4):286-300.

70. Spence SH, Donovan CL, March S, Gamble A, Anderson RE, Prosser S, et al. A randomized controlled trial of online versus clinic-based CBT for adolescent anxiety. Journal of Consulting and Clinical Psychology. 2011;79(5):629-42.

71. Spence SH, Holmes JM, March S, Lipp OV. The feasibility and outcome of clinic plus internet delivery of cognitive-behavior therapy for childhood anxiety. Journal of consulting and clinical psychology. 2006;74(3):614.

72. Stallard P, Richardson T, Velleman S, Attwood M. Computerized CBT (think, feel, do) for depression and anxiety in children and adolescents: Outcomes and feedback from a pilot randomized controlled trial. Behavioural and Cognitive Psychotherapy. 2011;39(3):273-84.

73. Stallman HM, Kavanagh DJ, Arklay AR, Bennett-Levy J. Randomised control trial of a low-intensity cognitive-behaviour therapy intervention to improve mental health in university students. Australian Psychologist. 2016;51(2):145-53.

74. Staples LG, Fogliati VJ, Dear BF, Nielssen O, Titov N. Internet-delivered treatment for older adults with anxiety and depression: implementation of the Wellbeing Plus Course in routine clinical care and comparison with research trial outcomes. BJPsych Open. 2016;2(5):307-13.

75. Storch EA, Salloum A, King MA, Crawford EA, Andel R, McBride NM, et al. A randomized controlled trial in community mental health centers of computer-assisted cognitive behavioral therapy versus treatment as usual for children with anxiety. Depression and Anxiety. 2015;32(11):843-52.

76. Titov N, Dear BF, Johnston L, Lorian C, Zou J, Wootton B, et al. Improving adherence and clinical outcomes in self-guided internet treatment for anxiety and depression: randomised controlled trial. PLoS ONE. 2013;8(7):e62873.

77. Titov N, Dear BF, Johnston L, McEvoy PM, Wootton B, Terides MD, et al. Improving adherence and clinical outcomes in self-guided internet treatment for anxiety and depression: a 12-month follow-up of a randomised controlled trial. PLoS ONE. 2014;9(2):e89591.

78. Titov N, Dear BF, Schwencke G, Andrews G, Johnston L, Craske MG, et al. Transdiagnostic internet treatment for anxiety and depression: A randomised controlled trial. Behaviour Research and Therapy. 2011;49(8):441-52.

79. Titov N, Fogliati VJ, Staples LG, Gandy M, Johnston L, Wootton B, et al. Treating anxiety and depression in older adults: randomised controlled trial comparing guided v. self-guided internet-delivered cognitive-behavioural therapy. BJPsych Open. 2016;2(1):50-8.

80. Titov N, Fogliati VJ, Staples LG, Gandy M, Johnston L, Wootton B, et al. Treating anxiety and depression in older adults: randomised controlled trial comparing guided v. self-guided internet-delivered cognitive–behavioural therapy. BJPsych Open. 2016;2(1):50-8.

81. Tulbure BT, Rusu A, Sava FA, Sălăgean N, Farchione TJ. A Web-Based Transdiagnostic Intervention for Affective and Mood Disorders: Randomized Controlled Trial. JMIR Ment Health. 2018;5(2):e36.

82. Twomey C, O'Reilly G, Byrne M, Bury M, White A, Kissane S, et al. A randomized controlled trial of the computerized CBT programme, moodGYM, for public mental health service users waiting for interventions. British Journal of Clinical Psychology. 2014;53(4):433-50.

83. Vigerland S, Ljotsson B, Thulin U, Ost L-G, Andersson G, Serlachius E. Internet-delivered cognitive behavioural therapy for children with anxiety disorders: A randomised controlled trial. Behaviour Research and Therapy. 2016;76:47-56.

84. Vigerland S, Serlachius E, Thulin U, Andersson G, Larsson JO, Ljotsson B. Long-term outcomes and predictors of internet-delivered cognitive behavioral therapy for childhood anxiety disorders. Behaviour research and therapy. 2017;90:67-75.

85. Volker D, Zijlstra-Vlasveld MC, Anema JR, Beekman AT, Brouwers EP, Emons WH, et al. Effectiveness of a blended web-based intervention on return to work for sick-listed employees with common mental disorders: results of a cluster randomized controlled trial. Journal of Medical Internet Research. 2015;17(5):e116.

86. Waite P, Marshall T, Creswell C. A randomized controlled trial of internet-delivered cognitive behaviour therapy for adolescent anxiety disorders in a routine clinical care setting with and without parent sessions. Child and Adolescent Mental Health. 2019.

87. Wong SYS, Tang WK, Mak WWS, Cheung FMC, Mercer S, Griffiths SM, et al. Stepped care programme in primary care to prevent anxiety and depression: a randomised clinical trial. Hong Kong Med J. 2019;25(1 Supplement 3).

88. Wuthrich VM, Rapee RM, Cunningham MJ, Lyneham HJ, Hudson JL, Schniering CA. A randomized controlled trial of the Cool Teens CD-ROM computerized program for adolescent anxiety. Journal of the American Academy of Child & Adolescent Psychiatry. 2012;51(3):261-70.

**Did not meet GAD criteria (61)**

1. actrn12616000634471. Mobile Mindfulness Meditation as an Adjunct to Treatment-as-usual: a Randomised Controlled Trial. Http://wwwwhoint/trialsearch/trial2aspx? Trialid=actrn12616000634471. 2016.

2. Armstrong L, Rimes KA. Mindfulness-Based Cognitive Therapy for Neuroticism (Stress Vulnerability): A Pilot Randomized Study. Behavior Therapy. 2016;47(3):287-98.

3. Bakker D, Kazantzis N, Rickwood D, Rickard N. A randomized controlled trial of three smartphone apps for enhancing public mental health. Behaviour Research and Therapy. 2018;109:75-83.

4. Bird T, Mansell W, Wright J, Gaffney H, Tai S. Manage your life online: A web-based randomized controlled trial evaluating the effectiveness of a problem-solving intervention in a student sample. Behavioural and Cognitive Psychotherapy. 2018;46(5):570-82.

5. Bolier L, Ketelaar SM, Nieuwenhuijsen K, Smeets O, Gartner FR, Sluiter JK. Workplace mental health promotion online to enhance well-being of nurses and allied health professionals: A cluster-randomized controlled trial. Internet Interventions. 2014;1(4):196-204.

6. Calear AL, Batterham PJ, Poyser CT, Mackinnon AJ, Griffiths KM, Christensen H. Cluster randomised controlled trial of the e-couch Anxiety and Worry program in schools. Journal of Affective Disorders. 2016;196:210-7.

7. Calear AL, Batterham PJ, Poyser CT, Mackinnon AJ, Griffiths KM, Christensen H. Cluster randomised controlled trial of the e-couch Anxiety and Worry program in schools. Journal of Affective Disorders. 2016;196:210-7.

8. Calear AL, Christensen H, Brewer J, Mackinnon A, Griffiths KM. A pilot randomized controlled trial of the e-couch anxiety and worry program in schools. Internet Interventions. 2016;6:1-5.

9. Calear AL, Christensen H, Mackinnon A, Griffiths KM, O'Kearney R. The YouthMood Project: A cluster randomized controlled trial of an online cognitive behavioral program with adolescents. Journal of Consulting and Clinical Psychology. 2009;77(6):1021-32.

10. Crawford EA, Salloum A, Lewin AB, Andel R, Murphy TK, Storch EA. A pilot study of computer-assisted cognitive behavioral therapy for childhood anxiety in community mental health centers. Journal of Cognitive Psychotherapy. 2013;27(3):221-34.

11. Cukrowicz KC, Joiner Jr TE. Computer-based intervention for anxious and depressive symptoms in a non-clinical population. Cognitive therapy and research. 2007;31(5):677-93.

12. de Voogd EL, Wiers RW, Prins PJM, de Jong PJ, Boendermaker WJ, Zwitser RJ, et al. Online attentional bias modification training targeting anxiety and depression in unselected adolescents: Short- and long-term effects of a randomized controlled trial. Behaviour Research and Therapy. 2016;87:11-22.

13. de Voogd EL, Wiers RW, Zwitser RJ, Salemink E. Emotional working memory training as an online intervention for adolescent anxiety and depression: A randomised controlled trial. Australian Journal of Psychology. 2016;68(3):228-38.

14. de Voogd L, Wiers RW, de Jong PJ, Zwitser RJ, Salemink E. A randomized controlled trial of multi-session online interpretation bias modification training: Short- and long-term effects on anxiety and depression in unselected adolescents. PLoS ONE. 2018;13(3):e0194274.

15. Dennis TA, O'Toole LJ. Mental health on the go: Effects of a gamified attention-bias modification mobile application in trait-anxious adults. Clinical Psychological Science. 2014;2(5):576-90.

16. Dillon A, Kelly M, Robertson IH, Robertson DA. Smartphone applications utilizing biofeedback can aid stress reduction. Frontiers in Psychology. 2016;7.

17. Ellis LA, Campbell AJ, Sethi S, O'Dea BM. Comparative randomized trial of an online cognitive-behavioral therapy program and an online support group for depression and anxiety. Journal of Cybertherapy and Rehabilitation. 2011;4(4):461-7.

18. Eustis EH. "Surviving and thriving during stress": Bridging the gap with technology, A web-based acceptance-based behavioral therapy program for university students. Dissertation Abstracts International: Section B: The Sciences and Engineering. 2018;79(10-B(E)):No-Specified.

19. Eustis EH, Hayes-Skelton SA, Orsillo SM, Roemer L. Surviving and thriving during stress: A randomized clinical trial comparing a brief web-based therapist-assisted acceptance-based behavioral intervention versus waitlist control for college students. Behavior Therapy. 2018;49(6):889-903.

20. Frazier P, Meredith L, Greer C, Paulsen JA, Howard K, Dietz LR, et al. Randomized controlled trial evaluating the effectiveness of a web-based stress management program among community college students. Anxiety, Stress and Coping: An International Journal. 2015;28(5):576-86.

21. Fulmer R, Joerin A, Gentile B, Lakerink L, Rauws M. Using Psychological Artificial Intelligence (Tess) to Relieve Symptoms of Depression and Anxiety: Randomized Controlled Trial. JMIR Mental Health. 2018;5(4):e64.

22. Goodman R, Newman D. Testing a digital storytelling intervention to reduce stress in adolescent females. Storytelling, Self, Society: An Interdisciplinary Journal of Storytelling Studies. 2014;10(2):177-93.

23. Grassi A, Gaggioli A, Riva G. The Green Valley: The Use of Mobile Narratives for Reducing Stress in Commuters. Cyberpsychology and Behavior. 2009;12(2):155-61.

24. Greer C. An online mindfulness intervention to reduce stress and anxiety among college students. Dissertation Abstracts International: Section B: The Sciences and Engineering. 2016;77(3-B(E)):No-Specified.

25. Griffiths KM, Bennett K, Walker J, Goldsmid S, Bennett A. Effectiveness of MH-Guru, a brief online mental health program for the workplace: A randomised controlled trial. Internet Interventions. 2016;6:29-39.

26. Hall BJ, Xiong P, Guo X, Sou EKL, Chou UI, Shen Z. An evaluation of a low intensity mHealth enhanced mindfulness intervention for Chinese university students: A randomized controlled trial. Psychiatry Research. 2018;270:394-403.

27. Hintz S, Frazier PA, Meredith L. Evaluating an online stress management intervention for college students. Journal of Counseling Psychology. 2015;62(2):137-47.

28. isrctn55164794. Investigating the effectiveness of a working memory training intervention to increase educational achievement and reduce anxiety in young people. Http://wwwwhoint/trialsearch/trial2aspx? Trialid=isrctn55164794. 2011.

29. Lee RA, Jung ME. Evaluation of an mHealth App (DeStressify) on University Students' Mental Health: Pilot Trial. JMIR Mental Health. 2018;5(1):e2.

30. Levin ME. Evaluating a prototype acceptance and commitment training web-based prevention program for depression and anxiety in college students. Dissertation Abstracts International: Section B: The Sciences and Engineering. 2014;75(1-B(E)):No-Specified.

31. Levin ME, Haeger JA, Pierce BG, Twohig MP. Web-based acceptance and commitment therapy for mental health problems in college students: A randomized controlled trial. Behavior Modification. 2017;41(1):141-62.

32. Levin ME, Pistorello J, Seeley JR, Hayes SC. Feasibility of a prototype web-based acceptance and commitment therapy prevention program for college students. Journal of American College Health. 2014;62(1):20-30.

33. Ma Y, She Z, Siu AF, Zeng X, Liu X. Effectiveness of Online Mindfulness-Based Interventions on Psychological Distress and the Mediating Role of Emotion Regulation. Frontiers in Psychology. 2018;9:2090.

34. Manicavasagar V, Horswood D, Burckhardt R, Lum A, Hadzi-Pavlovic D, Parker G. Feasibility and effectiveness of a web-based positive psychology program for youth mental health: Randomized controlled trial. Journal of Medical Internet Research. 2014;16(6):23-39.

35. Melnyk BM, Amaya M, Szalacha LA, Hoying J, Taylor T, Bowersox K. Feasibility, acceptability, and preliminary effects of the COPE online cognitive-behavioral skill-building program on mental health outcomes and academic performance in freshmen college students: A randomized controlled pilot study. Journal of Child and Adolescent Psychiatric Nursing. 2015;28(3):147-54.

36. Musiat P. Trait-focused internet-based prevention of common mental disorders in students [Ph.D.]. Ann Arbor: University of London, King's College (United Kingdom); 2012.

37. Musiat P, Conrod P, Treasure J, Tylee A, Williams C, Schmidt U. Targeted prevention of common mental health disorders in university students: randomised controlled trial of a transdiagnostic trait-focused web-based intervention. PLoS ONE. 2014;9(4):e93621.

38. Nct. The Efficacy of a Mobile Application for Treating Depression and Anxiety Symptoms. https://clinicaltrialsgov/show/NCT03032952. 2017.

39. Querstret D, Cropley M, Fife-Schaw C. The Effects of an Online Mindfulness Intervention on Perceived Stress, Depression and Anxiety in a Non-clinical Sample: A Randomised Waitlist Control Trial. Mindfulness. 2018;9(6):1825-36.

40. Saleh D, Camart N, Sbeira F, Romo L. Can we learn to manage stress? A randomized controlled trial carried out on university students. PLoS ONE. 2018;13(9):e0200997.

41. van Straten A, Cuijpers P, Smits N. Effectiveness of a web-based self-help intervention for symptoms of depression, anxiety, and stress: Randomized controlled trial. Journal of Medical Internet Research. 2008;10(1):80-9.

42. van't Veer-Tazelaar PJ, van Marwijk HWJ, van Oppen P, van Hout HPJ, van der Horst HE, Cuijpers P, et al. Stepped-care prevention of anxiety and depression in late life: a randomized controlled trial. Archives of general psychiatry. 2009;66(3):297-304.

43. Versluis A, Verkuil B, Brosschot JF. Reducing worry and subjective health complaints: A randomized trial of an internet-delivered worry postponement intervention. British Journal of Health Psychology. 2016;21(2):318-35.

44. Viskovich S, Pakenham KI. Pilot evaluation of a web-based acceptance and commitment therapy program to promote mental health skills in university students. Journal of Clinical Psychology. 2018;74(12):2047-69.

45. Wong N, Kady L, Mewton L, Sunderland M, Andrews G. Preventing anxiety and depression in adolescents: A randomised controlled trial of two school based Internet-delivered cognitive behavioural therapy programmes. Internet Interventions. 2014;1(2):90-4.

46. Balconi M, Fronda G, Crivelli D. Effects of technology-mediated mindfulness practice on stress: psychophysiological and self-report measures. Stress (Amsterdam, Netherlands). 2018;(no pagination).

47. Bostock S, Crosswell AD, Prather AA, Steptoe A. Mindfulness on-the-go: Effects of a mindfulness meditation app on work stress and well-being. Journal of occupational health psychology. 2018.

48. Ebert DD, Heber E, Berking M, Riper H, Cuijpers P, Funk B, et al. Self-guided internet-based and mobile-based stress management for employees: results of a randomised controlled trial. Occup Environ Med. 2016;73(5):315-23.

49. Ebert DD, Kahlke F, Buntrock C, Berking M, Smit F, Heber E, et al. A health economic outcome evaluation of an internet-based mobile supported stress management intervention for employees. Scandinavian Journal of Work, Environment & Health. 2018;44(2):171-82.

50. Ebert DD, Lehr D, Heber E, Riper H, Cuijpers P, Berking M. Internet- and mobile-based stress management for employees with adherence-focused guidance: efficacy and mechanism of change. Scand J Work Environ Health. 2016;42(5):382-94.

51. Floyd M, McKendree-Smith N, Bailey E, Stump J, Scogin F, Bowman D. Two-year follow-up of self-examination therapy for generalized anxiety disorder. Journal of anxiety disorders. 2002;16(4):369-75.

52. Heber E, Lehr D, Ebert DD, Berking M, Riper H. Web-Based and Mobile Stress Management Intervention for Employees: A Randomized Controlled Trial. Journal of medical Internet research. 2016;18(1):e21.

53. Herrero R, Mira A, Cormo G, Etchemendy E, Baños R, García-Palacios A, et al. An Internet based intervention for improving resilience and coping strategies in university students: Study protocol for a randomized controlled trial. Internet Interventions. 2019;16:43-51.

54. Kenardy J, McCafferty K, Rosa V. Internet-delivered indicated prevention for anxiety disorders: A randomized controlled trial. Behavioural and cognitive psychotherapy. 2003;31(3):279-89.

55. Kenardy J, McCafferty K, Rosa V. Internet-delivered indicated prevention for anxiety disorders: Six-month follow-up. Clinical Psychologist. 2006;10(1):39-42.

56. Mistretta EG, Davis MC, Temkit Mh, Lorenz C, Darby B, Stonnington CM. Resilience training for work-related stress among health care workers: results of a randomized clinical trial comparing in-person and smartphone-delivered interventions. Journal of occupational and environmental medicine. 2018;60(6):559-68.

57. Nct. Transdiagnostic iCBT for Depression and Anxiety. https://clinicaltrialsgov/show/NCT02266693. 2014.

58. Nct. Internet-delivered Cognitive Behaviour Therapy for Older Adults With Anxiety. https://clinicaltrialsgov/show/NCT02403557. 2015.

59. Norr AM, Gibby BA, Schmidt NB. Is computerized psychoeducation sufficient to reduce anxiety sensitivity in an at-risk sample?: A randomized trial. Journal of affective disorders. 2017;212:48-55.

60. Versluis A, Verkuil B, Spinhoven P, Brosschot JF. Effectiveness of a smartphone-based worry-reduction training for stress reduction: A randomized-controlled trial. Psychology and Health. 2018;33(9):1079-99.

61. Wiegand B, Luedtke K, Friscia D, Nair M, Aleles M, McCloskey R. Efficacy of a comprehensive program for reducing stress in women: a prospective, randomized trial. Current medical research and opinion. 2010;26(4):991-1002.

**Not an RCT (18)**

1. actrn12613000915752. What is the efficacy of therapist-guided and self-guided internet-delivered treatment for young adults (18-24) with symptoms of anxiety and depression? Http://wwwwhoint/trialsearch/trial2aspx? Trialid=actrn12613000915752. 2013.

2. actrn12613000958785. What is the efficacy of therapist-guided internet-treatment, self-guided internet-treatment with pre-treatment contact, and purely self-guided internet-delivered for older adults (60+) with symptoms of anxiety and depression? Http://wwwwhoint/trialsearch/trial2aspx? Trialid=actrn12613000958785. 2013.

3. Alavi N, Stefanoff M, Hirji A, Khalid-Khan S. Cognitive Behavioural Therapy through PowerPoint: Efficacy in an Adolescent Clinical Population with Depression and Anxiety. International Journal of Pediatrics. 2018.

4. Almlov J, Carlbring P, Kallqvist K, Paxling B, Cuijpers P, Andersson G. Therapist effects in guided internet-delivered CBT for anxiety disorders. Behavioural and cognitive psychotherapy. 2011;39(3):311-22.

5. Almlöv J, Carlbring P, Källqvist K, Paxling B, Cuijpers P, Andersson G. Therapist effects in guided Internet-delivered CBT for anxiety disorders. Behavioural and Cognitive Psychotherapy. 2011;39(3):311-22.

6. Anonymous. Erratum: Cost-effectiveness of computerised cognitive-behavioural therapy for anxiety and depression in primary care: Randomised controlled trial (British Journal of Psychiatry 185 (55-62)). British Journal of Psychiatry. 2004;185(NOV.):440.

7. Anstiss D, Davies A. ‘Reach Out, Rise Up’: The efficacy of text messaging in an intervention package for anxiety and depression severity in young people. Children and Youth Services Review. 2015;58:99-103.

8. Cavanagh K, Seccombe N, Lidbetter N. The implementation of computerized cognitive behavioural therapies in a service user-led, third sector self help clinic. Behavioural and Cognitive Psychotherapy. 2011;39(4):427-42.

9. Dahlin M, Ryberg M, Vernmark K, Annas N, Carlbring P, Andersson G. Internet-delivered acceptance-based behavior therapy for generalized anxiety disorder: A pilot study. Internet Interventions. 2016;6:16-21.

10. Dear BF, Zou JB, Ali S, Lorian CN, Johnston L, Terides MD, et al. Examining self-guided internet-delivered cognitive behavior therapy for older adults with symptoms of anxiety and depression: Two feasibility open trials. Internet Interventions. 2015;2(1):17-23.

11. Donovan CL, Spence SH, March S. Does an online CBT program for anxiety impact upon sleep problems in anxious youth? Journal of Clinical Child and Adolescent Psychology. 2017;46(2):211-21.

12. Drake KL, Stewart CE, Muggeo MA, Ginsburg GS. Enhancing the Capacity of School Nurses to Reduce Excessive Anxiety in Children: Development of the CALM Intervention. J Child Adolesc Psychiatr Nurs. 2015;28(3):121-30.

13. Heilman RM, Kallay E, Miclea M. The role of computer-based psychotherapy in the treatment of anxiety disorders. Cognition, Brain, Behavior: An Interdisciplinary Journal. 2010;14(3):209-30.

14. Knox M, Lentini J, Cummings T, McGrady A, Whearty K, Sancrant L. Game-based biofeedback for paediatric anxiety and depression. Mental Health in Family Medicine. 2011;8(3):195-203.

15. Lee Y-C, Gao L, Dear BF, Titov N, Mihalopoulos C. The cost-effectiveness of the online MindSpot Clinic for the treatment of depression and anxiety in Australia. Journal of Mental Health Policy and Economics. 2017;20(4):155-66.

16. Mahoney AEJ, Newby JM, Hobbs MJ, Williams AD, Andrews G. Reducing behavioral avoidance with internet-delivered cognitive behavior therapy for generalized anxiety disorder. Internet Interventions. 2019;15:105-9.

17. Palacios JE, Richards D, Palmer R, Coudray C, Hofmann SG, Palmieri PA, et al. Supported Internet-Delivered Cognitive Behavioral Therapy Programs for Depression, Anxiety, and Stress in University Students: Open, Non-Randomised Trial of Acceptability, Effectiveness, and Satisfaction. JMIR Mental Health. 2018;5(4):e11467.

18. Salloum A, Andel R, Lewin AB, Johnco C, McBride NM, Storch EA. Family accommodation as a predictor of cognitive-behavioral treatment outcome for childhood anxiety. Families in Society. 2018;99(1):45-55.

**Reviews (18)**

1. Andrews G, Basu A, Cuijpers P, Craske MG, McEvoy P, English CL, et al. Computer therapy for the anxiety and depression disorders is effective, acceptable and practical health care: An updated meta-analysis. Journal of Anxiety Disorders. 2018;55:70-8.

2. Andrews G, Cuijpers P, Craske MG, McEvoy P, Titov N. Computer therapy for the anxiety and depressive disorders is effective, acceptable and practical health care: a meta-analysis. PLoS ONE. 2010;5(10):e13196.

3. Arnberg FK, Linton SJ, Hultcrantz M, Heintz E, Jonsson U. Internet-delivered psychological treatments for mood and anxiety disorders: a systematic review of their efficacy, safety, and cost-effectiveness. PLoS ONE. 2014;9(5):e98118.

4. Ashford MT, Olander EK, Ayers S. Finding Web-Based Anxiety Interventions on the World Wide Web: A Scoping Review. Jmir Mental Health. 2016;3(2).

5. Calear AL, Christensen H. Review of internet-based prevention and treatment programs for anxiety and depression in children and adolescents. Medical Journal of Australia. 2010;192(11 Suppl):S12-4.

6. Christensen H, Batterham P, Calear A. Online interventions for anxiety disorders. Current Opinion in Psychiatry. 2014;27(1):7-13.

7. Cuijpers P, Marks IM, van Straten A, Cavanagh K, Gega L, Andersson G. Computer-aided psychotherapy for anxiety disorders: a meta-analytic review. Cognitive Behaviour Therapy\. 2009;38(2):66-82\.

8. Deady M, Choi I, Calvo RA, Glozier N, Christensen H, Harvey SB. eHealth interventions for the prevention of depression and anxiety in the general population: a systematic review and meta-analysis. BMC Psychiatry. 2017;17(1):310.

9. Ebert DD, Zarski AC, Christensen H, Stikkelbroek Y, Cuijpers P, Berking M, et al. Internet and computer-based cognitive behavioral therapy for anxiety and depression in youth: a meta-analysis of randomized controlled outcome trials. PLoS ONE. 2015;10(3):e0119895.

10. Fodor LA, Cotet CD, Cuijpers P, Szamoskozi S, David D, Cristea IA. The effectiveness of virtual reality based interventions for symptoms of anxiety and depression: A meta-analysis. Sci. 2018;8(1):10323.

11. Gee BL, Griffiths KM, Gulliver A. Effectiveness of mobile technologies delivering Ecological Momentary Interventions for stress and anxiety: A systematic review. Journal of the American Medical Informatics Association. 2016;23(1):221-9.

12. Grist R, Croker A, Denne M, Stallard P. Technology Delivered Interventions for Depression and Anxiety in Children and Adolescents: A Systematic Review and Meta-analysis. Clinical Child and Family Psychology Review. 2018;18:18.

13. Health Quality O. Internet-Delivered Cognitive Behavioural Therapy for Major Depression and Anxiety Disorders: A Health Technology Assessment. Ontario Health Technology Assessment Series. 2019;19(6):1-199.

14. Pennant ME, Loucas CE, Whittington C, Creswell C, Fonagy P, Fuggle P, et al. Computerised therapies for anxiety and depression in children and young people: A systematic review and meta-analysis. Behaviour Research and Therapy. 2015;67:1-18.

15. Reger MA, Gahm GA. A meta-analysis of the effects of Internet- and computer-based cognitive-behavioral treatments for anxiety. Journal of Clinical Psychology\. 2009;65(1):53-75\.

16. Richards D, Richardson T, Timulak L, McElvaney J. The efficacy of internet-delivered treatment for generalized anxiety disorder: A systematic review and meta-analysis. Internet Interventions. 2015;2(3):272-82.

17. Richardson T, Stallard P, Velleman S. Computerised cognitive behavioural therapy for the prevention and treatment of depression and anxiety in children and adolescents: A systematic review. Clinical Child and Family Psychology Review. 2010;13(3):275-90.

18. Ye X, Bapuji SB, Winters SE, Struthers A, Raynard M, Metge C, et al. Effectiveness of internet-based interventions for children, youth, and young adults with anxiety and/or depression: a systematic review and meta-analysis. BMC Health Services Research. 2014;14:313.

**Abstracts only (14)**

1. Balconi M, Fronda G, Venturella I, Crivelli D. Fostering adaptive stress management via technology-mediated mindfulness practice: self-report and psychophysiological evidence. Annals of general psychiatry. 2018;17.

2. Belnap BH, Abebe KZ, Karp J, Rollman BL. Use and benefits of computerized CBT integrated in a collaborative care program for anxious and depressed primary care patients. Psychosomatic Medicine. 2015;77 (3):A17.

3. Harrer M, Adam SH, Fleischmann RJ, Baumeister H, Auerbach R, Bruffaerts R, et al. Effectiveness of an Internet- and App-Based Intervention for College Students With Elevated Stress: Randomized Controlled Trial. Journal of Medical Internet Research. 2018;20(4):1-.

4. Houghton VT. A quantitative study of the effectiveness of mindfulness-based stress reduction treatment, using an internet-delivered self-help program, for women with generalized anxiety disorder. Dissertation Abstracts International: Section B: The Sciences and Engineering. 2008;69(5-B):3311.

5. March S, Donovan C, Spence S, Anderson R, Prosser S, Kenardy J. Online therapy for youth anxiety works! An overview of the evidence for brave-online and predictors of therapy outcome. Neuropsychiatrie de l'Enfance et de l'Adolescence. 2012;1):S61.

6. Ong LP, Ong SH, Yeo K. A Pilot Study on the Integration of a Cognitive-Behavioral Therapy-Based Computer Game in the Clinical Treatment of Childhood Anxiety. Annals of the Academy of Medicine Singapore (S327 pages). 2013;42:S56.

7. Paxling B, Almlöv J, Dahlin M, Carlbring P, Breitholtz E, Eriksson T, et al. A randomized controlled study of the efficacy of an internet-delivered guided self-help treatment for generalized anxiety disorder [unpublished manuscript]. 2011.

8. Reid S, Kauer S, Patton G. Using cell phones to detect, treat, and manage adolescent mental health: a randomised controlled trial of the mobiletype program in rural and metro primary care Australia. Journal of adolescent health. 2011;1)(2):S96-S7.

9. Rosenberger EM, Belnap BH, Abebe K, Rothenberger SD, Rollman BL. Engagement in an internet support group for mood and anxiety disorders is associated with improved psychiatric outcomes. Journal of General Internal Medicine. 2017;32 (2 Supplement 1):S181.

10. Hemenway Eustis E. "Surviving and Thriving during Stress": Bridging the Gap with Technology, a Web-Based Acceptance-Based Behavioral Therapy Program for Uni versity Students. Surviving and Thriving During Stress: Bridging the Gap With Technology, a Web-Based Acceptance-Based Behavioral Therapy Program for University Students. 2018:1-.

11. Gorini A, Riva G. The Potential of Virtual Reality as Anxiety Management Tool: A Randomized Controlled Study in a Sample of Patients Affected by Generalized Anxiety Disorder. Cyberpsychology and Behavior. 2009;12(1):110-.

12. Querstret D, Cropley M, Fife-Schaw C. ASSESSING THE EFFECTIVENESS OF AN INTERNET-BASED INSTRUCTOR-LED MINDFULNESS INTERVENTION FOR REDUCING STRESS, DEPRESSION AND ANXIETY: A RANDOMISED WAITLIST CONTROL TRIAL. International Journal of Behavioral Medicine. 2016;23:S54-S5.

13. Rollman BL, Belnap BH, Rothenberger SD, Abebe K, Rotondi AJ, Karp JF. Online treatments for mood and anxiety disorders in primary care: A randomized controlled trial. Journal of General Internal Medicine. 2016;1):S316-S7.

14. Bostock SK, Steptoe A. Can finding headspace reduce work stress? A randomised controlled workplace trial of a mindfulness meditation app. Psychosomatic Medicine. 2013;75 (3):A36-A7.

**No anxiety outcome (11)**

1. A health economic outcome evaluation of an internet-based mobile-supported stress management intervention for employees. Scandinavian journal of work, environment & health. 2018;44(2):171-82.

2. actrn12615000820505. A randomised controlled trial and consumer characteristics of iMindTime eHealth: a comprehensive mindfulness and loving kindness meditation wellbeing online program. Http://wwwwhoint/trialsearch/trial2aspx? Trialid=actrn12615000820505. 2015.

3. Carter FA, Bell CJ, Colhoun HC. Suitability and acceptability of computerised cognitive behaviour therapy for anxiety disorders in secondary care. Australian and New Zealand Journal of Psychiatry. 2013;47(2):142-52.

4. Cavanagh K, Churchard A, O'Hanlon P, Mundy T, Votolato P, Jones F, et al. A Randomised Controlled Trial of a Brief Online Mindfulness-Based Intervention in a Non-clinical Population: Replication and Extension. Mindfulness. 2018;9(4):1191-205.

5. Cavanagh K, Strauss C, Cicconi F, Griffiths N, Wyper A, Jones F. A randomised controlled trial of a brief online mindfulness-based intervention. Behaviour Research and Therapy. 2013;51(9):573-8.

6. Hersch RK, Cook RF, Deitz DK, Kaplan S, Hughes D, Friesen MA, et al. Reducing nurses' stress: A randomized controlled trial of a web-based stress management program for nurses. Applied Nursing Research. 2016;32:18-25.

7. Kählke F, Buntrock C, Smit F, Berking M, Lehr D, Heber E, et al. Economic Evaluation of an Internet-Based Stress Management Intervention Alongside a Randomized Controlled Trial. JMIR Ment Health. 2019;6(5):e10866.

8. Lappalainen P, Kaipainen K, Lappalainen R, Hoffren H, Myllymaki T, Kinnunen ML, et al. Feasibility of a personal health technology-based psychological intervention for men with stress and mood problems: randomized controlled pilot trial. JMIR Research Protocols. 2013;2(1):e1.

9. Levin ME, Navarro C, Cruz RA, Haeger J. Comparing in-the-moment skill coaching effects from tailored versus non-tailored acceptance and commitment therapy mobile apps in a non-clinical sample. Cognitive Behaviour Therapy. 2018:1-17.

10. Nct. Internet-delivered Psychodynamic Therapy for Depression and Anxiety Disorders. https://clinicaltrialsgov/show/NCT01532219. 2012.

11. Salemink E, van den Hout M, Kindt M. Effects of positive interpretive bias modification in highly anxious individuals. Journal of Anxiety Disorders. 2009;23(5):676-83.

**Intervention not software-based (10)**

1. Craske MG, Stein MB, Sullivan G, Sherbourne C, Bystritsky A, Rose RD, et al. Disorder-Specific Impact of Coordinated Anxiety Learning and Management Treatment for Anxiety Disorders in Primary Care. Archives of General Psychiatry. 2011;68(4):378-88.

2. Dean JH. Online support groups for depression and anxiety: outcome and process [Ph.D.]. Ann Arbor: University of London, University College London (United Kingdom); 2014.

3. actrn12613000480785. Evaluation of an online parenting programme for parents of 3-4 year-old children. Http://wwwwhoint/trialsearch/trial2aspx? Trialid=actrn12613000480785. 2013.

4. actrn12614000672651. A web-based randomised controlled trial exploring the impact of telephone-based practitioner support in an online parenting program. Http://wwwwhoint/trialsearch/trial2aspx? Trialid=actrn12614000672651. 2014.

5. LaFreniere LS, Newman MG. A Brief Ecological Momentary Intervention for Generalized Anxiety Disorder: A Randomized Controlled Trial of the Worry Outcome Journal. Depression and Anxiety. 2016;33(9):829-39.

6. Li F, Xiong YK. Application of music therapy combined with computer biofeedback in the treatment of anxiety disorders. Li S, Dai Y, Cheng Y, editors2016. 90-3 p.

7. actrn12613001150730. How to be Brave; Program to treat specific phobia in children and Prevent anxiety and depression in adolescence and adulthood. Http://wwwwhoint/trialsearch/trial2aspx? Trialid=actrn12613001150730. 2013.

8. Hayes-Skelton SA, Roemer L, Orsillo SM. A randomized clinical trial comparing an acceptance-based behavior therapy to applied relaxation for generalized anxiety disorder. Journal of consulting and clinical psychology. 2013;81(5):761.

9. Leichsenring D Sc F, Salzer S, Jaeger U, Kächele H, Kreische R, Leweke F, et al. Short-term psychodynamic psychotherapy and cognitive-behavioral therapy in generalized anxiety disorder: a randomized, controlled trial. American Journal of Psychiatry. 2009;166(8):875-81.

10. Roemer L, Orsillo SM, Salters-Pedneault K. Efficacy of an acceptance-based behavior therapy for generalized anxiety disorder: Evaluation in a randomized controlled trial. Journal of consulting and clinical psychology. 2008;76(6):1083.

**Not a GAD population (6)**

1. Loughnan SA, Sie A, Hobbs MJ, et al. A randomized controlled trial of 'MUMentum Pregnancy': Internet-delivered cognitive behavioral therapy program for antenatal anxiety and depression. Journal of Affective Disorders 2019;243:381-90 doi: http://dx.doi.org/10.1016/j.jad.2018.09.057[published Online First: Epub Date]|.

2. Amir N, Taylor CT. Interpretation training in individuals with generalized social anxiety disorder: A randomized controlled trial. Journal of Consulting and Clinical Psychology 2012;80(3):497-511 doi: http://dx.doi.org/10.1037/a0026928[published Online First: Epub Date]|.

3. Amir N, Taylor CT. "Interpretation training in individuals with generalized social anxiety disorder: A randomized controlled trial": Correction to Amir and Taylor (2012). Journal of Consulting and Clinical Psychology 2013;81(1):74 doi: http://dx.doi.org/10.1037/a0031156[published Online First: Epub Date]|.

4. Dolezal-Wood S, Belar CD, Snibbe J. A comparison of computer-assisted psychotherapy and cognitive-behavioral therapy in groups. Journal of Clinical Psychology in Medical Settings 1998;5(1):103-15 doi: http://dx.doi.org/10.1023/A:1026210020906[published Online First: Epub Date]|.

5. Nct. Mobile Phone App for Depression and Anxiety in Young Men Who Are Attracted to Men. https://clinicaltrials.gov/show/NCT02072252 2014

6. Nct. Mindfulness Based Resilience Training. https://clinicaltrials.gov/show/NCT02419430 2015
