# Supplementary material for: Digital Interventions for Generalized Anxiety Disorder (GAD): Systematic Review and Network Meta-Analysis
Source: Front Psychiatry. 2021 Dec 6;12:726222. doi: 10.3389/fpsyt.2021.726222 (PMC8685377; doi:10.3389/fpsyt.2021.726222)
Supplement: Supplementary file 5 [file Data_Sheet_5.docx]

**Appendix E Risk of Bias Assessment in RCTs of DIs for GAD**

**E1. Visual representation of percentage of studies that scored ‘low, high or some’ risk across the 6 domains of the Risk of Bias Assessment Tool**

**E2. Visual representation of each study and how it scored (‘low, high or some’ risk) in each domain of the Risk of Bias Assessment Tool**
