# Supplementary material for: Digital Interventions for Generalized Anxiety Disorder (GAD): Systematic Review and Network Meta-Analysis
Source: Front Psychiatry. 2021 Dec 6;12:726222. doi: 10.3389/fpsyt.2021.726222 (PMC8685377; doi:10.3389/fpsyt.2021.726222)
Supplement: Supplementary file 6 [file Data_Sheet_6.docx]

**APPENDIX F Outcome measures used in RCTs for comparisons between DIs and alternatives**

**F1. Outcome measures used in at least 2 studies**

|  | GAD-7 | PSWQ | PHQ-9 | BDI / BDI-II | STAI-T | BAI | STAI-S | MADRS-S | PDSS-SR | GADQ-IV | QOLI | K-10 | SDS | NEO-FFI-N | RRS | HADS/ HADS-A | MINI | CGI | ASI / ASI-3 | CES-D |
| --- | --- | --- | --- | --- | --- | --- | --- | --- | --- | --- | --- | --- | --- | --- | --- | --- | --- | --- | --- | --- |
| Andersson 2012 |  | x |  | x | x | x | x | x |  | x | x |  |  |  |  |  |  | x |  |  |
| Andersson 2016 |  | x |  |  |  |  |  | x |  |  |  |  |  |  |  | x |  |  |  |  |
| Christensen 2014a | x | x |  |  |  |  |  |  |  |  |  |  |  |  |  |  | x |  | x | x |
| Christensen 2014b | x |  |  |  |  |  |  |  |  |  |  |  |  |  |  |  |  | x |  | x |
| Dahlin 2016 | x | x | x |  |  | x |  | x |  | x | x |  |  |  |  |  |  |  |  |  |
| Dear 2015 | x |  | x |  |  |  |  |  | x |  |  |  |  | x |  |  | x |  |  |  |
| Hazen 2009 |  | x |  | x | x |  |  |  |  |  |  |  |  |  |  |  |  |  |  |  |
| Hirsch 2018 | x | x | x |  |  |  |  |  |  |  |  |  |  |  | x |  |  |  |  |  |
| Howell 2018 | x |  |  |  |  |  |  |  |  |  |  |  |  |  |  |  |  |  |  |  |
| Johansson 2013 | x |  | x |  |  |  |  |  |  |  |  |  |  |  |  |  |  |  |  |  |
| Jones 2016 | x |  | **x** |  |  |  |  |  |  |  |  |  |  |  |  |  |  |  |  |  |
| Navarro-Haro 2019 | x |  |  |  |  |  |  |  |  |  |  |  |  |  |  | x |  |  |  |  |
| Paxling 2011 |  | x |  | x | x | x | x | x |  | x | x |  |  |  |  |  |  |  |  |  |
| Pham 2016 | x |  |  |  |  |  |  |  | x |  |  |  |  |  |  |  |  |  | x |  |
| Repetto 2013 | x | x |  |  | x | x | x |  |  |  |  |  |  |  |  |  |  |  |  |  |
| Richards 2016 | x | x |  | x |  |  |  |  |  |  |  |  |  |  |  |  |  |  |  |  |
| Robinson 2010 | x | x | x |  |  |  |  |  |  |  |  | x | x |  |  |  |  |  |  |  |
| Teng 2019 |  | x |  | x | x | x | x |  |  |  |  |  |  |  |  |  |  |  |  |  |
| Titov 2009 | x | x | x |  |  |  |  |  |  |  |  | x | x |  |  |  |  |  |  |  |
| Titov 2010 |  | x | x |  |  |  |  |  | x |  |  | x | x | x |  |  |  |  |  |  |
| Topper 2017 |  | x |  | x |  |  |  |  |  | x |  |  |  |  | x |  |  |  |  |  |
| **Total no of comparisons** | 14 | 14 | 8 | 6 | 5 | 5 | 4 | 4 | 3 | 4 | 3 | 3 | 3 | 2 | 2 | 2 | 2 | 2 | 2 | 2 |

**F2. Outcome measures used in only 1 study**

|  | PSWQ-A | SCID-I | CAQ | BBQ | MCQ-30 | IOU | Days out of role | MINI-SPIN | GAI | GDS | WHOQOL | ACES | FFMQ | DERS | MAIA | OASIS | QLES-Q-SF | HAM-A | WASA | SPSQ | DASS-21 | PTQ | MASQ-D30 | EDI-2-BU | QDS |
| --- | --- | --- | --- | --- | --- | --- | --- | --- | --- | --- | --- | --- | --- | --- | --- | --- | --- | --- | --- | --- | --- | --- | --- | --- | --- |
| Andersson 2012 |  | x |  |  |  |  |  |  |  |  |  |  |  |  |  |  |  |  |  |  |  |  |  |  |  |
| Andersson 2016 |  |  | x | x | x | x |  |  |  |  |  |  |  |  |  |  |  |  |  |  |  |  |  |  |  |
| Christensen 2014a |  |  |  |  |  |  | x |  |  |  |  |  |  |  |  |  |  |  |  |  |  |  |  |  |  |
| Christensen 2014b |  |  |  |  |  |  |  |  |  |  |  |  |  |  |  |  |  |  |  |  |  |  |  |  |  |
| Dahlin 2016 |  |  |  |  |  |  |  |  |  |  |  |  |  |  |  |  |  |  |  |  |  |  |  |  |  |
| Dear 2015 |  |  |  |  |  |  |  | x |  |  |  |  |  |  |  |  |  |  |  |  |  |  |  |  |  |
| Hazen 2009 |  |  |  |  |  |  |  |  |  |  |  |  |  |  |  |  |  |  |  |  |  |  |  |  |  |
| Hirsch 2018 |  |  |  |  |  |  |  |  |  |  |  |  |  |  |  |  |  |  |  |  |  |  |  |  |  |
| Howell 2018 |  |  |  |  |  |  |  |  |  |  |  |  |  |  |  |  |  |  |  |  |  |  |  |  |  |
| Johansson 2013 |  |  |  |  |  |  |  |  |  |  |  |  |  |  |  |  |  |  |  |  |  |  |  |  |  |
| Jones 2016 | x |  |  |  |  |  |  |  | x | x | x | x |  |  |  |  |  |  |  |  |  |  |  |  |  |
| Navarro-Haro 2019 |  |  |  |  |  |  |  |  |  |  |  |  | x | x | x |  |  |  |  |  |  |  |  |  |  |
| Paxling 2011 |  |  |  |  |  |  |  |  |  |  |  |  |  |  |  |  |  |  |  |  |  |  |  |  |  |
| Pham 2016 |  |  |  |  |  |  |  |  |  |  |  |  |  |  |  | x | x |  |  |  |  |  |  |  |  |
| Repetto 2013 |  |  |  |  |  |  |  |  |  |  |  |  |  |  |  |  |  | x |  |  |  |  |  |  |  |
| Richards 2016 |  |  |  |  |  |  |  |  |  |  |  |  |  |  |  |  |  |  | x |  |  |  |  |  |  |
| Robinson 2010 |  |  |  |  |  |  |  |  |  |  |  |  |  |  |  |  |  |  |  |  |  |  |  |  |  |
| Teng 2019 |  |  |  |  |  |  |  |  |  |  |  |  |  |  |  |  |  |  |  |  |  |  |  |  |  |
| Titov 2009 |  |  |  |  |  |  |  |  |  |  |  |  |  |  |  |  |  |  |  |  |  |  |  |  |  |
| Titov 2010 |  |  |  |  |  |  |  |  |  |  |  |  |  |  |  |  |  |  |  | x | x |  |  |  |  |
| Topper 2017 |  |  |  |  |  |  |  |  |  |  |  |  |  |  |  |  |  |  |  |  |  | x | x | x | x |
| **Total no of comparisons** | 1 | 1 | 1 | 1 | 1 | 1 | 1 | 1 | 1 | 1 | 1 | 1 | 1 | 1 | 1 | 1 | 1 | 1 | 1 | 1 | 1 | 1 | 1 | 1 | 1 |

**F3. Outcome acronyms explained**

ACES = Anxiety change expectancy scale; ASI-3 = Anxiety Sensitivity Index-3; BAI = Beck Anxiety Inventory; BBQ = Brunnsviken Brief Quality of Life Questionnaire; BDI = Beck Depression Inventory; BDI-II = Beck Depression Inventory-II; CAQ = Cognitive Avoidance Questionnaire; CBT = Cognitive Behaviour Therapy; CES-D = Center for Epidemiologic Studies Depression Scale; CGI = Clinical Global Improvement Scale; DASS-21 = Depression Anxiety Stress Scales; DERS=Difficulties in Emotion Regulation Scale; DMHI = Digital Mental Health Intervention; EDI-2-BU = Eating Disorder Inventory; FFMQ = Five Facet Mindfulness Questionnaire; GAD-7 = Generalised Anxiety Disorder Questionnaire-7 item; GADQ-IV = Generalized Anxiety Disorder Questionnaire-4 item; GAI = Geriatric Anxiety Inventory; GDS = Geriatric Depression Scale; HADS = Hospital Anxiety and Depression Scale; HADS-A = Hospital Anxiety and Depression Scale-Anxiety Subscale; HAM-A = Hamilton Anxiety Rating Scale; IOU = Intolerance of Uncertainty Scale; K-10 = Kessler Psychological Distress Scale; MADRS-S = Montgomery–Åsberg Depression Rating Scale; MAIA = Multidimensional assessment of interoceptive awareness; MASQ-D30 = Mood and Anxiety Symptoms Questionnaire-short form; MCQ-30 = Metacognition Questionnaire-30; MINI = Mini-International Neuropsychiatric Interview; MINI-SPIN = Mini-Social Phobia Inventory; NEO-FFI-N = NEO Five-Factor Inventory – 3; OASIS = Overall Anxiety Severity and Impairment Scale; PDSS-SR = Panic Disorder Severity Scale; PHQ-9 = Patient Health Questionnaire-9 item; PSWQ = Penn State Worry Questionnaire; PSWQ-A = Penn State Worry Questionnaire- abbreviated; PTQ = Perseverative Thinking Questionnaire; QDS = Quick Drinking Screen; QLES-Q-SF = Quality of Life Enjoyment and Satisfaction Questionnaire – Short Form; QOLI = Quality of Life Inventory; RRS = Ruminative response scale; SCID-I = Structured Clinical Interview for DSM-IV Axis I Disorders; SDS = Sheehan Disability Scale; SPSQ = Satisfaction with Performance Scaled Questionnaire; STAI = State-Trait Anxiety Inventory; STAI-S = State-Trait Anxiety Inventory- State; STAI-T = State-Trait Anxiety Inventory= Trait; WASA = Work and Social Adjustment; WHOQOL = The World Health Organization Quality of Life
