# Supplementary material for: Digital Interventions for Generalized Anxiety Disorder (GAD): Systematic Review and Network Meta-Analysis
Source: Front Psychiatry. 2021 Dec 6;12:726222. doi: 10.3389/fpsyt.2021.726222 (PMC8685377; doi:10.3389/fpsyt.2021.726222)
Supplement: Supplementary file 7 [file Data_Sheet_7.docx]

**Appendix H SUCRA GRAPHS AND RANKOGRAMS**

**H1. GAD-7 SUCRAS: Ranking of interventions based on SUCRA values for each intervention for GAD-7**

**H2. GAD-7 RANKOGRAMS: Rankogram of each intervention for GAD-7**
