# Supplementary material for: Digital Interventions for Generalized Anxiety Disorder (GAD): Systematic Review and Network Meta-Analysis
Source: Front Psychiatry. 2021 Dec 6;12:726222. doi: 10.3389/fpsyt.2021.726222 (PMC8685377; doi:10.3389/fpsyt.2021.726222)
Supplement: Supplementary file 8 [file Data_Sheet_8.docx]

**Appendix I Assessment of between-study heterogeneity and inconsistencies**

As only one study was performed in a milder population, the NMA ANCOVA RE meta-regression model considering a binary covariate on severity (1: mild/moderate; 0: moderate/severe) did not converge for neither of the endpoints. For comorbidities, the number of studies reporting information on the proportion of individuals within the trial with comorbidities was limited (GAD-7: 2 out of 13), impairing the use of multiple imputation. For this reason, we did not explore this variable further. Information on the proportion of individuals with concomitant medication was more prevalent across the evidence base (GAD-7: 9 out of 13). Thus, data on concomitant medication was included as a covariate in the synthesis modelling.

NMA model for GAD-7 that accounted for the proportion of patients receiving concomitant medication, fit comparably with those that did not, suggesting that no improvement in model fit was achieved. The effect modification coefficient is of the expected direction (GAD-7: $\beta_{med}$= -1.8 [-28.6 to 24.2]), suggesting that as the proportion of patients receiving concomitant medication increases, GAD-7 score is reduced. However, the covariate effect is not statistically significant and highly uncertain. When this covariate is included, the between-study heterogeneity parameter, $\tau^{2}$, is not reduced, suggesting that heterogeneity is not explained by this covariate. Crucially, even if the proportion receiving concomitant medication is found to be an important effect modifier, the described meta-regression model is not necessarily suited to detect this intervention-covariate interaction as patients were receiving medication before trial entry. Therefore, medication may have already exerted an effect on patients, being captured by the ANCOVA baseline adjustment component, $\beta_{k}$.

The consistency models produced lower DIC (> 3 points difference for the GAD-7 endpoint) than the inconsistency models and therefore the additional model complexity that is due to the consistency assumptions is supported by the data (Table I1). The consistency plots (Figure I1) show that there are a few deviant data points in which the inconsistency models lead to higher residual deviance than the consistency models, further supporting the latter.

**Table I1 – Results from ANCOVA RE NMA consistency and inconsistency models for GAD-7.**

| **Model** | **Dres** | **DIC** | **Tau.sq** | **B_base** |
| --- | --- | --- | --- | --- |
| **NMA ANCOVA RE – Consistency model** | 21.28  [11.23, 36.39] | 194.50 | 1.85  [0.004, 25.47] | -0.09  [-0.85, 0.68] |
| **NMA ANCOVA RE – Inconsistency model** | 23.11  [12.45, 38.73] | 198.17 | 1.95  [0.004, 29.37] | -0.08  [-1.77, 1.65] |

Note: RE=Random-effects; Dres=mean residual deviance;DIC=deviance information criteria; Tau.sq=between-study heterogeneity; B_base=coefficient relating to baseline score adjustment.

**Fig I1 - Consistency plot for GAD-7**
