# Supplementary material for: Digital Interventions for Generalized Anxiety Disorder (GAD): Systematic Review and Network Meta-Analysis
Source: Front Psychiatry. 2021 Dec 6;12:726222. doi: 10.3389/fpsyt.2021.726222 (PMC8685377; doi:10.3389/fpsyt.2021.726222)

**Appendix K Comparison of NMA results on GAD-7 and PSWQ for DIs**

To compare the results of the NMAs across both outcome measures, GAD-7 and PSWQ, and only for comparisons between DIs and their alternatives (rather than between alternatives, e.g. medication vs. group therapy), we present an abridged version of the NMA results in Table K1. The table shows the network comparisons and the direct pairwise comparisons between DIs and alternatives for post-treatment (12 weeks) on GAD-7 and PSWQ median scores (adjusted for baseline). Figure K1 shows the likelihood of DIs being ‘best’ among all comparators, based on ANCOVAs for scores at up-to-12-weeks (adjusted for baseline). Figure K2 shows the likelihood of DIs being ‘best’ among all comparisons between interventions, non-therapeutic controls and no intervention, based on SUCRAs.

**Table K1 – Abridged^#^ meta-analysis results: relative treatment effects based on network and direct pairwise comparisons between digital interventions and alternatives for post-treatment (3-12 weeks) on GAD-7 and PSWQ median scores (adjusted for baseline)**

| **COMPARATORS** | **GAD-7** | | **PSWQ** | |
| --- | --- | --- | --- | --- |
|  | **Network** | **Direct Pairwise** | **Network** | **Direct Pairwise** |
| **Medication** |  |  |  |  |
| M vs SDI | -3.18 [-11.32, 4.76] | -2.70* [-9.80, 4.40] | Not available | Not available |
| M vs UDI | -3.97 [-14.30, 6.20] | Not available | Not available | Not available |
| **No intervention** |  |  |  |  |
| SDI vs NI | -1.75 [-15.72, 12.26] | -3.66 [-8.19, 0.90] FE  -1.26** [-43.8, 40.93] RE | -6.43 [-39.63, 39.15] | -5.23 [-12.91, 2.39] FE  4.76** [-51.7, 59.53] RE |
| UDI vs. NI | -0.96 [-16.43, 14.54] | Not available | -7.13 [-37.55, 24.61] | Not available |
| **Group therapy** |  |  |  |  |
| SNoDI vs. SDI | -0.65 [-14.67, 13.23] | -0.71* [-14.03, 12.61] | -0.71 [-25.34, 23.90] | -0.58* [-24.74, 23.58] |
| SNoDI vs UDI | -1.45 [-16.92, 14.03] | Not available | -0.06 [-34.59, 34.83] | Not available |
| **Non-therapeutic controls** |  |  |  |  |
| **Digital** |  |  |  |  |
| SDI vs SDC | -1.61 [-8.03, 4.82] | -8.16 [-26.57, 13.04] FE  -1.07** [-30.2, 27.19] RE | -2.65 [-15.95, 10.74] | -2.93 [-20.90, 15.00] FE  0.95** [-77.24, 80.71] RE |
| SDI vs UDC | -1.49 [-10.51, 7.47] | -1.50* [-11.34, 8.34] | -4.35 [-34.27, 25.60] | -3.60* [-35.42, 28.22] |
| UDI vs SDC | -0.83 [-8.82, 7.29] | -0.51 [-31.75, 28.95] | -3.36 [-28.72, 21.82] | -2.52 [-26.55, 22.31] |
| UDI vs UDC | -0.70 [-9.10, 7.58] | -0.77 [-7.81, 6.26] | -4.96 [-35.07, 24.94] | -6.73 [-33.99, 21.37] |
| **Non-digital** |  |  |  |  |
| SNoDC vs SDI | Not available | Not available | 6.15 [-18.36, 30.39] | 6.75* [-20.97, 34.47] |
| SNoDC vs UDI | Not available | Not available | 6.81 [-26.18, 39.67] | Not available |
| **Variants of digital interventions** |  |  |  |  |
| SDI vs UDI | -0.79 [-7.39, 5.80] | -0.14 [-8.85, 8.89] FE  -1.38 [-26.23, 23.62] RE | 0.67 [-23.71, 25.41] | 1.53 [-21.48, 24.93] FE |

# only comparisons that include digital interventions are presented

* Non-pooled data for when n=1

**Pairwise ANCOVA RE meta-analysis for when n>3 for contrasts with intervention SDI

**Fig. K1 - Ranking of interventions and controls, and likelihood of being ‘best’ compared to no intervention (NI) based on ANCOVAs for scores at 12-weeks (adjusted for baseline)**


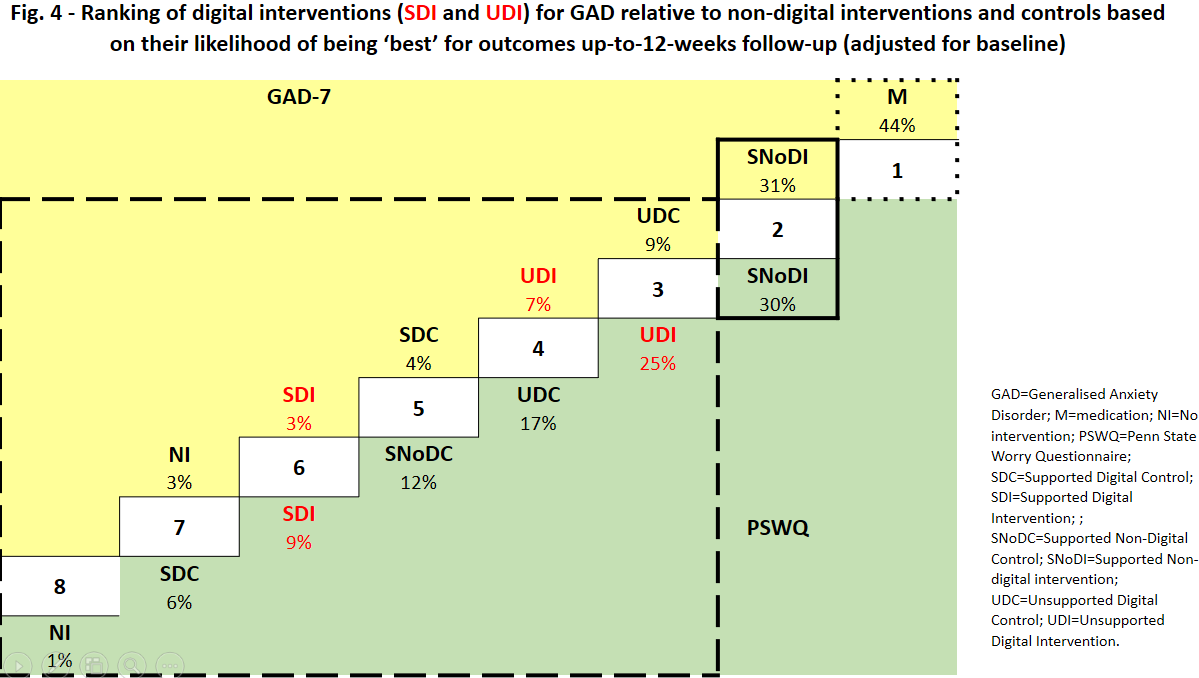


**Fig. K2 - Ranking of interventions and controls, and likelihood of being ‘best’, based on SUCRAs**


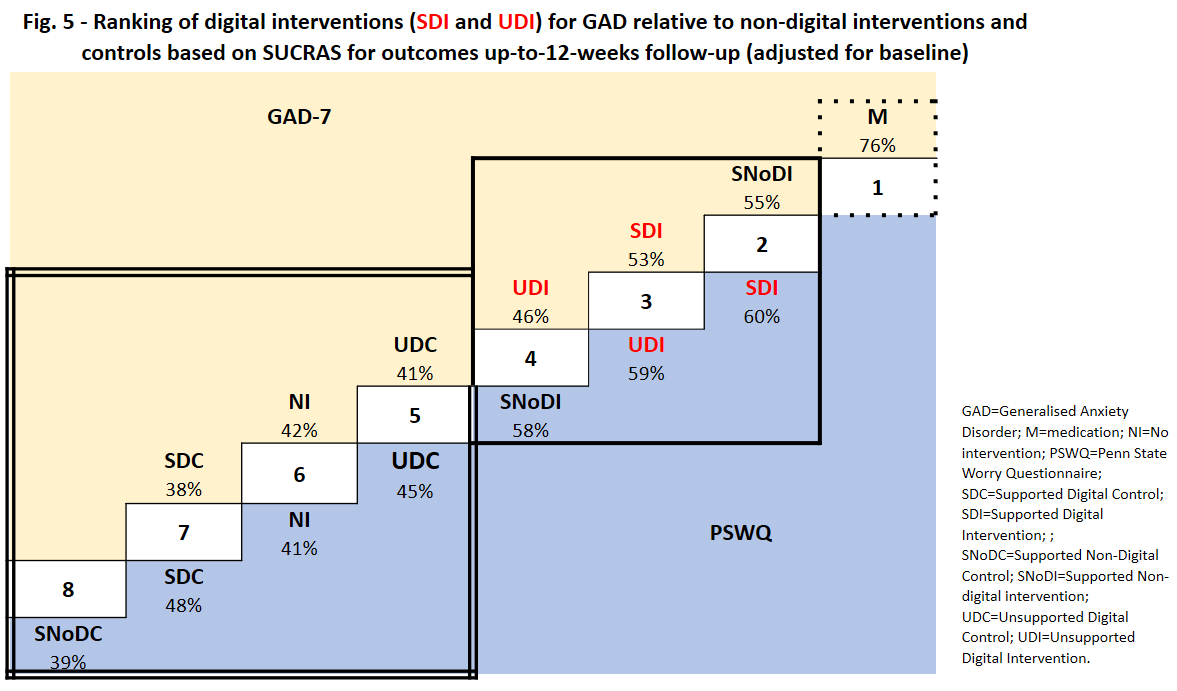

Supplement: Supplementary file 9 [file Data_Sheet_9.docx]
