# Supplementary material for: Digital Interventions for Generalized Anxiety Disorder (GAD): Systematic Review and Network Meta-Analysis
Source: Front Psychiatry. 2021 Dec 6;12:726222. doi: 10.3389/fpsyt.2021.726222 (PMC8685377; doi:10.3389/fpsyt.2021.726222)
Supplement: Supplementary file 10 [file Data_Sheet_10.docx]

**Appendix G Direct pairwise comparisons reported in RCTs of Digital Interventions for GAD**

**Table G1 - Direct pairwise comparisons reported in RCTs of Digital Interventions for GAD on GAD-7 scores**

| **Studies** | **RCT design** | **Randomisation arms** | **SDI vs. NI** | **SDI vs. SNoDI** | **SDI vs. M** | **SDI vs. UDC** | **SDI vs. SDC** | **UDI vs. UDC** | **UDI vs. SDC** | **SDI vs. UDI** | **M vs. SDC** | **UDC vs. SDC** |
| --- | --- | --- | --- | --- | --- | --- | --- | --- | --- | --- | --- | --- |
| Christensen 2014a | 5-arm | SDI vs UDC vs SDC vs UDI1 (no reminders) vs UDI2 (weekly reminders) |  |  |  | √ | √ | √ √ | √ √ | √ √ |  | √ |
| Christensen 2014b | 3-arm | SDI vs SDC vs M (SSRI) |  |  | √ |  | √ |  |  |  | √ |  |
| Dahlin 2016 | 2-arm | SDI vs NI | √ |  |  |  |  |  |  |  |  |  |
| Dear 2015 | 2x2 factorial | SDI (CBT Transdiagnostic + GAD-specific) vs UDI (CBT Transdiagnostic + GAD-specific) |  |  |  |  |  |  |  | √ |  |  |
| Hirsch 2018 | 3-arm | SDI ( with + without RNT priming) vs SDC |  |  |  |  | √ |  |  |  |  |  |
| Johansson 2013 | 2-arm | SDI vs SDC |  |  |  |  | √ |  |  |  |  |  |
| Jones 2016 | 2-arm | SDI vs NI | √ |  |  |  |  |  |  |  |  |  |
| Navarro-Haro 2019 | 2-arm | SDI vs SNoDI |  | √ |  |  |  |  |  |  |  |  |
| Pham 2016 | 2-arm | UDI vs UDC |  |  |  |  |  | √ |  |  |  |  |
| Repetto 2013 | 3-arm | SDI1 (with biofeed) vs SDI2 (no biofeed) vs NI | √ √ |  |  |  |  |  |  |  |  |  |
| Richards 2016 | 2-arm | SDI vs NI | √ |  |  |  |  |  |  |  |  |  |
| Robinson 2010 | 3-arm | SDI 1 (clinicians) vs SDI2 (assistants) vs NI | √ √ |  |  |  |  |  |  |  |  |  |
| Titov 2009 | 2-arm | SDI vs NI | √ |  |  |  |  |  |  |  |  |  |
| **13 studies** |  |  | 8 | 1 | 1 | 1 | 4 | 3 | 2 | 3 | 1 | 1 |

GAD=Generalised Anxiety Disorder; M=medication; NI=No intervention; PSWQ=Penn State Worry Questionnaire; SDC=Supported Digital Control; SDI=Supported Digital Intervention; ; SNoDC=Supported Non-Digital Control; SNoDI=Supported Non-digital intervention; UDC=Unsupported Digital Control; UDI=Unsupported Digital Intervention.

RNT repetitive negative thinking

*Hirsch – 3-arm but the results were reported in 2 groups by pooling the intervention groups

**Table G2 - Results of RCTs using the GAD-7 as outcome synthesised in the GAD-7 NMA model.**

| **Studies** | **Intervention** | **N** | **Baseline GAD (y_0_, mean)** | **Baseline GAD (se_0_, SE)** | **Post-treatment GAD (y_1_, mean)** | **Post-treatment GAD (se_1_, SE)** |
| --- | --- | --- | --- | --- | --- | --- |
| Christensen 2014a^158^ | UDC | 111 | 7.00 | 3.80 | 6.10 | 4.10 |
|  | SDC | 113 | 6.60 | 3.70 | 5.30 | 4.20 |
|  | UDI | 111 | 6.80 | 3.90 | 6.10 | 4.70 |
|  | UDI | 110 | 6.80 | 3.60 | 4.70 | 3.60 |
|  | SDI | 113 | 6.20 | 3.90 | 4.60 | 2.90 |
| Christensen 2014b^159^ | SDC | 7 | 11.70 | 4.80 | 12.00 | 6.50 |
|  | SDI | 8 | 11.50 | 3.70 | 6.50 | 2.30 |
|  | M | 6 | 14.80 | 5.20 | 3.80 | 2.80 |
| Dahlin 2016^40^ | NI | 51 | 13.51 | 4.14 | 10.72 | 4.20 |
|  | SDI | 52 | 13.83 | 3.66 | 6.90 | 3.52 |
| Dear 2015^160^ | UDI | 170 | 12.42 | 4.34 | 6.23 | 4.05 |
|  | SDI | 168 | 12.61 | 4.40 | 6.09 | 3.96 |
| *Hirsch 2018^162^ | SDC | 20 | 14.55 | 3.46 | 11.15 | 4.33 |
|  | SDI | 44 | 14.00 | 3.18 | 11.59 | 4.91 |
| Johansson 2013^35^ | SDC | 21 | 12.67 | 2.80 | 8.90 | 4.70 |
|  | SDI | 22 | 12.23 | 3.80 | 6.95 | 5.30 |
| Jones 2016^164^ | NI | 21 | 11.99 | 4.82 | 10.16 | 4.22 |
|  | SDI | 24 | 11.78 | 4.87 | 6.50 | 4.55 |
| Navarro-Haro 2019^165^ | SDI | 19 | 14.05 | 4.61 | 9.79 | 5.60 |
|  | SNoDI | 20 | 15.33 | 4.03 | 9.08 | 3.85 |
| Pham 2016^37^ | UDC | 32 | 10.66 | 4.63 | 9.53 | 4.79 |
|  | UDI | 31 | 11.55 | 5.05 | 9.39 | 5.21 |
| Repetto 2013^167^ | NI | 4 | 14.25 | 4.57 | 8.75 | 6.19 |
|  | SDI | 4 | 10.25 | 5.56 | 8.25 | 3.95 |
|  | SDI | 4 | 16.00 | 8.37 | 6.50 | 4.51 |
| Richards 2016^168^ | NI | 67 | 13.19 | 2.78 | 9.13 | 4.13 |
|  | SDI | 70 | 12.84 | 2.39 | 7.73 | 4.44 |
| Robinson 2010^169^ | NI | 48 | 12.94 | 4.07 | 11.25 | 4.70 |
|  | SDI | 50 | 11.90 | 3.38 | 6.02 | 3.43 |
|  | SDI | 47 | 12.45 | 4.14 | 5.55 | 4.73 |
| Titov 2009^171^ | NI | 21 | 13.62 | 3.51 | 12.29 | 4.26 |
|  | SDI | 24 | 14.33 | 4.50 | 6.92 | 4.40 |

GAD=Generalised Anxiety Disorder; M=medication; NI=No intervention; SDC=Supported Digital Control; SDI=Supported Digital Intervention; ; SNoDC=Supported Non-Digital Control; SNoDI=Supported Non-digital intervention; UDC=Unsupported Digital Control; UDI=Unsupported Digital Intervention.

*Hirsch – 3-arm but the results were reported in 2 groups by pooling the intervention groups.
