# Supplementary material for: Digital Interventions for Generalized Anxiety Disorder (GAD): Systematic Review and Network Meta-Analysis
Source: Front Psychiatry. 2021 Dec 6;12:726222. doi: 10.3389/fpsyt.2021.726222 (PMC8685377; doi:10.3389/fpsyt.2021.726222)
Supplement: Supplementary file 11 [file Data_Sheet_11.docx]

**Appendix J Penn State Worry Questionnaire**

**Table J1 - Direct pairwise comparisons reported in RCTs of Digital Interventions for GAD on PSWQ scores**

| **Studies** | **RCT design** | **Randomisation arms** | **SDI vs. NI** | **SDI**  **vs. SNoDI** | **SDI**  **vs. UDC** | **SDI**  **vs.**  **SDC** | **SDI**  **vs. SNoDC** | **UDI**  **vs. UDC** | **UDI**  **vs.**  **SDC** | **SDI**  **vs.**  **UDI** | **UDC**  **vs.**  **SDC** | **SNoDI vs.**  **NI** | **SNoDC**  **vs.**  **SDC** |
| --- | --- | --- | --- | --- | --- | --- | --- | --- | --- | --- | --- | --- | --- |
| Andersson 2012 | 3-arm | SDI1 (CBT) vs SDI2 (psychodynamic) vs NI | √ √ |  |  |  |  |  |  |  |  |  |  |
| Andersson 2016 | 2-arm | SDI vs NI | √ |  |  |  |  |  |  |  |  |  |  |
| Christensen 2014a | 5-arm | SDI vs UDC vs SDC vs UDI1 (no reminders) vs UDI2 (weekly reminders) |  |  | √ | √ |  | √ √ | √ √ | √ √ | √ |  |  |
| Dahlin 2016 | 2-arm | SDI vs NI | √ |  |  |  |  |  |  |  |  |  |  |
| Hazen 2009 | 2-arm | SDI vs SDC |  |  |  | √ |  |  |  |  |  |  |  |
| Hirsch 2018 | 3-arm | SDI ( with + without RNT priming) vs SDC |  |  |  | √ |  |  |  |  |  |  |  |
| Paxling 2011 | 2-arm | SDI vs NI | √ |  |  |  |  |  |  |  |  |  |  |
| Repetto 2013 | 3-arm | SDI1 (with biofeed) vs SDI2 (no biofeed) vs NI | √ √ |  |  |  |  |  |  |  |  |  |  |
| Richards 2016 | 2-arm | SDI vs NI | √ |  |  |  |  |  |  |  |  |  |  |
| Robinson 2010 | 3-arm | SDI 1 (clinicians) vs SDI2 (assistants) vs NI | √ √ |  |  |  |  |  |  |  |  |  |  |
| Teng 2019 | 3-arm | SDI vs SDC vs SNoDC |  |  |  | √ | √ |  |  |  |  |  | √ |
| Titov 2009 | 2-arm | SDI vs NI | √ |  |  |  |  |  |  |  |  |  |  |
| Titov 2010 | 2-arm | SDI vs NI | √ |  |  |  |  |  |  |  |  |  |  |
| Topper 2017 | 2-arm | SDI vs SNoDI | √ | √ |  |  |  |  |  |  |  | √ |  |
| **14 studies** |  |  | 13 | 1 | 1 | 4 | 1 | 2 | 2 | 2 | 1 | 1 | 1 |

**Table J2 - Results of RCTs using the PSWQ as outcome synthesised in the PSWQ NMA model**

| **Studies** | **Intervention** | **N** | **Baseline PSWQ**  **(y_0_, mean)** | **Baseline PSWQ**  **(se_0_, SE)** | **Post-treatment PSWQ (y_1_, mean)** | **Post-treatment PSWQ (se_1_, SE)** |
| --- | --- | --- | --- | --- | --- | --- |
| Andersson 2012^157^ | NI | 27 | 68.52 | 6.24 | 62.88 | 9.39 |
|  | SDI | 27 | 67.89 | 6.19 | 60.78 | 9.83 |
|  | SDI | 27 | 69.74 | 5.56 | 61.88 | 7.73 |
| Andersson 2016^39^ | NI | 70 | 66.59 | 6.84 | 66.31 | 7.84 |
|  | SDI | 70 | 65.60 | 6.20 | 52.92 | 11.16 |
| Christensen 2014a^158^ | UDC | 111 | 40.30 | 12.00 | 41.00 | 12.30 |
|  | SDC | 113 | 39.20 | 10.80 | 38.40 | 12.80 |
|  | UDI | 111 | 40.50 | 12.20 | 39.00 | 13.20 |
|  | UDI | 110 | 37.90 | 12.50 | 33.80 | 11.50 |
|  | SDI | 113 | 39.50 | 11.60 | 37.40 | 10.60 |
| Dahlin 2016^40^ | NI | 51 | 67.45 | 6.77 | 63.35 | 8.4 |
|  | SDI | 52 | 66.88 | 7.16 | 55.29 | 10.02 |
| Hazen 2009^161^ | SDC | 12 | 67.96 | 6.05 | 67.83 | 8.05 |
|  | SDI | 12 | 71.09 | 4.70 | 62.82 | 8.75 |
| *Hirsch 2018^162^ | SDC | 20 | 67.10 | 6.54 | 65.80 | 6.84 |
|  | SDI | 44 | 69.48 | 6.22 | 65.32 | 9.39 |
| Paxling 2011^166^ | NI | 45 | 69.32 | 6.55 | 69.39 | 7.06 |
|  | SDI | 44 | 68.74 | 5.94 | 57.82 | 13.01 |
| Repetto 2013^167^ | NI | 4 | 51.25 | 9.85 | 50.00 | 5.29 |
|  | SDI | 4 | 48.50 | 12.66 | 47.25 | 8.73 |
|  | SDI | 4 | 41.25 | 13.24 | 48.50 | 12.40 |
| Richards 2016^168^ | NI | 67 | 63.48 | 6.95 | 60.33 | 8.79 |
|  | SDI | 70 | 63.04 | 8.11 | 58.53 | 10.97 |
| Robinson 2010^169^ | NI | 48 | 65.81 | 10.24 | 64.22 | 11.81 |
|  | SDI | 50 | 63.12 | 9.46 | 52.28 | 10.73 |
|  | SDI | 47 | 64.02 | 9.27 | 51.45 | 12.28 |
| Teng 2019^36^ | SDC | 31 | 60.60 | 10.09 | 57.03 | 8.23 |
|  | SDI | 31 | 59.80 | 8.86 | 53.43 | 11.01 |
|  | SNoDC | 31 | 62.27 | 8.99 | 60.18 | 8.88 |
| Titov 2009^171^ | NI | 21 | 66.33 | 12.70 | 66.14 | 8.70 |
|  | SDI | 24 | 66.13 | 8.25 | 56.75 | 10.78 |
| Topper 2017^172^ | NI | 85 | 59.15 | 6.78 | 57.80 | 8.54 |
|  | SDI | 84 | 58.73 | 6.96 | 51.87 | 8.85 |
|  | SNoDI | 82 | 58.20 | 6.59 | 51.29 | 8.58 |

PSWQ=Penn State Worry Questionnaire; M=medication; NI=No intervention; SDC=Supported Digital Control; SDI=Supported Digital Intervention; SNoDC=Supported Non-Digital Control; SNoDI=Supported Non-digital intervention; UDC=Unsupported Digital Control; UDI=Unsupported Digital Intervention.

*Hirsch – 3-arm but the results were reported in 2 groups by pooling the intervention groups

**Fig. J1 - Network plot for comparisons between all interventions and controls for GAD populations in RCTs with PSWQ scores as an outcome**


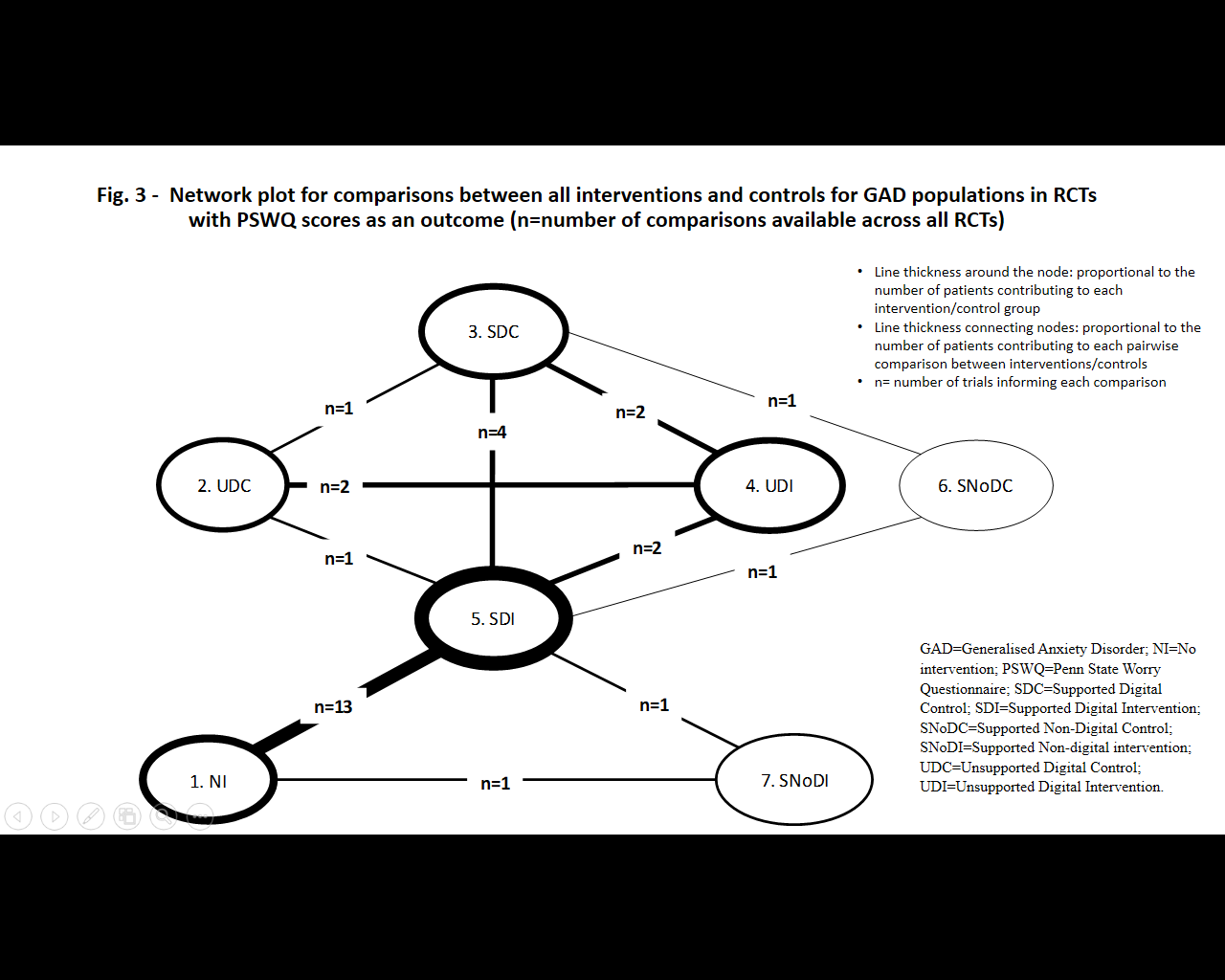


**Table J3 - Full meta-analysis results: network and direct pairwise comparisons between all interventions and controls for GAD post-treatment (12 weeks) on PSWQ Scores adjusted for baseline**

|  |  | **PSWQ Direct Pairwise^*,**^** | | | | | | | |
| --- | --- | --- | --- | --- | --- | --- | --- | --- | --- |
|  | **Comparator** | **NI** | **UDC** | **SDC** | **UDI** | **SDI** | | **SNoDC** | **SNoDI** |
|  |  |  |  |  |  | **FE** | **RE** |  |  |
| **PSWQ Network** | **NI** |  | Not available | Not available | Not available | -5.23  [-12.91, 2.39] | 4.76**  [-51.7, 59.53] | Not available | -6.51*  [-30.24, 17.22] |
|  | **UDC** | -2.13  [-45.14, 41.05] |  | -2.60*  [-37.39, 32.19] | -6.73  [-33.99, 21.37] | -3.60*  [-35.42, 28.22] | | Not available | Not available |
|  | **SDC** | -3.76  [-46.77, 32.38] | -1.76  [-32.27, 28.68] |  | -2.52  [-26.55, 22.31] | -2.93  [-20.90, 15.00] | 0.95**  [-77.24, 80.71] | 3.15*  [-20.58, 26.88] | Not available |
|  | **UDI** | -7.13  [-37.55, 24.61] | -4.96  [-35.07, 24.94] | -3.36  [-28.72, 21.82] |  | 1.53  [-21.48, 24.93] | --- | Not available | Not available |
|  | **SDI** | -6.43  [-39.63, 39.15] | -4.35  [-34.27, 25.6] | -2.65  [-15.95, 10.74] | 0.67  [-23.71, 25.41] |  | | 6.75*  [-20.97, 34.47] | -0.58*  [-24.74, 23.58] |
|  | **SNoDC** | -0.39  [-53.64, 39.29] | 1.87  [-35.41, 38.99] | 3.51  [-19.87, 26.64] | 6.81  [-26.18, 39.67] | --- | 6.15  [-18.36, 30.39] |  | Not available |
|  | **SNoDI** | -7.14  [-32.27, 28.68] | -4.98  [-44.2, 33.81] | -3.42  [-31.40, 24.76] | -0.06  [-34.59, 34.83] | --- | -0.710  [-25.34, 23.9] | -6.91  [-41.53, 27.92] |  |

FE=Fixed Effects; GAD=Generalised Anxiety Disorder; NI=No intervention; PSWQ=Penn State Worry Questionnaire; RE=Random Effects; SDC=Supported Digital Control; SDI=Supported Digital Intervention; SNoDC=Supported Non-Digital Control; SNoDI=Supported Non-digital intervention; UDC=Unsupported Digital Control; UDI=Unsupported Digital Intervention.

*Non-pooled data for when n=1

**Pairwise ANCOVA RE meta-analysis for when n>3.

Lower left triangle: Network Meta-Analysis Results (ANCOVA RE); Upper right triangle (shaded area): Direct Pairwise Meta-Analysis Results (ANCOVA FE); Cells with thick black perimeter: available comparisons between digital interventions (supported and unsupported) and alternatives

* FE with no Baseline adjustment, **Large tau^2 implying substantial heterogeneity across trials

**Fig. J2 - PSWQ SUCRAS: Ranking of interventions based on SUCRA values for each intervention for PSWQ.**

**Fig. J3 - PSWQ RANKOGRAMS: Rankogram of each intervention for PSWQ.**

**Table J4 – Results from ANCOVA RE NMA consistency and inconsistency models for PSWQ.**

| **Model** | **Dres** | **DIC** | **Tau.sq** | **B_base** |
| --- | --- | --- | --- | --- |
| **NMA ANCOVA RE – Consistency model** | 21.33  [11.34, 36.33] | 253.82 | 8.03  [0.02, 77.54] | 0.01  [-0.45, 0.48] |
| **NMA ANCOVA RE – Inconsistency model** | 21.58  [11.61, 36.51] | 254.27 | 7.06  [0.01, 73.4] | -0.01  [-1.13, 1.13] |

Note: RE=Random-effects; Dres=mean residual deviance;DIC=deviance information criteria; Tau.sq=between-study heterogeneity; B_base=coefficient relating to baseline score adjustment.

**Fig J4 - Consistency plot for PSWQ**
